# Bismuth Radical Catalysis: Thermally Induced Intramolecular C(sp^3^)–C(sp) Cyclization of Unactivated Alkyl Iodides and Alkynes

**DOI:** 10.1021/acscatal.5c02812

**Published:** 2025-08-13

**Authors:** Sebastián Martínez, Marius A. Junghanns, Tobias Dunaj, Crispin Lichtenberg

**Affiliations:** Department of Chemistry, 9377 Philipps-University Marburg, 35032 Marburg, Germany

**Keywords:** bismuth, transition metal bismuthanes, redox-neutral coupling, thermal catalysis, radical chemistry

## Abstract

Bismuth radical chemistry has attracted increased interest in recent years. Here, we report a series of bismuth–manganese complexes that catalyze the intramolecular redox-neutral coupling of alkyl iodides and alkynes under purely thermal conditions. Computational studies support the design of catalyst candidates, aiming to achieve low Bi–Mn bond homolytic dissociation energies. The radical nature of the cyclization reaction was supported by EPR spectroscopic spin trap experiments. This underexplored mode of thermally induced radical reactivity was applied to construct a variety of cyclic vinyl iodide compounds, including drug derivatives.

## Graphical abstract

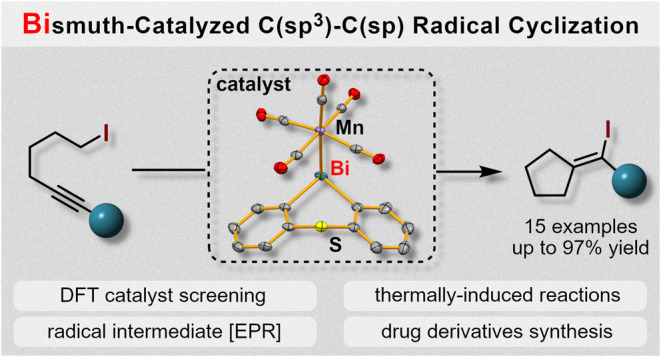

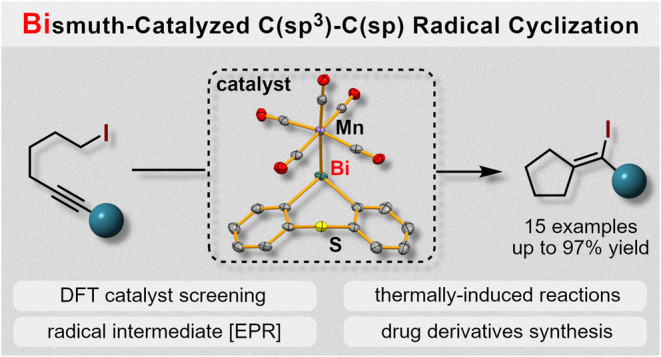

## Introduction

The development of novel catalytic methodologies involving bismuth has recently attracted significant attention in organic synthesis and catalysis, owing to its unique reactivity and versatility in both one- and two-electron catalytic processes.^1–4^ Bismuth-mediated radical transformations have re-emerged as particularly effective methodologies in the context of controlled radical reactions aimed at C–X bond formation (X = B, C, N, S).^3–6^ In this regard, bismuth complexes featuring low homolytic bond dissociation energies of Bi–X bonds (X = H, alkyl, allyl, aryloxide, amide, transition metal)^7–14^ have enabled applications in synthetic chemistry,^15^ polymerization catalysis,^16–18^ and organic synthesis.^11–14,19^ Despite significant advancements, the full potential of bismuth complexes in radical chemistry remains largely underexplored.

Atom transfer radical cyclization (ATRC) and atom transfer radical addition (ATRA)^20–26^ reactions represent efficient strategies to quickly furnish complex cyclic structures while maintaining an excellent overall atom economy of the process. Classical ATRC/ATRA methodologies required the use of stoichiometric amounts of initiators to form radicals from the alkyl halide starting materials (including organotin reagents,^22–25^ BEt_3_,^27–29^ or oxidants^26^). Recently, metal-based photoredox catalysis driven by visible light or blue LEDs^30–33^ has further improved the performance of ATRC/ATRA methodologies while operating under milder reaction conditions. In addition to significant advances, conventional photochemical approaches often struggle to convert unactivated alkyl halides due to their highly negative redox potentials, which often require reaction conditions that promote unwanted side reactions, such as parasitic hydrogen atom transfer (HAT) reactions. Therefore, we envisioned that developing a purely thermally induced, redox-neutral strategy for ATRC reactions would provide a complementary approach to existing state-of-the-art photochemical methodologies for such reactions (Scheme 1).^33,34^

**Scheme 1:**
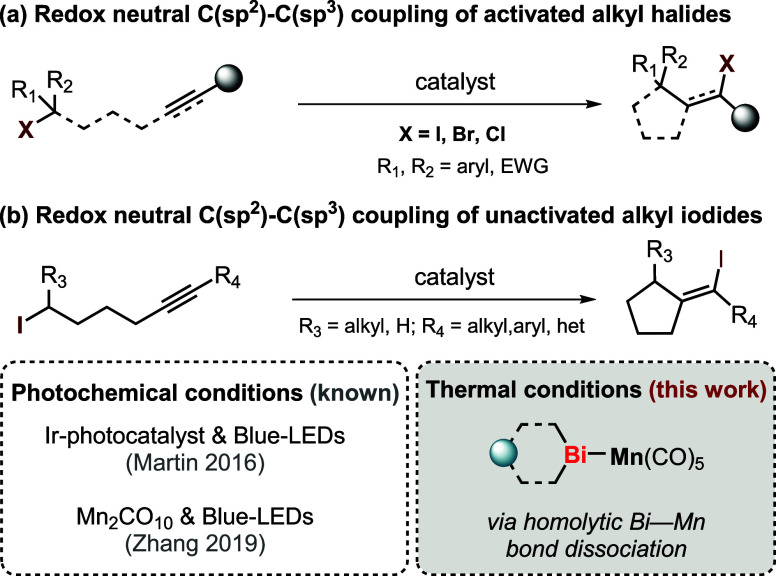
Context of This Work

Inspired by our previous work on thermally induced cyclo-isomerization reactions catalyzed by transition metal bismuthanes,^11^ we considered that a bismuth–manganese (Bi–Mn) complex could serve as an efficient catalyst for thermally induced ATRC of unactivated alkyl halides with alkynes. Herein, we report the first extensive set of examples of such a reaction operating via purely thermal initiation. Using a novel Bi–Mn complex, this type of transformation is catalyzed at mild temperatures, bypassing the need for light irradiation or external redox reagents. The reaction proceeds with excellent functional group tolerance and regioselectivity, highlighting the potential of Bi–Mn complexes as a new class of catalysts for such radical transformations under simple thermal conditions.

## Results and Discussion

We commenced our investigation by computing the homolytic bond dissociation energies of potential Bi–Mn catalysts, aiming to identify candidates with weak Bi–Mn bonds. In view of the complex catalytic scenario of the target reaction, a weak Bi–Mn bond would definitely not guarantee a high catalytic activity. But Bi–Mn homolysis is expected to be the key first step in the catalytic process and was hypothesized to be a simple, computationally affordable parameter with a high chance of correlating with a catalytic performance when compared to other Bi/Mn species (as these would all lead to Mn–I and Bi–C bond formation). Our study considered 15 different structures, including both reported compounds and unknown complexes, that show realistic chances of being readily synthesizable. The computed homolytic bond dissociation energies are presented in Figure 1. Our calculations show that bond dissociation energies in the range of 26.4–32.1 kcal·mol^–1^ are covered. The most promising candidates, namely, compounds **2**, **3**, and **4**, represent simple six-membered bisma-cyclic structures without extensive steric load or electronic stabilization (Figure 1, highlighted in red).

**Figure 1:**
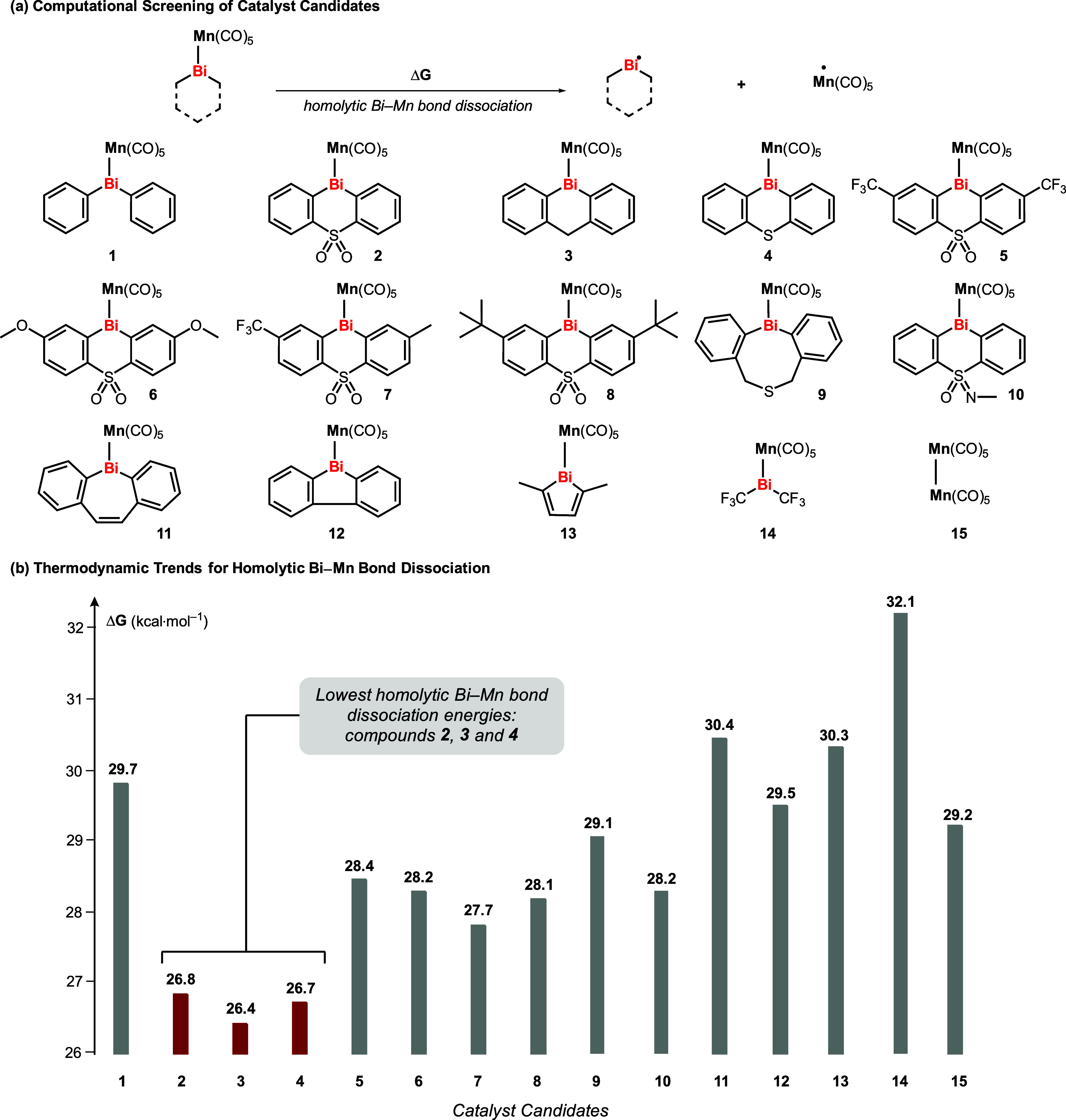
Identification of suitable catalyst candidates by DFT studies.

Notably, the calculated bond dissociation energies of these compounds are significantly lower than those of compounds **1** and **9**, which have been experimentally investigated by our group for related cyclo-isomerization reactions.^11^

With the promising candidates selected, we followed up to synthesize all three unreported compounds. Starting from the corresponding bismuth iodides **2**-**I**, **3**-**I**, and **4**-**I**, compounds **2**, **3**, and **4** were prepared and isolated in yields of 57%, 52%, and 82%, respectively (Scheme 2). Single-crystal X-ray diffraction (XRD) analyses of all three compounds confirmed the targeted structures (**2** and **4** are shown in Scheme 2; **3** is shown in the Supporting Information). Compound [(Κ^2^-C_12_H_8_SO_2_)­Bi­(Mn­(CO)_5_)]·(MeCN) (**2**) crystallized in the triclinic space group *P*1̅ with *Z* = 2. Compound [(K^2^- C_12_H_8_S)­Bi­(Mn­(CO)_5_)] (**4**) crystallized in orthorhombic space group *Pccn* with Z = 8. The bismuth centers are found in a trigonal pyramidal coordination geometry. The Bi–Mn bond lengths for **2** and **4** are 2.85 Å and 2.84 Å, respectively, which are in the expected range of interatomic distances.^11,35–38^ The heteroatom bridging unit is oriented toward the bismuth atom in **2** and toward the Mn­(CO)_5_ complex fragment in **4** (side view in Scheme 2b,c).

**Scheme 2:**
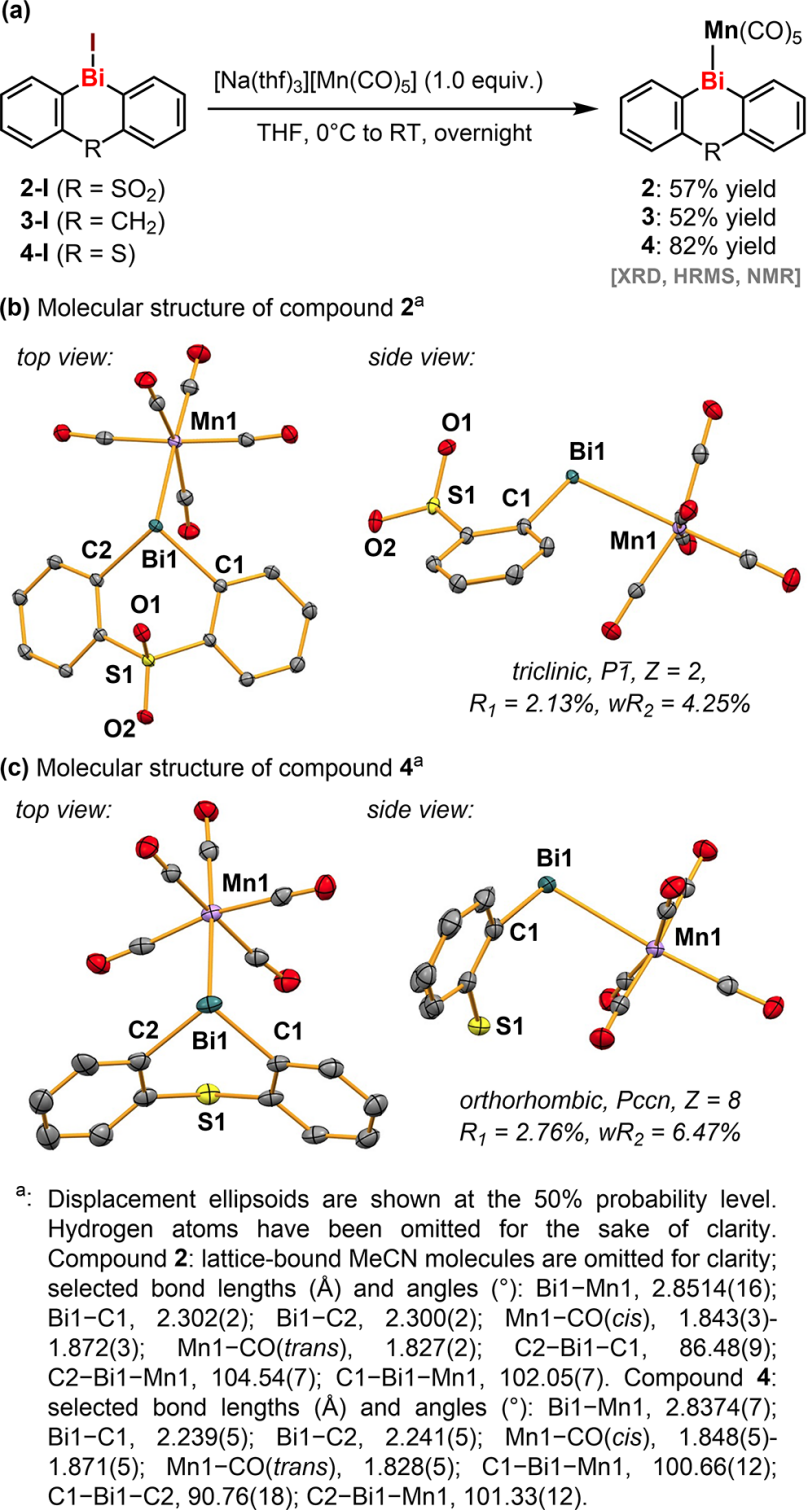
Synthesis and Molecular Structures of Catalyst Candidates

Exploratory studies to test the catalytic activity of the prepared candidates on the known cyclo-isomerization of 6-iodo-1-hexene showcased that compound **4** was significantly more active than our previously reported catalyst **1**,^11^ leading to excellent yields in much lower reaction times (see Supporting Information for further details and comparison of all candidate’s reactivity).

We then evaluated the performance of catalyst candidates **2**, **3**, and **4**, along with compounds **1** and **15**, in the model ATRC of alkyl iodide **16a** under thermal conditions (Table 1). Compound **4** effectively promoted the cyclization reaction, producing excellent yields of **16b** after 20 h at 80 °C (entry 1). With lower catalyst loadings of 5%, full conversion to cyclization product **16b** could still be achieved, albeit at significantly longer reaction times (see Supporting Information). Lower reaction temperatures progressively decrease the yield (entries 2 and 3). Adjusting the reaction time was crucial for full substrate conversion (entry 4). Variation of the reaction concentration had minimal impact on the yield (entry 5). However, exposure to UV light or blue LED significantly reduced the yields (entries 6 and 7), most likely as a result of promoting side reactions.

**Table 1:**
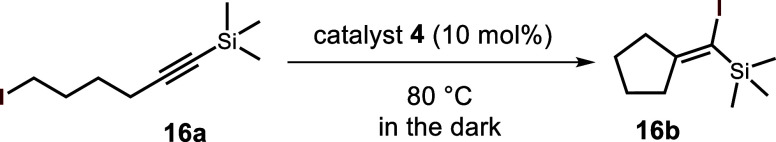
Performance of Catalyst Candidates and Optimization of Model Reaction Conditions

entry	deviation from standard conditions^a^	16b (%)^a^
1	none	91
2	70 °C	82
3	60 °C	76
4	1 h instead of 20 h	37
5	0.05 M instead of 0.10 M	84
6	blue LED, 1 h, rt	36
7	UV light^c^, 1 h, rt	44
8	3 instead of 4	68
9	2 instead of 4	40
10	1 instead of 4	42
11	15 instead of 4	5
12	15 instead of 4, ambient light	11

a **16a** (0.025 mmol), C_6_D_6_ (0.1 M), and **4** (10 mol %).

b Determined by^1^H NMR using 1,3,5-trimethoxybenzene as an internal standard.

c Low-pressure Hg-vapor lamp.

Compound **3** also proved to be a successful catalyst, albeit with slightly lower efficiency, which was ascribed to side reactions at the double-benzylic position in the ligand backbone (entry 8). In contrast, compound **2** produced low yields (entry 9), likely due to its limited solubility in benzene caused by the sulfone group in the ligand backbone. Catalyst **1** performed notably worse than compounds **4** and **2**, as judged on the basis of low yields of the desired cyclization product **16b** (entry 10). Control experiments confirmed that compound **15** did not promote the reaction under thermal conditions (in the dark) or under ambient light (entries 11–12). Finally, the performance achieved with compound **4** (entry 1) is similar to those reported under photochemical conditions using Sn reagents,^25^ Ir photocatalysts,^33^ or Mn_2_(CO)_10_.^34^ It outperforms previously reported BEt_3_/O_2_-based systems (one example: 30 mol % catalyst, 83% yield, secondary (i.e., more reactive) alkyl iodide substrate).^28^ Therefore, the above-mentioned findings highlight the success of our approach to identify suitable catalysts for thermally induced ATRC reactions from a set of structurally related compounds.

Next, we gathered evidence of radical species being formed during the model reaction. For that, the ATRC reaction of substrate **16a** catalyzed by **4** was performed in the presence of nitrone **17** as a radical trap at 70 °C and analyzed by EPR spectroscopy at room temperature. The observed resonance shows coupling constants of a­(^14^N) = 41.0 MHz and a­(^1^H) = 9.22 MHz at a *g*_iso_ value of 2.0049 (Figure 2, top). Importantly, the strong signal intensity allowed for the identification of additional hyperfine coupling with nuclei of low natural abundance, a­(^13^C) = 30.0 MHz and a­(^29^Si) = 14.3 MHz (Figure 2, bottom). Such parameters agree with those expected for radical species **18**, indicating the presence of already cyclized radical species in the reaction.

**Figure 2:**
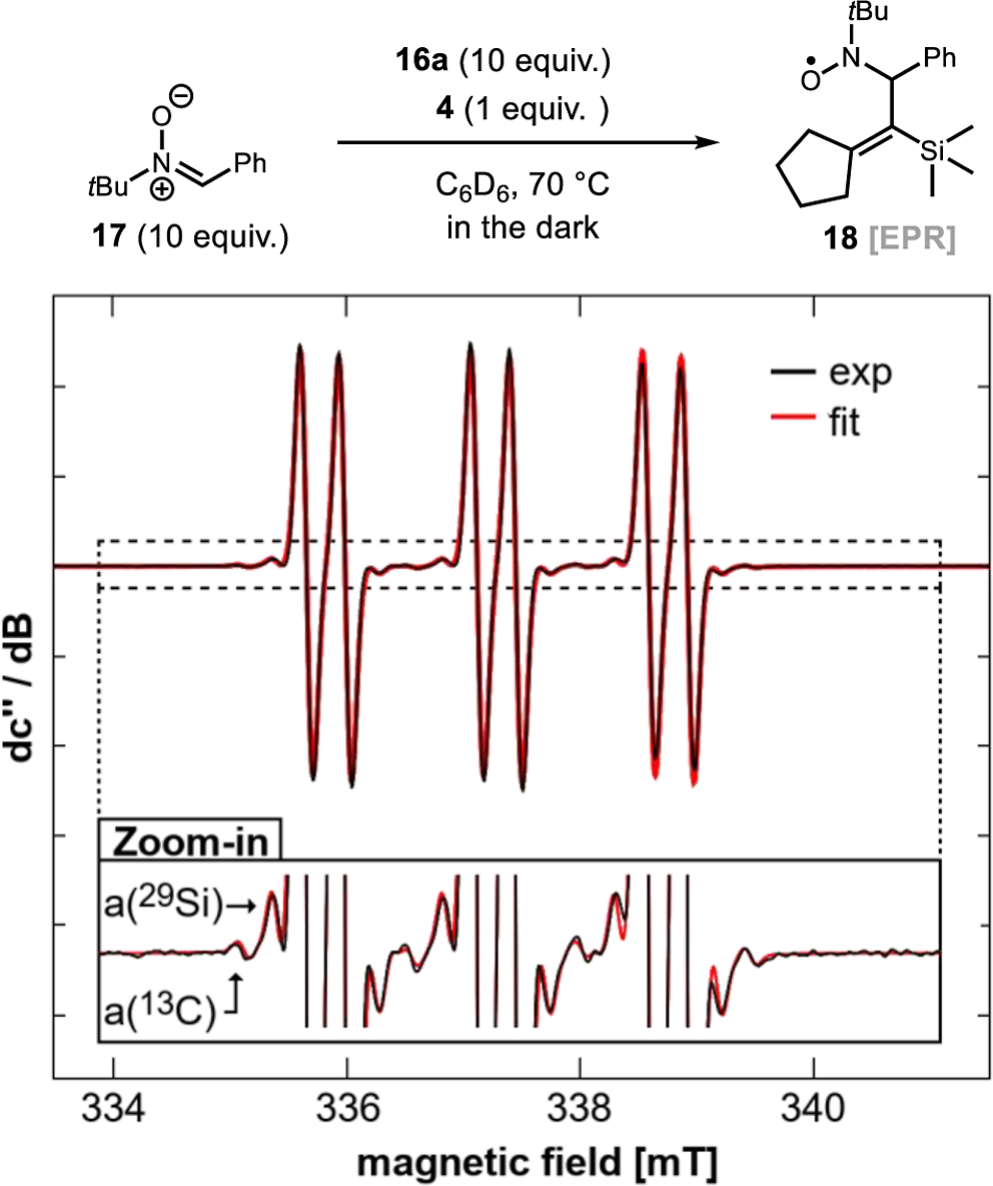
Radical trap experiment and EPR spectra of **18**. Experimental (black) and simulated (red) continuous-wave (CW) X-band EPR spectra of a solution containing 1 equiv BiMn­(CO)_5_(C_6_H_4_)_2_S (**4**) (*c* = 0.05 × 10^–5^ mol/L), 10 equiv substrate **16a**, and 10 equiv PBN **17** in benzene-*d*_6_ (0.5 mL). Spectrometer settings were as follows: microwave frequency = 9.466495 GHz, 0.02 mT modulation amplitude at 100 kHz, microwave power = 1 mW, number of accumulated scans = 1, and conversion time = 2 ms.

Bolstered by the promising performance of catalyst **4**, we investigated the preparative scope of the reaction. Overall, thermally induced ATRC exhibited mostly high yields and good functional group tolerance (Table 2).

**Table 2:**
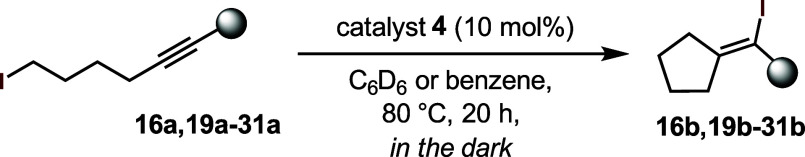

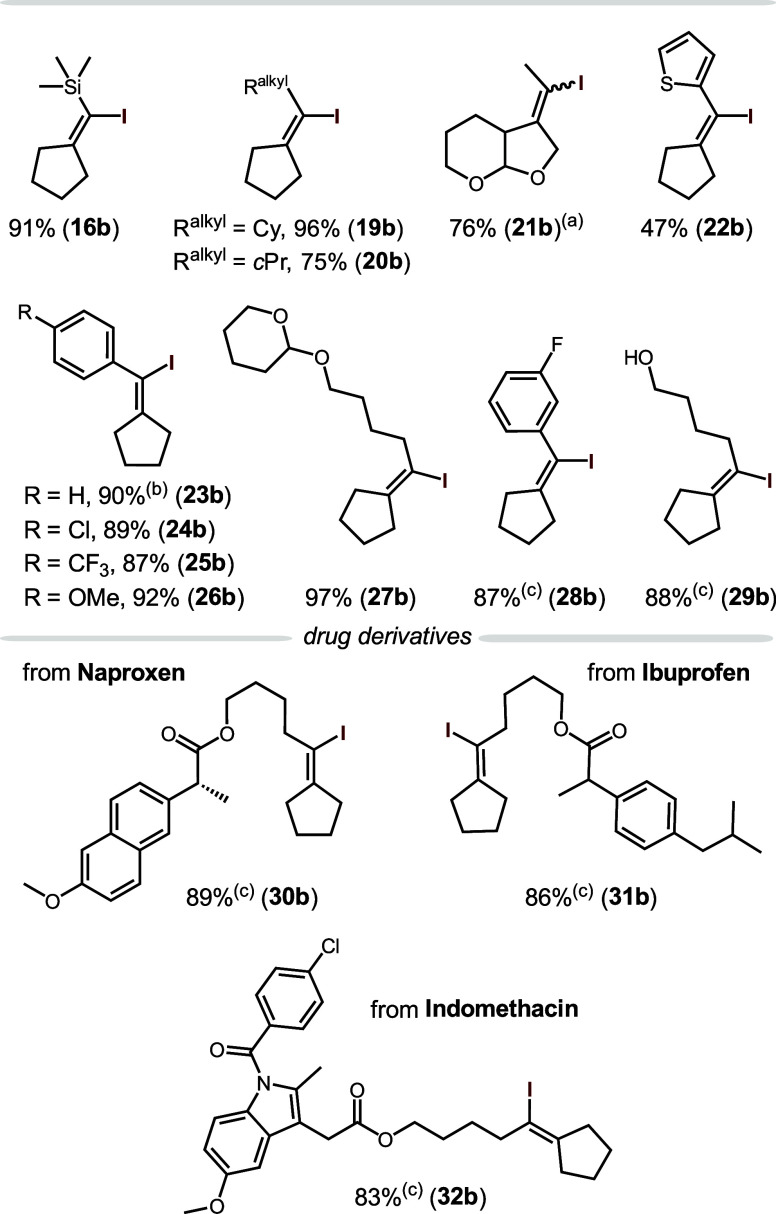
Thermally Induced Redox-Neutral Intramolecular C­(sp^2^)–C­(sp^3^) Coupling of Unactivated Alkyl Iodides and Alkynes Catalyzed by a Bi–Mn Complex^d^

a Performed on the 0.025 mmol scale.

b When performed on the 0.2 mmol scale, a yield of 85% was obtained (see text).

c Isolated yield.

d Yields determined by ^1^H NMR spectroscopy using 1,3,5-trimethoxybenzene as an internal standard. Reaction scale: substrate 0.05 mmol.

For instance, silyl ether (**16b**), thiophene (**22b**), or even free alcohol (**29b**) functional groups could perfectly be accommodated. A protected alcohol (**27b**) motif was also well-tolerated. Alkenyl iodides with cyclohexyl (**19b**) or cyclopropyl (**20b**) substituents were obtained in good yields. Notably, the cyclopropyl substituent did not undergo ring opening, in agreement with related protocols operating in the radical regime.^33^ Several aryl-substituted substrates bearing diverse electronic demands provided the targeted products (**23b**-**26b** and **28b**) without affecting the good performance of the catalyst. A secondary alkyl iodide also formed the desired cyclized product **21b** in good yield while at the same time demonstrating the feasibility of tolerating and forming cyclic ethers under standard conditions. Further substrates that gave the desired compounds in moderate yield are presented in the Supporting Information (including amine and cyano functional groups as well as intermolecular reactions). Next, we extended our reaction scope to test its compatibility in more structurally complex contexts, for which we prepared a series of drug derivative substrates (**30a**–**32a**) starting from Naproxen, Ibuprofen, and Indomethacin. To our delight, the catalytic reactions proceeded smoothly, readily furnishing the targeted products **30b**–**32b** in high yields. These examples demonstrate that ether, ester, and amide functionalities are well-tolerated. The results presented in Table 2 showcase the robustness of the ATRC reaction while operating under purely thermal conditions. As a proof-of-principle, the cyclo-isomerization of **23a** was conducted on the 0.2 mmol scale, which gave the desired compound **23b** in consistently high yields of 85%. A reaction mechanism involving equilibrium radical reactions and bismuth alkyl species as key intermediates is tentatively proposed based on chemical logic and experimental observations (Supporting Information).

## Conclusions

In summary, we have disclosed new bismuth-based catalysts that enable the unconventional thermally induced ATRC reaction of alkyl iodides and alkynes. Initial investigations by means of density functional theory (DFT) studies facilitated the identification of suitable Bi–Mn complexes. EPR studies show evidence of the radical character of the reaction and proof that the radical cyclization is fast compared with radical trapping. The preparative scope of the catalytic transformation with our methodology building upon purely thermal reaction initiation showcased high chemoselectivity and versatility while delivering good to excellent yields, even in more complex molecular environments. This is a very rare example of a methodology enabling the thermally initiated radical cyclo-isomerization between unactivated alkyl iodide and alkyne functional groups. This precious-metal-free, thermally driven strategy provides an innovative and tunable approach complementary to existing photochemical protocols.

## Methods

### Compound **4**

The bismuth halide **4**-**I** (130 mg, 0.25 mmol, 1.00 equiv) was dissolved in THF (2.5 mL). The solution was cooled to 0 °C, and a solution of [Na­(thf)_3_]­[Mn­(CO)_5_] (0.25 mmol, 108 mg, 1.00 equiv) in THF (2.5 mL) was added dropwise. The reaction mixture was warmed to ambient temperature overnight. The reaction mixture was filtered, and all volatiles were removed from the reaction mixture under reduced pressure. The residue was washed with pentane (2 × 5 mL) and dried in vacuo to give compound **4** (120 mg, 0.20 mmol, 82%) as a pale orange solid. The product was analyzed by ^1^H and ^13^C nuclear magnetic resonance (NMR) spectroscopy, IR spectroscopy, mass spectrometry, elemental analysis, and single-crystal X-ray diffraction (for details, see Supporting Information, including the synthesis of precursors and other bismuth compounds covered in this work).

### Catalytic Experiments

In a typical catalytic experiment, a J. Young-NMR tube was sequentially charged with substrate (0.05 mmol), benzene-*d*_6_ (0.5 mL), and the selected catalyst (5.0 μmol, 10 mol %). The NMR tube was covered in aluminum foil and placed in a heating block at the indicated temperature and for the indicated time. Upon optimal conversion of the starting material (as judged by ^1^H NMR spectroscopy), TMB (1,3,5-trimethoxybenzene) was added as an internal standard, and the yield was determined. Further details of analyses and analytical data are given in Supporting Information.

## Supplementary Material















## Data Availability

Crystallographic data (CIF, CCDC numbers: 2446115–2446120).

## Notes

Experimental procedures, computational and spectral data, and crystallographic details (PDF)
Crystallographic information file (CIF)
Crystallographic information file (CIF)
Crystallographic information file (CIF)
Crystallographic information file (CIF)
Crystallographic information file (CIF)
Crystallographic information file (CIF)

## Abbreviations

ATRC: atom transfer radical cyclization
ATRA: atom transfer radical addition
NMR: nuclear magnetic resonance
XRD: X-ray diffraction.
Equiv: equivalents
MeCN: acetonitrile
THF: tetrahydrofuran
DFT: density functional theory
EPR: electron paramagnetic resonance
