# Supplementary material for: Bismuth Radical Catalysis: Thermally Induced Intramolecular C(sp3)–C(sp) Cyclization of Unactivated Alkyl Iodides and Alkynes
Source: ACS Catal. 2025 Aug 13;15(17):14976–82. doi: 10.1021/acscatal.5c02812 (PMC12418314; doi:10.1021/acscatal.5c02812)

Supporting Information

Bismuth Radical Catalysis: Thermally-induced Intramolecular C(sp^3)-C(sp)-Cyclization of Unactivated Alkyl Iodides and Alkynes

Sebastián Martínez, Marius André Junghanns, Tobias Dunaj, and Crispin Lichtenberg*

Department of Chemistry, Philipps-University Marburg, 35032 Marburg, Germany.
Email: crispin.lichtenberg@chemie.uni-marburg.de

Table of Contents

General considerations 3
Preparation of starting materials 3
Preparation of ligands 10
Preparation of bismuth compounds 12
General Procedure for the preparation of triaryl-bismuth precursors. 12
General Procedure for the preparation of bismuth halide precursors. 12
General Procedure for the preparation of bismuth-manganese compounds 12
Compound **2-Ar**. 13
Compound **2-I**. 13
Compound **2**. 14
Compound **3-Ar**. 14
Compound **3-I**. 15
Compound **3**. 15
Compound **4-Ar**. 16
Compound **4-I**. 17
Compound **4**. 17
Single-crystal X-ray analysis 19
Compound **2-I**: [(κ^2-$C_{12}H_8SO_2$)BiI] 19
Compound **2**: [(κ^2-$C_{12}H_8SO_2$)Bi(Mn(CO)$_5$)]·(MeCN) 19
Compound **3-I**: [(κ^2-$C_{13}H_{10}$)BiI] 20
Compound **3**: [(κ^2-$C_{13}H_{10}$)Bi(Mn(CO)$_5$)] 21
Compound **4-I**: [(κ^2-$C_{12}H_8S$)BiI] 21
Compound **4**: [(κ^2-$C_{12}H_8S$)Bi(Mn(CO)$_5$)] 22
Catalytic reactions 23
General procedure 23
Exploratory studies 23
Catalyst loading (substrate **16a**, catalyst **4**) 24
Characterization of products 24
Additional substrates 28
Mechanistic considerations 29
EPR spectroscopic measurements 30
Computational screening of catalyst candidates 31
Computational details 31
References 34
Cartesian coordinates 36
IR spectra 51
Copies of NMR spectra 52

General considerations

All air and moisture-sensitive manipulations were carried out using standard Schlenk techniques or in a glovebox containing purified argon. Solvents were purified by distillation using the appropriate drying agents, degassed and stored over molecular sieves prior to use (3 Å for acetonitrile, 4 Å for other solvents used here). Deuterated solvents used for NMR spectroscopy were dried, degassed and stored over molecular sieves (3 Å or 4 Å, see above) under dry argon prior to use. All NMR spectra were acquired either on a Bruker Avance 300 spectrometer or on Bruker Avance I/III 500 spectrometer. All chemical shifts are reported in ppm. ^{1}H and ^{13}C chemical shifts are reported relative to $SiMe_4$ using the residual solvent peak of the solvent as a secondary standard. ^{19}F chemical shifts are reported relative to $CFCl_3$ as an external standard. Elemental analyses (C, H, N, S) were conducted on Vario Micro Cube instruments by Elementar Analysesysteme GmbH. HR-ESI mass spectra were acquired with an Orbitrap Q Exactive plus mass spectrometer (Thermo Fischer Scientific), with a resolution set to 140,000. HR-FD/FI/LIFDI mass spectra were acquired with an AccuTOF GCv 4G (JEOL) Time of Flight (TOF) mass spectrometer. An internal or external standard was used for drift time correction. The LIFDI ion source and FD emitters were purchased from Linden ChroMasSpec GmbH (Bremen, Germany). Single-crystals suitable for X-ray diffraction analysis were coated with perfluorinated polyether oil in a glovebox, transferred to a nylon loop and then transferred to the goniometer of a diffractometer (Bruker D8 Quest or Bruker D8 Venture or Stoe IPDS-2T or Stoe Stadivari) equipped with a molybdenum or copper X-ray tube (λ = 0.71073 Å or 1.54178 Å). The structures were solved using Olex2,[1] with XT structure solution program[2] and refined with the XL refinement package.[3]

Preparation of starting materials

6-iodohex-1-ene S1

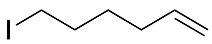

The compound was prepared starting from 6-bromo-1-hexene (1.00 g, 6.17 mmol, 1.00 equiv.) and NaI (2.77 g, 18.51 mmol, 3.00 equiv.), yielding the title product (1.04 g, 4.95 mmol, 80%) as a colorless oil. The NMR data match previously reported data for the title product.[4]

^{1}H NMR (300 MHz, $CDCl_3$) δ 5.84 – 5.57 (m, 1H), 5.06 – 4.76 (m, 2H), 3.09 (t, J = 7.0 Hz, 2H), 2.05 – 1.90 (m, 2H), 1.81 – 1.66 (m, 2H), 1.47 – 1.33 (m, 2H).

General procedures for the preparation of substrates

Procedure A

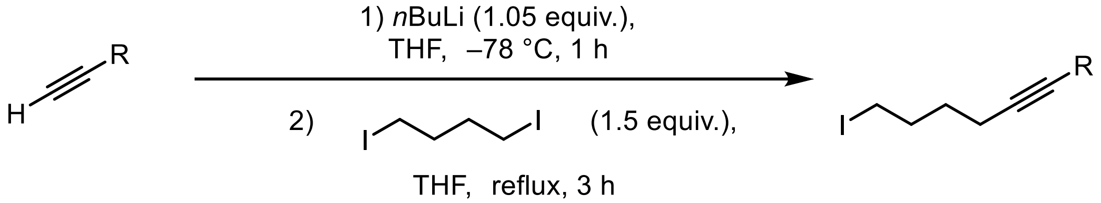

To a stirred solution of the terminal alkyne (2.00 mmol, 1.00 equiv.) in THF (10 mL) under inert gas atmosphere at –78 °C, *n*BuLi (0.84 mL, 2.10 mmol, 2.5 M, 1.05 equiv.) was added dropwise. The reaction mixture was stirred for 1 hour and then warmed up to room temperature. Then, a solution of 1,4-diiodobutane (930 mg, 3.00 mmol, 1.50 equiv.) in THF (5 mL) was added and the mixture was heated to reflux for 3 hours. Upon full consumption of the alkyne, the mixture was cooled down to room temperature, diluted with diethyl ether (50 mL), washed with water (2 × 10 mL) and brine (2 × 10 mL). The organic layer was dried over anhydrous $MgSO_4$, filtered, and concentrated under reduced pressure. The residue was purified by column chromatography (silica, hexane 100%), yielding the corresponding alkyl iodide.

Procedure B

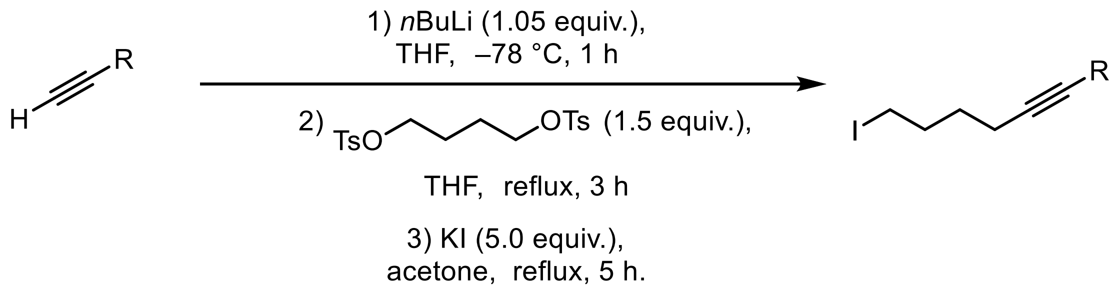

To a stirred solution of the terminal alkyne (2.00 mmol, 1.00 equiv.) in THF (10 mL) under inert gas atmosphere at –78 °C, *n*BuLi (0.84 mL, 2.10 mmol, 2.5 M, 1.05 equiv.) was added dropwise. The reaction mixture was stirred for 1 hour and then warmed up to room temperature. Then, a solution of butane-1,4-diyl bis(4-methylbenzenesulfonate) (1194 mg, 3.00 mmol, 1.50 equiv.) in THF (5 mL) was added and the mixture was heated to reflux for 3 hours. Upon full consumption of the alkyne, the mixture was cooled down to room temperature, diluted with diethyl ether (50 mL), washed with water (2 × 20 mL) and brine (1 × 20 mL). The organic layer was dried over anhydrous $MgSO_4$, filtered, and concentrated under reduced pressure. The residue was then dissolved in acetone (20 mL), KI (1.65 g, 10.0 mmol, 5.00 equiv.) was added and the mixture was heated to reflux for 5 hours. Then, water (50 mL) and diethyl ether (50 mL) were added. The aqueous phase was extracted with diethyl ether (2 × 50 mL), dried over anhydrous $MgSO_4$, filtered, and concentrated under reduced pressure. The residue was purified by column chromatography (silica, hexane 100%), yielding the corresponding alkyl iodide.

(6-iodohex-1-yn-1-yl)trimethylsilane 16a

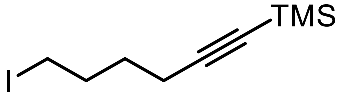

The compound was prepared following procedure **A**, starting from ethynyltrimethylsilane (196 mg, 2.00 mmol, 1.00 equiv.), yielding the title product (392 mg, 1.40 mmol, 70%) as a colorless oil. The NMR data match previously reported data for the title product.[5]

^{1}H NMR (300 MHz, $CDCl_3$) δ 3.44 (t, J = 6.6 Hz, 2H), 2.39 – 2.14 (m, 2H), 2.06 – 1.92 (m, 2H), 1.81 – 1.34 (m, 4H), 0.15 (s, 9H).

(6-iodohex-1-yn-1-yl)cyclohexane 19a

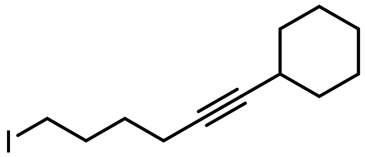

The compound was prepared following procedure **A**, starting from ethynylcyclohexane (216 mg, 2.00 mmol, 1.00 equiv.), yielding the title product (423 mg, 1.46 mmol, 73%) as a colorless oil. The NMR data match previously reported data for the title product.[6]

^{1}H NMR (300 MHz, CD_2Cl_2) δ 3.23 (t, *J* = 7.0 Hz, 2H), 2.46 – 2.21 (m, 1H), 2.26 – 2.05 (m, 2H), 1.97 – 1.89 (m, 2H), 1.78 – 1.51 (m, 6H), 1.40 – 1.25 (m, 4H).

(6-iodohex-1-yn-1-yl)cyclohexane 20a

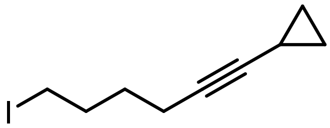

The compound was prepared following procedure **A**, starting from ethynylcyclopropane (132 mg, 2.00 mmol, 1.00 equiv.), yielding the title product (362 mg, 1.46 mmol, 73%) as a colorless oil. The NMR data match previously reported data for the title product.[6]

^{1}H NMR (300 MHz, CD_2Cl_2) δ 3.22 (t, J = 7.0 Hz, 2H), 2.22 – 2.05 (m, 2H), 1.95 – 1.81 (m, 2H), 1.66 – 1.53 (m, 2H), 0.77 – 0.63 (m, 2H), 0.63 – 0.44 (m, 2H).

2-butynyloxy-3-iodo-tetrahydropyran 21a

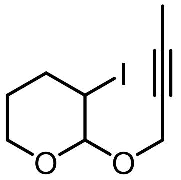

The compound was prepared according to the literature. The NMR data match previously reported data for the title product.[7]

^{1}H NMR (300 MHz, $CDCl_3$) δ 4.89 – 4.84 (d, J = 4.5 Hz, 1H), 4.36 – 4.16 (ddq, J = 9.2 Hz; 4.5 Hz; 2.3 Hz, 2H), 4.18 – 4.10 (dt, J = 7 Hz; 4.5 Hz, 1H), 4.00 – 3.88 (m, 1H), 3.66 – 3.55 (m, 1H), 2.42 – 2.27 (m, 1H), 2.06 – 1.93 (m, 1H), 1.92 – 1.77 (m, 1H), 1.87 (m, 3H, overlapping with other multiplet), 1.61 – 1.47 (m, 1H).

3-(6-iodohex-1-yn-1-yl)thiophene 22a

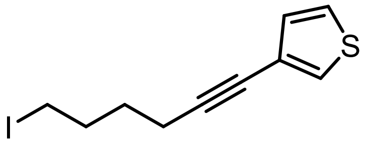

The compound was prepared following procedure **B**, starting from 3-ethynylthiophene (216 mg, 2.00 mmol, 1.00 equiv.), yielding the title product (267 mg, 0.92 mmol, 46%) as a colorless oil. The NMR data match previously reported data for the title product.[8]

^{1}H NMR (300 MHz, CD_2Cl_2) δ 7.37 – 7.34 (m, 1H), 7.28 – 7.25 (m, 1H), 7.08 – 7.05 (m, 1H), 3.26 (t, J = 6.9 Hz, 2H), 2.46 – 2.41 (m, 2H), 2.01 – 1.92 (m, 2H), 1.75 – 1.67 (m, 2H).

(6-iodohex-1-yn-1-yl)benzene 23a

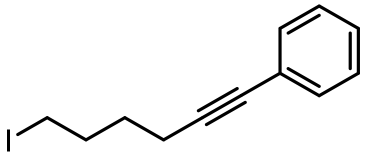

The compound was prepared following procedure **A**, starting from ethynylbenzene (204 mg, 2.00 mmol, 1.00 equiv.), yielding the title product (358 mg, 1.26 mmol, 63%) as a colorless oil. The NMR data match previously reported data for the title product.[9]

^{1}H NMR (300 MHz, C_6D_6) δ 7.52 – 7.40 (m, 2H), 7.07 – 6.89 (m, 3H), 2.65 (t, J = 6.9 Hz, 2H), 2.03 (t, J = 7.0 Hz, 2H), 1.60 – 1.48 (m, 2H), 1.34 – 1.27 (m, 2H).

1-chloro-4-(6-iodohex-1-yn-1-yl)benzene 24a

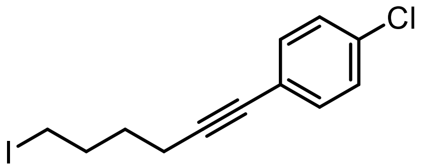

The compound was prepared following procedure **B**, starting from 1-chloro-4-ethynylbenzene (272 mg, 2.00 mmol, 1.00 equiv.), yielding the title product (343 mg, 1.08 mmol, 54%) as a colorless oil. The NMR data match previously reported data for the title product.[10]

^{1}H NMR (300 MHz, CD_2Cl_2) δ 7.41 – 7.16 (m, 4H), 3.26 (t, J = 6.9 Hz, 2H), 2.44 (t, J = 7.0 Hz, 2H), 2.05 – 1.94 (m, 2H), 1.77 – 1.63 (m, 2H).

1-(6-iodohex-1-yn-1-yl)-4-(trifluoromethyl)benzene 25a

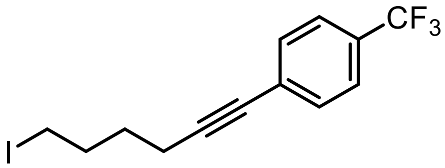

The compound was prepared following procedure **B**, starting from 1-ethynyl-4-(trifluoromethyl)benzene (340 mg, 2.00 mmol, 1.00 equiv.), yielding the title product (359 mg, 1.02 mmol, 51%) as a colorless oil. The NMR data match previously reported data for the title product.[8]

^{1}H NMR (300 MHz, CD_2Cl_2) δ 7.53 (d, J = 8.1 Hz, 4H), 3.27 (t, J = 6.9 Hz, 2H), 2.48 (t, J = 7.0 Hz, 2H), 2.06 – 1.95 (m, 2H), 1.78 – 1.68 (m, 2H).

1-(6-iodohex-1-yn-1-yl)-4-methoxybenzene 26a

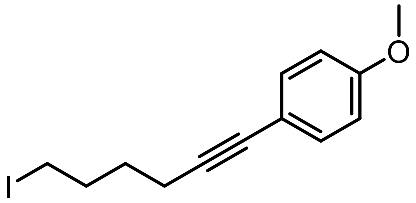

The compound was prepared following procedure **B**, starting from 1-ethynyl-4-methoxybenzene (264 mg, 2.00 mmol, 1.00 equiv.), yielding the title product (351 mg, 1.12 mmol, 56%) as a colorless oil. The NMR data match previously reported data for the title product.[8]

^{1}H NMR (300 MHz, CD_2Cl_2) δ 7.38 – 7.24 (m, 2H), 6.85 – 6.77 (m, 2H), 3.79 (s, 3H), 3.27 (t, J = 6.9 Hz, 2H), 2.43 (t, J = 7.0 Hz, 2H), 2.05 – 1.94 (m, 2H), 1.75 – 1.63 (m, 2H).

2-((10-iododec-5-yn-1-yl)oxy)tetrahydro-2H-pyran 27a

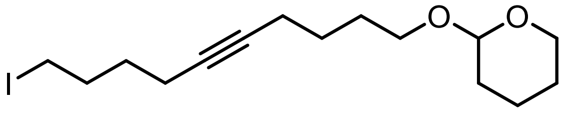

The compound was prepared following procedure **A**, starting from 2-(hex-5-yn-1-yloxy)tetrahydro-2H-pyran (728 mg, 4.00 mmol, 1.00 equiv.), yielding the title product (858 mg, 2.36 mmol, 59%) as a colorless oil. The NMR data match previously reported data for the title product.[6]

^{1}H NMR (300 MHz, CD_2Cl_2) δ 4.65 – 4.45 (m, 1H), 3.89 – 3.64 (m, 2H), 3.60 – 3.16 (m, 4H), 2.44 – 2.12 (m, 4H), 1.98 – 1.48 (m, 14H).

1-fluoro-3-(6-iodohex-1-yn-1-yl)benzene 28a

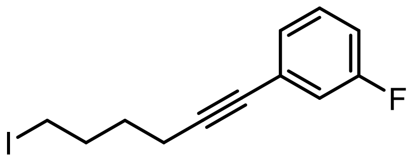

The compound was prepared following procedure **B**, starting from 1-ethynyl-3-fluorobenzene (240 mg, 2.00 mmol, 1.00 equiv.), yielding the title product (283 mg, 0.94 mmol, 47%) as a colorless oil. The NMR data match previously reported data for the title product.[11]

^{1}H NMR (300 MHz, CD_2Cl_2) δ 7.33 – 7.22 (m, 1H), 7.21 – 7.14 (m, 1H), 7.13 – 6.92 (m, 2H), 3.27 (t, J = 6.9 Hz, 2H), 2.45 (t, J = 7.0 Hz, 2H), 2.04 – 1.93 (m, 2H), 1.76 – 1.66 (m, 2H).

10-iododec-5-yn-1-ol 29a

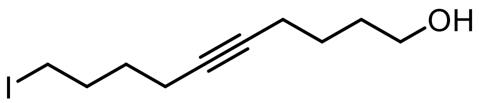

To a stirred solution of 2-((10-iododec-5-yn-1-yl)oxy)tetrahydro-2H-pyran **27a** (544 mg, 1.5 mmol, 1.00 equiv.) in methanol (15 mL), was added TsOH·H_2O (0.1 equiv.) at room temperature. The reaction was allowed to stir overnight and then the solvent was removed under reduced pressure. The reaction mixture was diluted with ethyl acetate (50 mL), washed with a saturated aqueous solution of $NaHCO_3$ (2 × 20 mL) and brine (2 × 20 mL). The organic layer was dried over anhydrous $MgSO_4$, filtered, concentrated under vacuum and the residue was purified by column chromatography (silica, 10% to 100% ethyl acetate in hexane), yielding the title product (374 mg, 1.33 mmol, 89%) as a colorless oil. The NMR data match previously reported data for the title product.[6]

^{1}H NMR (300 MHz, C_6D_6) δ 3.44 – 3.31 (m, 2H), 2.70 (t, J = 7.0 Hz, 2H), 2.15 – 2.00 (m, 2H), 1.96 – 1.88 (m, 2H), 1.64 – 1.53 (m, 2H), 1.52 – 1.41 (m, 5H), 1.33 – 1.23 (m, 2H).

10-iododec-5-yn-1-yl (S)-2-(6-methoxynaphthalen-2-yl)propanoate 30a

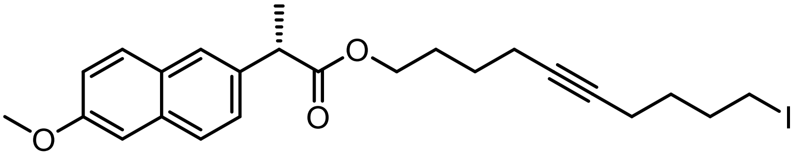

1) Naproxen extraction. Naproxen tablets (10 × 250 mg) were crushed to powder using a mortar and pestle. The resulting powder was mixed with 100 mL of ethyl acetate and 3M HCl (50 mL), and stirred at room temperature for 30 min. The organic layer was separated, and the aqueous layer was extracted with ethyl acetate (3 × 50 mL). The combined organic layers were concentrated under reduced pressure, yielding pure neutral Naproxen (2.22g, 89% yield).

2) Naproxen acyl-chloride *preparation*. Naproxen acyl-chloride ((S)-2-(6-methoxynaphthalen-2-yl)propanoyl chloride) was prepared by refluxing a solution of Naproxen (690 mg, 3.00 mmol, 1.00 equiv.) and thionyl chloride (0.25 mL, 407 mg, 3.45 mmol, 1.15 equiv.) in benzene (30 mL) in the presence of catalytic amounts of pyridine (2.2 mg, 0.03 mmol, 5.0 mol%) for 5 hours. The resulting mixture was then concentrated under reduced pressure, and the crude acyl-chloride product was used without further purification.

3) The title compound was prepared by reaction of a mixture of freshly prepared Naproxen acyl-chloride (62 mg, 0.25 mmol, 1.00 equiv.) and the alcohol 10-iododec-5-yn-1-ol **29a** (70 mg, 0.25 mmol, 1.00 equiv.) in dry DCM (5 mL), after 20 hours of stirring at room temperature. The resulting mixture was concentrated under reduced pressure and the residue was purified by column chromatography (silica, 20% ethyl acetate in hexane) yielding the title product (110 mg, 0.22 mmol, 89% yield) as a colorless oil.

^{1}H NMR (300 MHz, $CDCl_3$) δ 7.77 – 7.62 (m, 3H), 7.47 – 7.35 (m, 1H), 7.18 – 7.08 (m, 2H), 4.17 – 4.00 (m, 2H), 3.92 (s, 3H), 3.89 – 3.78 (m, 1H), 3.18 (t, *J* = 7.0 Hz, 2H), 2.23 – 2.03 (m, 4H), 1.97 – 1.81 (m, 2H), 1.74 – 1.62 (m, 2H), 1.62 – 1.39 (m, 7H).

^{13}C NMR (75 MHz, $CDCl_3$) δ 174.83, 157.78, 135.93, 133.82, 129.41, 129.09, 127.24, 126.41, 126.06, 119.09, 105.76, 80.31, 64.52, 55.47, 45.66, 32.62, 29.81, 27.87, 25.49, 18.68, 18.46, 17.83, 6.44.

HRMS (ESI): calc'd for ($C_{24}H_{29}IO_3Na$) $[M+Na]^+$ 515.1054, found 515.1063.

10-iododec-5-yn-1-yl 2-(4-isobutylphenyl)propanoate 31a

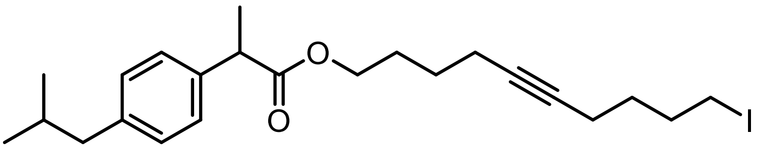

1) Ibuprofen extraction. Ibuprofen tablets (10 x 600 mg) were crushed to powder using a mortar and pestle. The resulting powder was mixed with hexane (500 mL) and stirred at room temperature for 30 min. The undissolved solid was filtered off and the resulting solution was concentrated under reduced pressure, yielding pure Ibuprofen (5.90 g, 98% yield).

2) Ibuprofen acyl-chloride *preparation*. Ibuprofen acyl-chloride (2-(4-isobutylphenyl)propanoyl chloride) was prepared by refluxing a solution of Ibuprofen (618 mg, 3.00 mmol, 1.00 equiv.) and thionyl chloride (0.25 mL, 407 mg, 3.45 mmol, 1.15 equiv.) in benzene (30 mL) in the presence of catalytic amounts of pyridine (2.2 mg, 0.03 mmol, 5.0 mol%) for 5 hours. The resulting mixture was then concentrated under reduced pressure, and the crude acyl-chloride product was used without further purification.

3) The title compound was prepared by reaction of a mixture of freshly prepared Ibuprofen acyl-chloride (112 mg, 0.50 mmol, 1.00 equiv.) and the alcohol 10-iododec-5-yn-1-ol **29a** (140 mg, 0.50 mmol, 1.00 equiv.) in dry DCM (10 mL), after 20 hours of stirring at room temperature. The resulting mixture was concentrated under reduced pressure and the residue was purified by column chromatography (silica, 10% ethyl acetate in hexane) yielding the title product (211 mg, 0.45 mmol, 90% yield) as a colorless oil.

^{1}H NMR (300 MHz, $CDCl_3$) δ 7.20 (d, J = 8.2 Hz, 2H), 7.09 (d, J = 8.2 Hz, 2H), 4.08 (t, J = 6.5 Hz, 2H), 3.68 (q, J = 7.2 Hz, 1H), 3.20 (t, J = 6.9 Hz, 2H), 2.45 (d, J = 7.2 Hz, 2H), 2.25 –

2.06 (m, 4H), 1.98 – 1.78 (m, 3H), 1.73 – 1.54 (m, 4H), 1.50 – 1.39 (m, 5H), 0.89 (d, J = 6.6 Hz, 6H).

^{13}C NMR (75 MHz, CDCl3) δ 174.87, 140.59, 137.99, 129.41, 127.28, 80.33, 64.36, 45.32, 45.18, 32.62, 30.31, 29.82, 27.85, 25.45, 22.51, 18.60, 18.44, 17.84, 6.39.

HRMS (ESI): calc'd for ($C_{23}H_{33}IO_2Na$) $[M+Na]^+$ 491.1417, found 491.1423.

10-iododec-5-yn-1-yl 2-(1-(4-chlorobenzoyl)-5-methoxy-2-methyl-1H-indol-3-yl)acetate 32a

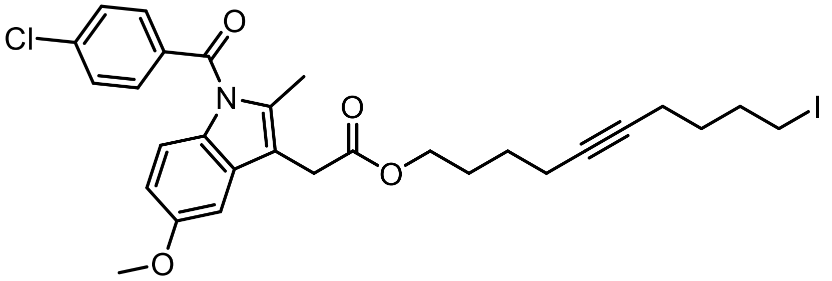

1) Indomethacin acyl-chloride preparation. Indomethacin acyl-chloride (2-(1-(4-chlorobenzoyl)-5-methoxy-2-methyl-1H-indol-3-yl)acetyl chloride) was prepared by refluxing a solution of Indomethacin (1.07 g, 3.00 mmol, 1.00 equiv.) and thionyl chloride (0.25 mL, 407 mg, 3.45 mmol, 1.15 equiv.) in benzene (30 mL) in the presence of catalytic amounts of pyridine (2.2 mg, 0.03 mmol, 5.0 mol%) for 5 hours. The resulting mixture was then concentrated under reduced pressure, and the crude acyl-chloride product was used without further purification.

2) The title compound was prepared by reaction of a mixture of freshly prepared Indomethacin acyl-chloride (94 mg, 0.25 mmol, 1.00 equiv.) and the alcohol 10-iododec-5-yn-1-ol **29a** (70 mg, 0.25 mmol, 1.00 equiv.) in dry DCM (5 mL), after 20 hours of stirring at room temperature. The resulting mixture was concentrated under reduced pressure and the residue was purified by column chromatography (silica, 20% ethyl acetate in hexane) yielding the title product (120 mg, 0.19 mmol, 77% yield) as a colorless oil.

^{1}H NMR (300 MHz, $CDCl_3$) δ 7.66 (d, *J* = 8.5 Hz, 2H), 7.47 (d, *J* = 8.5 Hz, 2H), 6.96 (d, *J* = 2.5 Hz, 1H), 6.87 (d, *J* = 9.0 Hz, 1H), 6.67 (dd, *J* = 9.1, 2.5 Hz, 1H), 4.12 (t, *J* = 6.5 Hz, 2H), 3.84 (s, 3H), 3.66 (s, 2H), 3.19 (t, *J* = 6.9 Hz, 2H), 2.39 (s, 3H), 2.28 – 2.03 (m, 4H), 2.03 – 1.82 (m, 2H), 1.80 – 1.65 (m, 2H), 1.64 – 1.41 (m, 4H).

^{13}C NMR (75 MHz, $CDCl_3$) δ 170.88, 168.29, 156.08, 139.27, 135.91, 133.96, 131.19, 130.84, 130.67, 129.13, 114.96, 112.68, 111.68, 101.33, 80.06, 79.85, 64.67, 55.73, 32.52, 30.43, 29.68, 27.80, 25.45, 18.37, 17.71, 13.37, 6.26.

HRMS (ESI): calc'd for ($C_{29}H_{32}ClINO_4$) $[M+H]^+$ 620.1059, found 620.1063.

Preparation of ligands

Ligand **L1** was prepared according to a literature procedure. [12] Ligand **L2** was prepared according to a literature procedure.[13,14] Ligand **L3** was purchased from Thermo Scientific®.

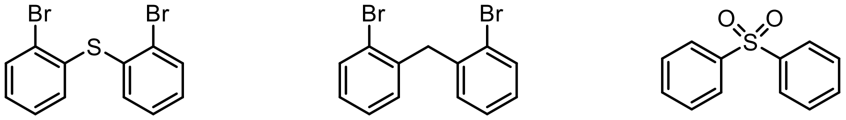

Scheme S1. Ligands utilized in this work.

Ligand L1

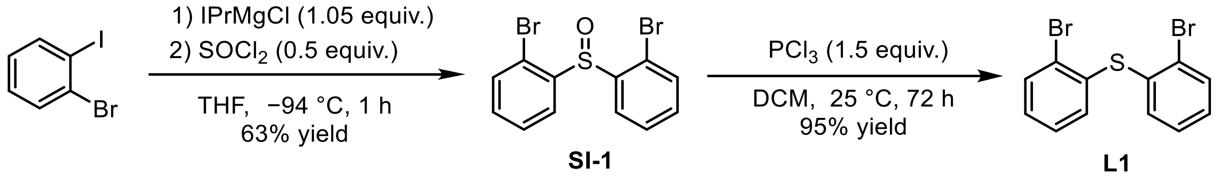

Bis(2,2'-bromophenyl)sulfoxide **SI-1**:

A solution of *i*PrMgCl (2.0 M in THF, 49 mL, 98.5 mmol, 1.05 equiv.) was added dropwise to a solution of 1-iodo-2-bromobenzene (12 mL, 93.5 mmol, 1.00 equiv.) in THF (100 ml) at – 94 °C over the course of 1 h 30 min. Then, $SOCl_2$ (3.4 mL, 47 mmol, 0.50 equiv.) was added and the reaction was stirred for 1 h 30 min at –94 °C. The reaction was quenched by addition of a saturated aqueous solution of NH_4Cl (50 mL), the organic layer was collected and the aqueous layer was extracted with Et_2O (3 × 40 mL). The combined organic layers were dried with $MgSO_4$ and concentrated under reduced pressure. The crude product was poured into hexane and filtrated yielding bis(2,2'-bromophenyl)sulfoxide **SI-1** (10.54 g, 29.44 mmol, 63%) as a pale yellowish solid, which was used in the next step without further purification. ^{1}H NMR data is consistent with the one reported in the literature.[15]

^{1}H NMR (300 MHz, $CDCl_3$) δ 7.68 (dd, *J* = 7.8, 1.7 Hz, 2H), 7.60 (dd, *J* = 7.9, 1.3 Hz, 2H), 7.54 – 7.45 (m, 2H), 7.41 – 7.32 (m, 2H).

Bis(2,2'-bromophenyl)sulfide **L1**:

To solution of bis(2,2'-bromophenyl)sulfoxide **SI-1** (10.54 g, 29.44 mmol, 1.00 equiv.) in dry DCM (100 mL) under inert atmosphere at room temperature was added PCl_3 (d = 1.574 g/mL, 3.86 mL, 44.2 mmol, 1.50 equiv.). After 72 – 96 h at room temperature and full consumption of the starting material (monitored by TLC), the reaction mixture was diluted with DCM (100 mL) and quenched by addition of water (100 mL) at 0 °C. The organic layer was collected and the aqueous layer was extracted with DCM (2 × 50 mL). The combined organic extracts were filtered over a silica gel plug followed by subsequent evaporation to dryness. The crude product was purified by column chromatography (silica, 100 % hexane) yielding bis(2,2'-bromophenyl)sulfide **L1** as colorless solid (9.84 g, 28.0 mmol, 95% yield). ^{1}H NMR data in $CDCl_3$ is consistent with the one reported in the literature. [12] We include also ^{1}H NMR data in CD_2Cl_2.

^{1}H NMR (300 MHz, CD_2Cl_2) δ 7.66 (dd, *J* = 7.8, 1.5 Hz, 2H), 7.32 – 7.23 (m, 2H), 7.23 – 7.07 (m, 4H).

Ligand L2

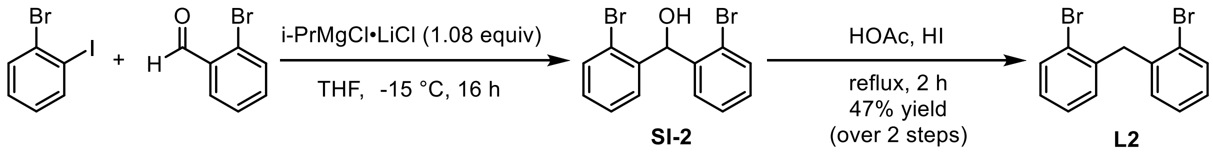

bis-(2-bromophenyl)-methanol **SI-2**:

To a stirring solution of 2-bromo-iodobenzene (14.0 g, 50.0 mmol, 1.00 equiv.) in THF (250 mL) was added *i*PrMgCl (2.0 M in THF, 27.0 mL, 54.0 mmol, 1.08 equiv.) at −15 °C. After 4 hours of reaction time, the exchange was complete, as judged by GCMS through the detection of bromobenzene in quenched aliquots. The reaction mixture was then cooled to −78 °C and 2-bromobenzaldehyde was added dropwise with a syringe. The reaction was allowed to warm slowly to room temperature overnight. Then, the reaction mixture was diluted with diethyl ether and quenched with HCl 6M (aq.) until the aqueous layer was slightly acidic (litmus red colored) at which time an additional portion of water was added (100 mL). The phases were separated and the aqueous layer was extracted with diethyl ether (3 × 100 mL). The combined organics extracts were washed with brine, dried with $MgSO_4$ and concentrated in vacuo. The crude material was purified by column chromatography (silica, 5% ethyl acetate in hexane), yielding bis-(2-bromophenyl)-methanol **SI-2** (15.4 g, 45.0 mmol, 90%) as a colorless solid. ^{1}H NMR data is consistent with the one reported in the literature. [13,14]

^{1}H NMR (300 MHz, $CDCl_3$) δ 7.58 (d, *J* = 7.9 Hz, 2H), 7.39 – 7.28 (m, 4H), 7.23 – 7.15 (m, 2H), 6.41 (s, 1H), 2.05 (s, 1H).

Bis-(2-bromophenyl)-methane **L2**:

To a solution of bis(2-bromophenyl)methanol **SI-2** (15.4 g, 45.0 mmol, 1.00 equiv.) in acetic acid (300 mL) was added an aqueous solution of HI 55% (41.0 g, 180 mmol, 4.00 equiv.). The reaction mixture was heated under reflux for 2 hours, upon which full conversion of starting the material was observed (TLC). The reaction mixture was then stirred at room temperature for 18 hours and quenched by addition of a saturated solution of sodium sulfite until no further color change from dark to yellow was observed. Then the mixture was diluted with water (1 L) and the aqueous phase was extracted with diethyl ether (5 × 200 mL). The combined organic extracts were washed with NaOH 1M (aq.) until the aqueous phase was fully neutralized (litmus blue colored). The organic extracts were separated and dried over $MgSO_4$ and concentrated in vacuo. The crude product was purified by column chromatography (silica, 100% hexane) yielding Bis-(2-bromophenyl)-methane **L2** (7.58 g, 23.4 mmol, 52%) as a colorless oil. NMR data is consistent with the one reported in the literature.[13]

^{1}H NMR (300 MHz, $CDCl_3$) δ 7.61 (d, *J* = 7.9 Hz, 2H), 7.25 – 7.17 (m, 2H), 7.16 – 7.07 (m, 2H), 7.05 – 6.93 (m, 2H), 4.21 (s, 2H).

Preparation of bismuth compounds

General Procedure for the preparation of triaryl-bismuth precursors.

Under inert atmosphere, the appropriate ligand (2.00 mmol, 1.00 equiv.) was dissolved in anhydrous THF (10 mL) and cooled to –78 °C. Then *n*BuLi 2.5M in hexanes (4.1 mmol, 2.05 equiv.) was added dropwise and the solution was stirred for 1 h. The arylbismuthdibromide (2.00 mmol, 1 equiv.) was dissolved in THF (10 mL) and diethyl ether (8 mL) and slowly added to the lithiated ligand solution at –78 °C. The resulting solution was allowed to warm up to room temperature overnight, followed by the addition of a saturated NaCl (aq.) solution (20 mL) and extraction with EtOAc (2 × 50 mL) and DCM (2 × 50 mL). The organic phase was dried over $MgSO_4$ and filtered over silica with EtOAc as the eluent. The filtrated organic phase was concentrated in vacuo over silica, and the residue was subjected to column chromatography (silica, 0 - 5% DCM in hexane) yielding the corresponding product.

General Procedure for the preparation of bismuth halide precursors.

The appropriate triaryl-bismuth compound (1.00 mmol, 1.00 equiv.) was dissolved in DCM (20 mL) and then a solution of iodine (1.00 mmol, 1.00 equiv.) in DCM (40 mL) was added portion-wise. The mixture was stirred for 30 min and then filtered over celite. The filtrated solution was concentrated in vacuo over silica, and the residue was subjected to column chromatography (silica, 0 - 15% DCM in hexane) yielding the corresponding product.

General Procedure for the preparation of bismuth-manganese compounds

The appropriate bismuth halide complex (0.25 mmol, 1.00 equiv.) was dissolved in THF (2.5 mL) and cooled to 0 °C. A solution of $[Na(thf)_3][Mn(CO)_5]$ (0.25 mmol, 108 mg, 1.00 equiv.) in THF (2.5 mL) was added dropwise to the bismuth halide solution. The reaction mixture was stirred overnight allowing it to warm up to room temperature. The mixture was then filtered, concentrated in vacuo and the crude product was washed with pentane (2 × 5 mL) and dried in vacuo, yielding the corresponding product.

$[Na(thf)_3][Mn(CO)_5]$[16]

$[Na(thf)_3][Mn(CO)_5]$ was prepared in a mercury-free approach.

A solution of Mn_2CO_{10} (1.17 g, 3.0 mmol) in THF (40 mL) was added to Na/NaCl (5 wt%, 4.7 g, 10 mmol, 3.3 equiv.). The resulting mixture was stirred overnight at room temperature. The suspension was then filtered, and the residue was extracted with THF (10 mL). The solvent was removed from the combined filtrates under reduced pressure, yielding the product as a greyish-green solid. The product (1.276 g, 2.94 mmol, 98%) was then used without further purification. Analytical data was in agreement with the literature.[16]

Compound 2-Ar.

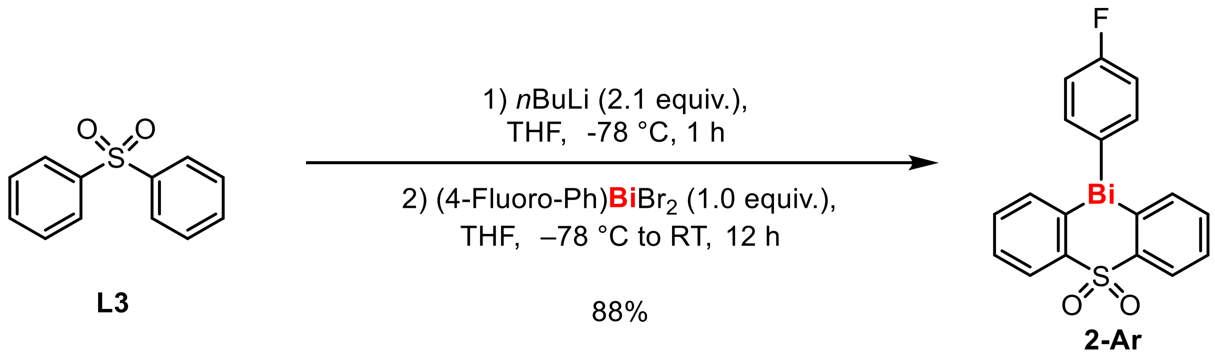

The compound was prepared according to the general procedure for the preparation of triaryl-bismuth precursors, starting from ligand **L3** (436 mg, 2.00 mmol, 1.00 equiv.) and (4-fluoro-phenyl) bismuthdibromide (923 mg, 2.00 mmol, 1.00 equiv.), yielding the title product (915 mg, 1.76 mmol, 88%) as a yellowish solid. NMR data is consistent with those reported in the literature.[17]

^{1}H NMR (300 MHz, $CDCl_3$) δ 8.38 (d, J = 7.5 Hz, 2H, CH_{arom}), 7.84 (d, J = 6.9 Hz, 2H, CH_{arom}), 7.78 – 7.64 (m, 2H, CH_{arom}), 7.48 – 7.30 (m, 4H, CH_{arom}), 7.13 – 6.99 (m, 2H, CH_{arom}).

Compound 2-I.

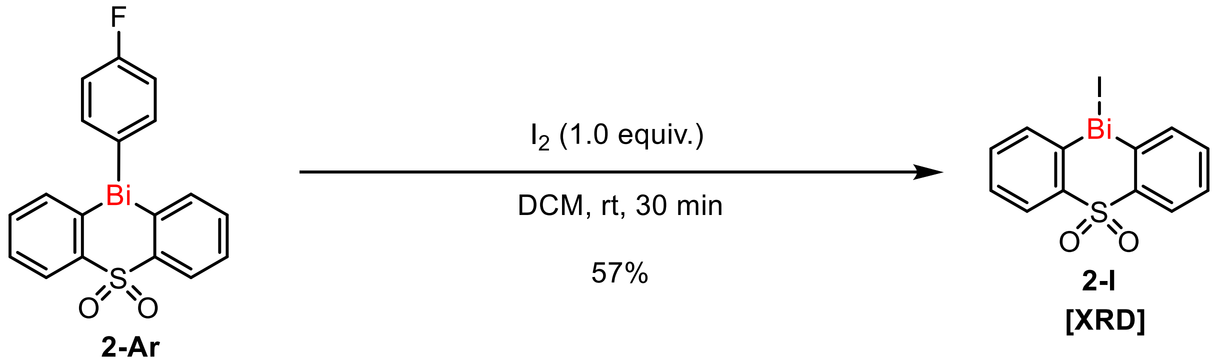

The compound was prepared according to the general procedure for the preparation of bismuth halide precursors, starting from compound **2-Ar** (520 mg, 1.00 mmol, 1.00 equiv.) and I_2 (254 mg, 1.00 mmol, 1.00 equiv.), yielding the title product (314 mg, 0.57 mmol, 57%) as a green-yellowish solid. Crystals suitable for SC-XRD were obtained by slow evaporation of a DCM solution of **2-I**. NMR data is consistent with those reported in the literature.[18,17]

^{1}H NMR (300 MHz, $CDCl_3$) δ 9.22 (d, *J* = 7.3 Hz, 2H, CH_{arom}), 8.30 (d, *J* = 7.7 Hz, 2H, CH_{arom}), 7.67 – 7.58 (m, 2H, CH_{arom}), 7.53 – 7.45 (m, 2H, CH_{arom}).

^{1}H NMR (300 MHz, DMSO) δ 9.25 (d, *J* = 7.3 Hz, 1H, CH_{arom}), 8.25 (d, *J* = 7.6 Hz, 1H, CH_{arom}), 7.77 – 7.66 (m, 1H, CH_{arom}), 7.59 – 7.48 (m, 1H, CH_{arom}).

Compound 2.

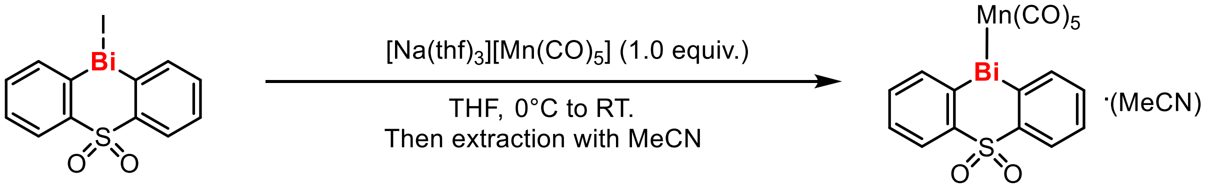

2-I → **2** **[XRD]**

The compound was prepared according to the general procedure for the preparation of bismuth-manganese compounds, starting from bismuth halide **2-I** (138 mg, 0.25 mmol, 1.00 equiv.) and $[Na(thf)_3][Mn(CO)_5]$ (0.25 mmol, 108 mg, 1.00 equiv.), followed by extraction with MeCN (2 × 5 mL), yielding the title product (92 mg, 0.14 mmol, 56%) as a dark brown solid. Crystals suitable for SC-XRD were obtained by slow evaporation of a MeCN solution of **2**.

^{1}H NMR (300 MHz, CD_3CN) δ 8.40 (dd, *J* = 7.2, 1.4 Hz, 1H, CH_{arom}), 8.32 – 8.18 (m, 1H, CH_{arom}), 7.39 – 7.23 (m, 2H, CH_{arom}), 1.94 – 1.88 (m, 3H, CH_3).

^{13}C NMR (75 MHz, CD_3CN) δ 141.91 (C_2CBi_{arom}), 141.64 (C_2CS_{arom}), 132.62 (C_2CH_{arom}), 132.52 (C_2CH_{arom}), 128.17 (C_2CH_{arom}), 126.50 (C_2CH_{arom}).

Note: C atoms of CO could not be observed in the ^{13}C NMR spectra (usually a weak broad signal around 219 ppm[14]). Signals of MeCN molecule in ^{13}C NMR are overlapped with the solvent signal (CD_3CN).

HRMS-LIFDI (positive mode) calc'd for $C_{17}H_8BiMnO_7S$ $[M]^+$: 619.9175, found: 619.9180.

IR: CO stretching modes: = 1942, 2010, 2037 cm^{-1}

Compound 3-Ar.

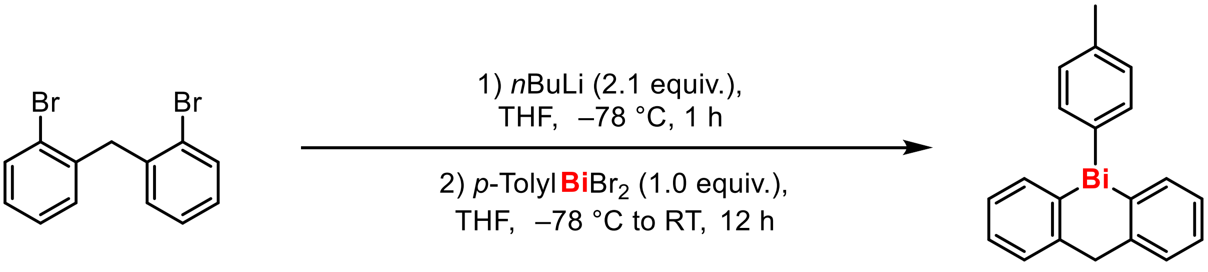

L2 → **3-Ar** (73%)

The compound was prepared according to the general procedure for the preparation of triaryl-bismuth precursors, starting from ligand **L2** (648 mg, 2.00 mmol, 1.00 equiv.) and *p*-tolylbismuthdibromide (916 mg, 2.00 mmol, 1.00 equiv.), yielding the title product (680 mg, 1.46 mmol, 73%) as a yellowish oil.

^{1}H NMR (300 MHz, CD_2Cl_2) δ 7.81 (dd, *J* = 7.0, 1.6 Hz, 2H, CH_{arom}), 7.64 – 7.57 (m, 4H, CH_{arom}), 7.25 – 7.09 (m, 6H, CH_{arom}), 4.04 – 3.80 (m, 2H, CH_2), 2.28 (s, 3H, CH_3).

$^{13}C\{^1H\}$ NMR (75 MHz, CD_2Cl_2) δ 144.52 (C_2CCH_{2arom}), 137.93 (C_2CCH_{3arom}), 137.51 (C_2CCH_{arom}), 137.27 (C_2CCH_{arom}), 131.15 (C_2CCH_{arom}), 131.03 (C_2CCH_{arom}), 128.75 (C_2CCH_{arom}), 128.35 (C_2CCH_{arom}), 127.49 (C_2CCH_{arom}), 21.10 (CH_3).

Note: C–Bi carbon atoms are not observable in the ^{13}C NMR spectra (due to line broadening effects arising from ^{209}Bi). Signals of CH_2 carbon in ^{13}C NMR are overlapped with the solvent signal (CD_2Cl_2).

HRMS (ESI): calc'd for $C_{20}H_{17}Bi$ M^+: 466.1134, found: 466.1121.

Compound 3-I.

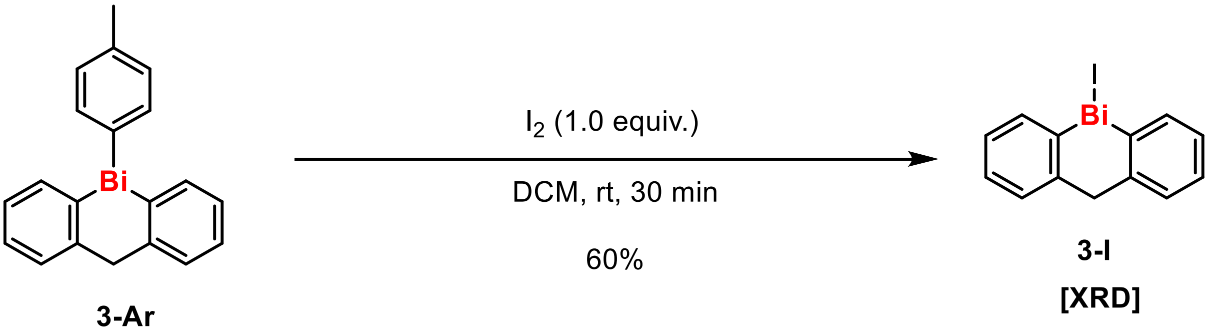

The compound was prepared according to the general procedure for the preparation of bismuth halide precursors, starting from compound **3-Ar** (466 mg, 1.00 mmol, 1.00 equiv.) and I_2 (254 mg, 1.00 mmol, 1.00 equiv.), yielding the title product (301 mg, 0.60 mmol, 60%) as a yellowish-orange solid. Crystals suitable for SC-XRD were obtained by slow evaporation of a DCM solution of **3-I**. The title compound was previously reported using a similar synthetic procedure, with only ^1H-NMR data provided. [18]

^1H NMR (300 MHz, DMSO-d$_6$) δ 7.47 (dd, J = 7.2, 1.5 Hz, 2H, CH_{arom}), 7.12 (dd, J = 7.5, 1.4 Hz, 2H, CH_{arom}), 6.58 – 6.48 (m, 2H, CH_{arom}), 6.45 – 6.38 (m, 2H, CH_{arom}), 3.57 (s, 2H, CH_2).

^{13}C NMR (75 MHz, DMSO-d$_6$) δ 146.01 (C_2CBi_{arom}), 137.46 (C_3C_{arom}), 133.32 (C_2CH_{arom}), 129.09 (C_2CH_{arom}), 128.83 (C_2CH_{arom}), 127.63 (C_2CH_{arom}), 56.54 (C_2CH_2).

HRMS (ESI): calc'd for $C_{13}H_{11}BiI$ $[M+H]^+$: 502.9704, found: 502.9688.

Compound 3.

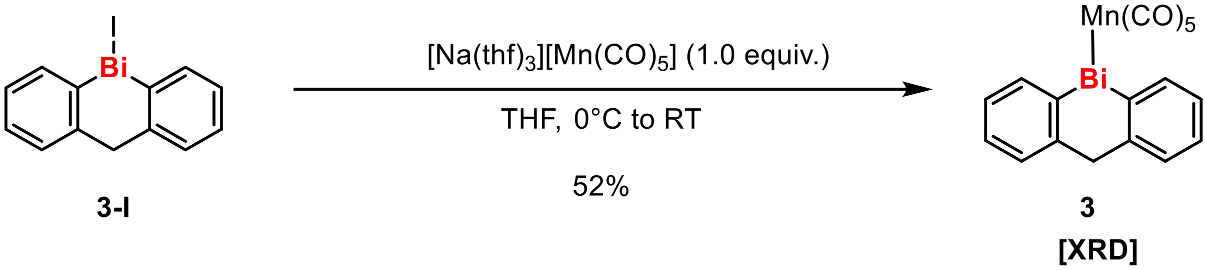

The compound was prepared according to the general procedure for the preparation of bismuth-manganese compounds, starting from bismuth halide **3-I** (125 mg, 0.25 mmol, 1.00 equiv.) and

[Na(thf)$_3$][Mn(CO)$_5$] (0.25 mmol, 108 mg, 1.00 equiv.), yielding the title product (74 mg, 0.13 mmol, 52%) as a pale orange solid. Crystals suitable for SC-XRD were obtained by slow evaporation of a DCM/pentane solution of **3**.

^{1}H NMR (300 MHz, CD_3CN) δ 7.79 (dd, J = 7.2, 1.5 Hz, 2H, CH$_{arom}$), 7.64 (dd, J = 7.5, 1.4 Hz, 2H, CH$_{arom}$), 7.22 – 7.14 (m, 2H, CH$_{arom}$), 7.11 – 7.04 (m, 2H, CH$_{arom}$), 4.13 (s, 2H, CH_2).

^{13}C NMR (75 MHz, CD_3CN) δ 148.47 (C$_3$C$_{arom}$), 139.14 (C$_2$CH$_{arom}$), 129.76 (C$_2$CH$_{arom}$), 129.42 (C$_2$CH$_{arom}$), 127.70 (C$_2$CH$_{arom}$), 57.38 (C$_2$CH$_2$).

HRMS-LIFDI (positive mode) calc'd for $C_{18}H_{10}BiMnO_5$ [M]$^+$: 569.97126, found: 569.9714.
Note: C–Bi carbon atoms are not observable in the ^{13}C NMR spectra (due to line broadening effects arising from ^{209}Bi).

IR: CO stretching modes: = 1901, 2016, 2078 cm^{-1}

Compound 4-Ar.

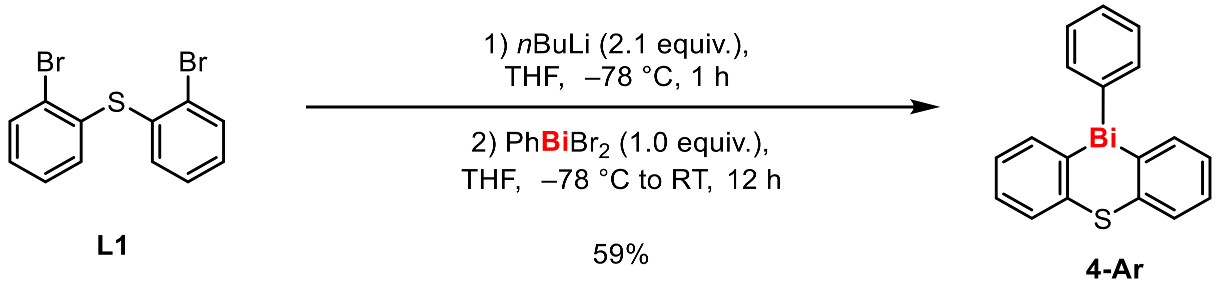

The compound was prepared according to the general procedure for the preparation of triaryl-bismuth precursors, starting from ligand **L1** (688 mg, 2.00 mmol, 1.00 equiv.) and Phenylbismuthdibromide (892 mg, 2.00 mmol, 1.00 equiv.), yielding the title product (555 mg, 1.18 mmol, 59%) as a yellowish oil.

^{1}H NMR (300 MHz, CD_2Cl_2) δ 8.09 – 7.80 (m, 4H, CH$_{arom}$), 7.73 – 7.61 (m, 2H, CH$_{arom}$), 7.49 – 7.37 (m, 3H, CH$_{arom}$), 7.28 – 7.17 (m, 4H, CH$_{arom}$).

^{13}C NMR (75 MHz, CD_2Cl_2) δ 139.65 (C$_2$C$_{arom}$S), 139.25 (C$_2$CH$_{arom}$), 137.85 (C$_2$CH$_{arom}$), 131.79 (C$_2$CH$_{arom}$), 131.05 (C$_2$CH$_{arom}$), 129.73 (C$_2$CH$_{arom}$), 128.67 (C$_2$CH$_{arom}$), 128.50 (C$_2$CH$_{arom}$).

HRMS (ESI): calc'd for $C_{18}H_{14}BiS$ [M+H]$^+$: 471.0615, found: 471.0601.

Note: C–Bi carbon atoms are not observable in the ^{13}C NMR spectra (due to line broadening effects arising from ^{209}Bi).

Compound 4-I.

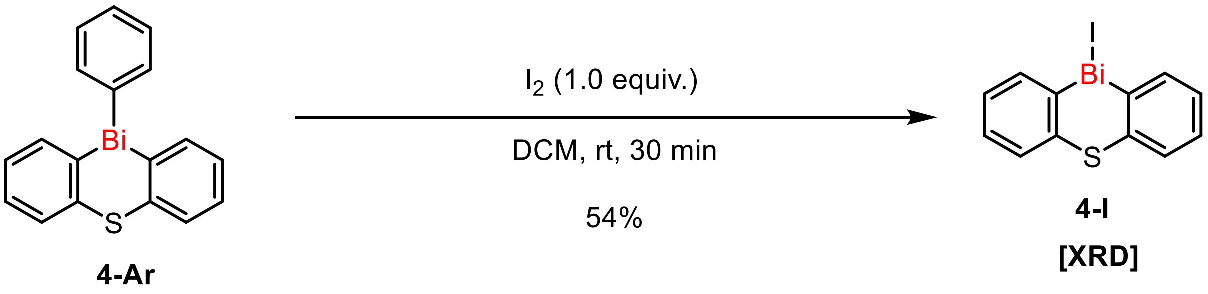

The compound was prepared according to the general procedure for the preparation of bismuth halide precursors, starting from compound **4-Ar** (470.3 mg, 1.00 mmol, 1.00 equiv.) and I_2 (254 mg, 1.00 mmol, 1.00 equiv.), yielding the title product (281 mg, 0.54 mmol, 54%) as a yellowish solid. Crystals suitable for SC-XRD were obtained by slow evaporation of a DCM solution of **4-I**. The title compound was previously reported using a similar synthetic procedure, with only ^{1}H-NMR data provided.[18]

^{1}H NMR (300 MHz, CD_2Cl_2) δ 8.99 (dd, J = 7.5, 1.5 Hz, 1H, CH_{arom}), 7.86 (dd, J = 7.6, 1.3 Hz, 1H, CH_{arom}), 7.52 (td, J = 7.4, 1.3 Hz, 1H, CH_{arom}), 7.30 (td, J = 7.5, 1.5 Hz, 1H, CH_{arom}).

^{13}C NMR (75 MHz, CD_2Cl_2) δ 139.87 (C_2CH_{arom}), 137.43 ($C_2C_{arom}S$), 134.30 (C_2CH_{arom}), 133.09 (C_2CH_{arom}), 129.31 (C_2CH_{arom}).

HRMS (ESI): calc'd for $C_{12}H_9BiIS$ $[M+H]^+$: 520.9268, found: 520.9256.

Note: C–Bi carbon atoms are not observable in the ^{13}C NMR spectra (due to line broadening effects arising from ^{209}Bi).

Compound 4.

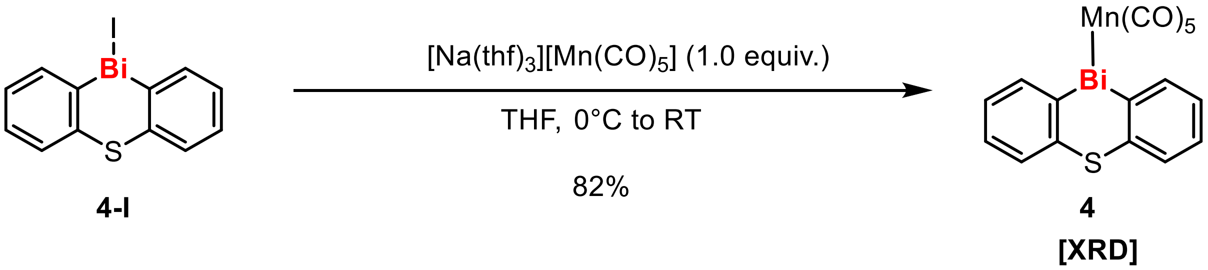

The compound was prepared according to the general procedure for the preparation of bismuth-manganese compounds, starting from bismuth halide **4-I** (130 mg, 0.25 mmol, 1.00 equiv.) and $[Na(thf)_3][Mn(CO)_5]$ (0.25 mmol, 108 mg, 1.00 equiv.), yielding the title product (120 mg, 0.20 mmol, 82%) as a pale orange solid. Crystals suitable for SC-XRD were obtained by slow evaporation of a DCM/pentane solution of **4**.

^{1}H NMR (300 MHz, CD_3CN) δ 7.94 (dd, J = 7.7, 1.4 Hz, 2H, CH_{arom}), 7.87 (dd, J = 7.2, 1.6 Hz, 2H, CH_{arom}), 7.27 – 7.20 (m, 2H, CH_{arom}), 7.18 – 7.12 (m, 2H, CH_{arom}).

^{13}C NMR (75 MHz, CD_3CN) δ 146.45 ($C_2C_{arom}S$), 139.39 (C_2CH_{arom}), 130.09 (C_2CH_{arom}), 130.00 (C_2CH_{arom}), 128.61 (C_2CH_{arom}).

HRMS (EI):EI-MS: calc'd for $C_{16}H_8BiMnO_4S$ [M-(CO)]$^+$: 559.9328 m/z, found: 559.9195 m/z

Note: C–Bi carbon atoms are not observable in the ^{13}C NMR spectra (due to line broadening effects arising from ^{209}Bi).

Elemental analysis: Traces of THF can be difficult to remove from this sample (MW = 588.22 g/mol). The sample analyzed here contained residual amounts of THF. Anal. calc. for $(C_{17}H_8BiMnO_5S)\cdot(THF)_{0.125}$ (597.23 g/mol): C, 35.19; H, 1.52; S, 5.37, found: C, 35.54; H 1.90; S, 4.97.

IR: CO stretching modes: = 1930, 2028, 2076 cm^{-1}

Single-crystal X-ray analysis

Single-crystal X-ray diffraction analyses were performed for compounds **2-I**, **2**, **3-I**, **3**, **4-I** and **4** (Figure S1–6). Graphic representations of the molecular structures as well as selected bond lengths and angles are presented below.

Compound 2-I: [(κ^{2}-$C_{12}H_8SO_2$)BiI]

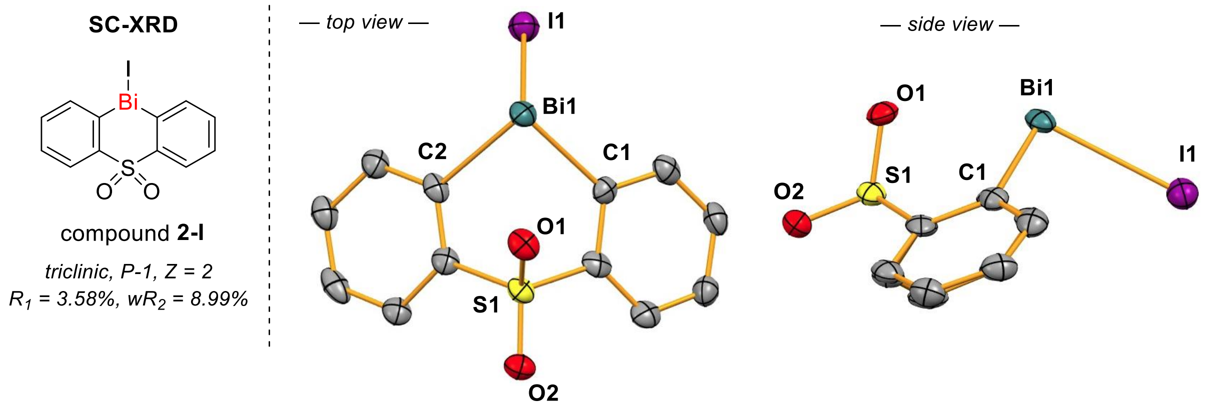

Figure S1. Compound [(κ^{2}-$C_{12}H_8SO_2$)BiI] (**2-I**) was analyzed by single-crystal X-ray diffraction and crystallized in the triclinic space group $P\bar{1}$ with Z = 2. The bismuth center is found in a trigonal pyramidal coordination geometry. Displacement ellipsoids are shown at the 50% probability level. Hydrogen atoms are omitted for clarity. Selected bond lengths (Å) and angles (°): Bi1–I1, 2.8648(6); Bi1–C2, 2.273(7); Bi1–C1, 2.257(7); C2–Bi1–I1, 93.27(19); C1–Bi1–I1, 92.32(18); C1–Bi1–C2, 87.4(3).

Compound 2: [(κ^{2}-$C_{12}H_8SO_2$)Bi(Mn(CO)$_5$)]·(MeCN)

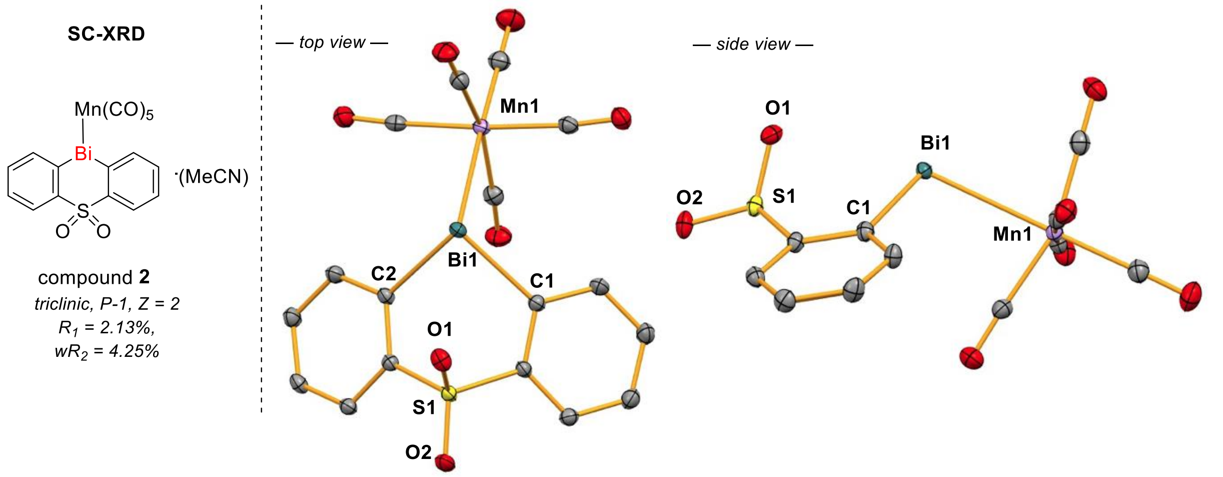

Figure S2. Compound [(κ^{2}-$C_{12}H_8SO_2$)Bi(Mn(CO)$_5$)]·(MeCN) (**2**) was analyzed by single-crystal X-ray diffraction and crystallized in the triclinic space group $P\bar{1}$with Z = 2. The bismuth center is found in a trigonal pyramidal coordination geometry. The Bi–Mn bond length is 2.85 Å, which is in the expected range.[14,17–20] Displacement ellipsoids are shown at the 50% probability level. Hydrogen atoms and one lattice-bound MeCN molecule per formula unit are omitted for clarity. Selected bond lengths (Å) and angles (°): Bi1–Mn1, 2.8514(16); Bi1–C1, 2.302(2); Bi1–C2, 2.300(2); Mn1–CO(*cis*), 1.843(3)-1.872(3); Mn1–CO(*trans*), 1.827(2); C2–Bi1–C1, 86.48(9); C2–Bi1–Mn1, 104.54(7); C1–Bi1–Mn1, 102.05(7).

Compound 3-I: [(κ^2-$C_{13}H_{10}$)BiI]

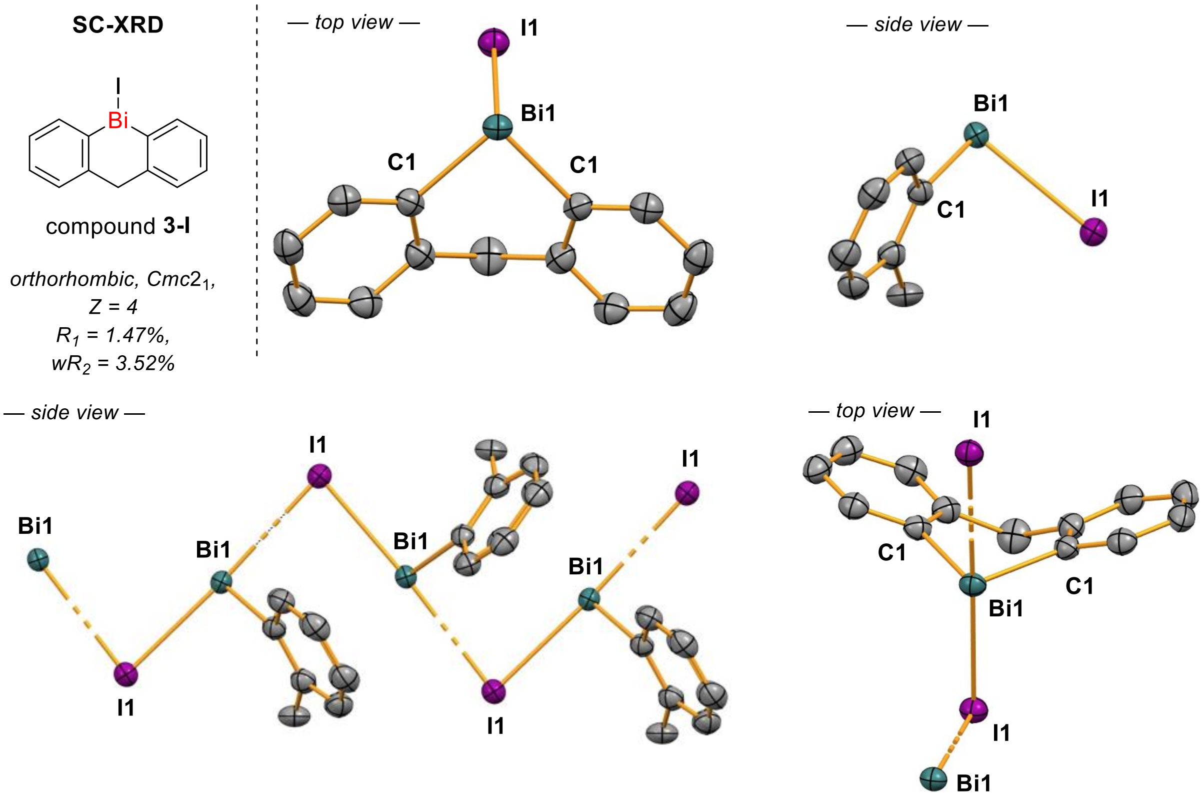

Figure S3. Compound [(κ^2-$C_{13}H_{10}$)BiI] (**3-I**) was analyzed by single-crystal X-ray diffraction and crystallized in the orthorhombic space group *Cmc*2$_1$ with $Z = 4$. The bismuth center is found in a bisphenoidal coordination geometry with an I−Bi−I angle of 176.78(4). Displacement ellipsoids are shown at the 50% probability level. Hydrogen atoms are omitted for clarity. Selected bond lengths (Å) and angles (°): Bi1–C1, 2.234(6); Bi1–I1, 3.0299(10); Bi1–I1(neighboring unit), 3.2988(11); C1–Bi1–C1, 88.7(3); C1–Bi1–I1, 93.44(16); C1–Bi1–I1, 88.86(16); I1–Bi1–I1, 176.78(4); Bi1–I1–Bi1, 83.227(17).

Compound 3: [(κ^2-$C_{13}H_{10}$)Bi(Mn(CO)$_5$)]

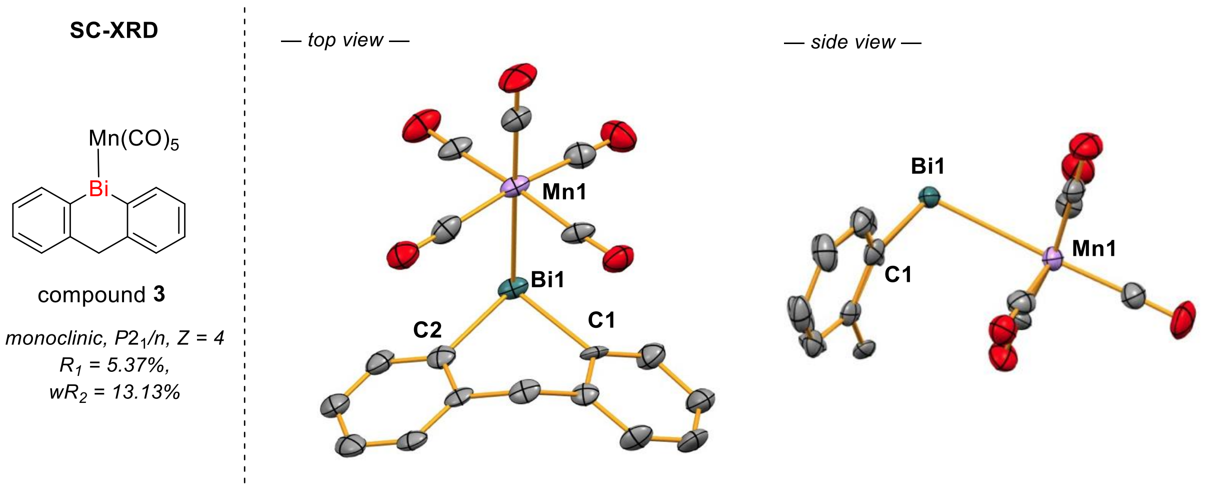

Figure S4. Compound [(κ^2-$C_{13}H_{10}$)Bi(Mn(CO)$_5$)] (**3**) was analyzed by single-crystal X-ray diffraction and crystallized in the monoclinic space group $P2_1/n$ with $Z = 4$. The bismuth center is found in a trigonal pyramidal coordination geometry. The Bi–Mn bond distance is 2.84 Å, which is in the expected bond distance range.[14,17–20] Displacement ellipsoids are shown at the 50% probability level. Hydrogen atoms are omitted for clarity. Selected bond lengths (Å) and angles (°): Bi1–Mn1, 2.8422(19); Bi1–C1, 2.236(13); Bi1–C2, 2.241(11); Mn1–CO(*cis*), 1.838(15)-1.869(15); Mn1–CO(*trans*), 1.827(14); C1–Bi1–Mn1, 101.9(3); C1–Bi1–C2 88.7(4); C2–Bi1–Mn1, 102.8(3).

Compound 4-I: [(κ^2-$C_{12}H_8S$)BiI]

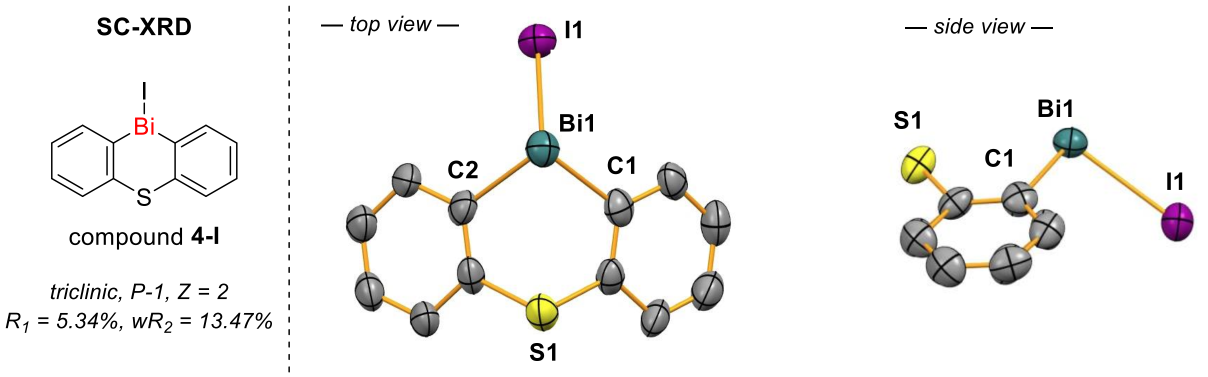

Figure S5. Compound [(κ^2-$C_{12}H_8S$)BiI] (**4-I**) was analyzed by single-crystal X-ray diffraction and crystallized in the triclinic space group $P\bar{1}$ with $Z = 2$. The bismuth center is found in trigonal pyramidal coordination geometry. Displacement ellipsoids are shown at the 50% probability level. Hydrogen atoms are omitted for clarity. Selected bond lengths (Å) and angles (°): Bi1–I1, 2.8422(11); Bi1–C2, 2.252(13); Bi1–C1, 2.246(14); C2–Bi1–I1, 93.3(3); C1–Bi1–I1, 94.8(3); C1–Bi1–C2, 87.5(4). The Bi···S distance is 3.27 Å, which is longer than that reported for related Bi-halide bisaryl S-tethered compounds (X = Cl, Br, I; Bi···S, 2.83 - 2.86 Å).[15,16]

Compound 4: [(κ^2-$C_{12}H_8S$)Bi(Mn(CO)$_5$)]

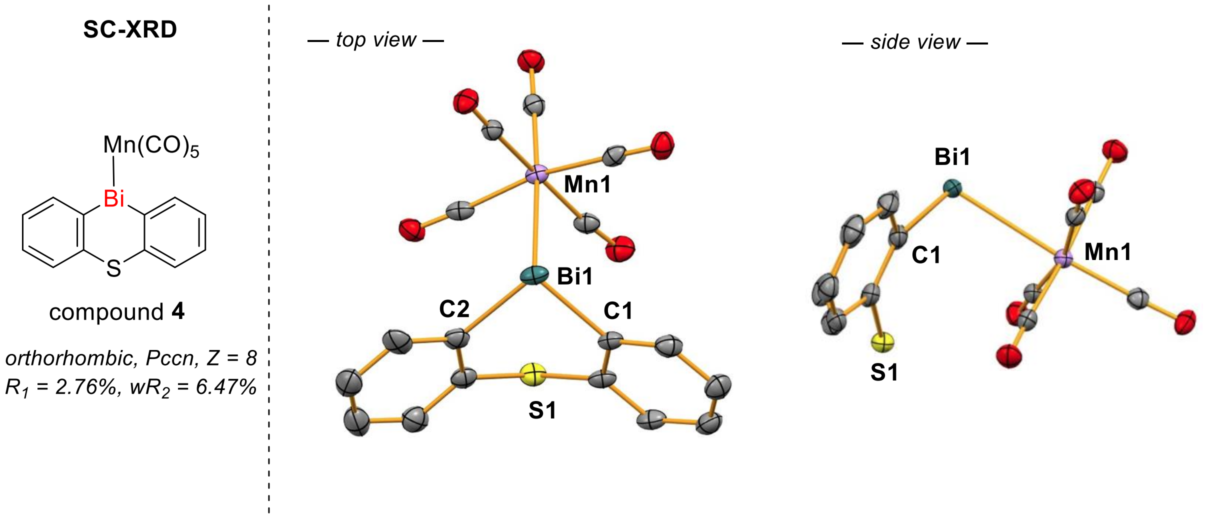

Figure S6. Compound [(κ^2-$C_{12}H_8S$)Bi(Mn(CO)$_5$)] (**4**) was analyzed by single-crystal X-ray diffraction and crystallized in the orthorhombic space group *Pccn* with *Z* = 8. The bismuth center is found in a trigonal pyramidal coordination geometry. The Bi–Mn bond distance is 2.84 Å, which is in the expected bond distance range.[14,17–20] Displacement ellipsoids are shown at the 50% probability level. Hydrogen atoms are omitted for clarity. Selected bond lengths (Å) and angles (°): Bi1–Mn1, 2.8374(7); Bi1–C1, 2.239(5); Bi1–C2, 2.241(5); Mn1–CO(*cis*), 1.848(5)-1.871(5); Mn1–CO(*trans*), 1.828(5); C1–Bi1–Mn1, 100.66(12); C1–Bi1–C2, 90.76(18); C2–Bi1–Mn1, 101.33(12).

In the molecular structure in the solid state, the S atom of **4** points towards the Mn(CO)$_5$ group. DFT calculations suggest that a conformational isomer, in which the S atom points away from the Mn(CO)$_5$ moiety, is energetically accessible (ΔG = 5.1 kcal/mol in the gas phase (level of theory: B3LYP, def2-tzvp, dftd4 dispersion correction)).

Catalytic reactions

General procedure

A J. Young-NMR tube was sequentially charged with substrate (0.05 mmol), benzene-d_6 (0.5 mL) and the selected catalyst (5.0 µmol, 10 mol%). The NMR tube was cover in aluminum foil and placed in a heating block at the indicated temperature and for the indicated time. Upon optimal conversion of the starting material (as judged by ^{1}H NMR spectroscopy), TMB (1,3,5-trimethoxybeneze) was added as an internal standard and the yield was determined.

Collection of analytical data for reported products: a duplicate of the reaction mixture (without TMB added) was filtrated using a silica plug using ethyl acetate as an eluent. The filtrate was concentrated under reduced pressure and then ^{1}H-NMR sample was prepared.

Collection of analytical data for unreported products: a duplicate of the crude reaction mixture (without TMB added) was concentrated under reduced pressure and the residue was purified by column chromatography (silica, 10-20% ethyl acetate in hexane). Fractions containing the product were combined and concentrated under reduced pressure, yielding the corresponding pure isolated product.

Exploratory studies

We performed exploratory studies to test the reactivity of the catalysts on our previously reported cycloisomerization of **S3a** (Table S1)**.** Our studies showed that catalyst **4** delivered similar results to that obtained with compound **1**, while catalysts **2** and **3** presented lower yields (entries 1 to 4). However, much shorter reaction times were obtained when increasing the temperature to 80 °C, which result in catalyst **4** being more efficient than catalyst **1** (entries 5 and 6). Finally, catalyst **4** could also promote the reaction under Blue LED irradiation producing good yields of **S3b** (entry 7).

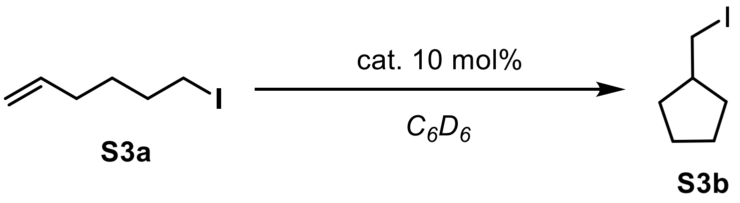

Entry	Catalyst	Conditions[a]	Yield (%)[b]
1	**1**	60 °C, 20 h	96
2	**2**	60 °C, 20 h	45
3	**3**	60 °C, 20 h	89
4	**4**	60 °C, 20 h	95
5	**1**	80 °C, 40 min	78
6	**4**	80 °C, 40 min	94
7	**4**	Blue LEDs, 40 min	69

Table S1. Cycloisomerization of 6-iodo-1-hexene. [(a)]**S3a** (0.025 mmol), C_6D_6 (0.1 M), **catalyst** (10 mol%). [(b)]determined by ^{1}H-NMR using 1,3,5-trimethoxybenzene as an internal standard.

Catalyst loading (substrate 16a, catalyst 4)

We attempted to lower the catalyst loading of the model reaction reported in the manuscript. Reaction of **16a** (14.0 mg, 0.05 mmol, 1 equiv.), catalyst **4** (1.5 mg, 2.5 µmol, 5 mol%), C_6D_6 (0.5 mL) at 80 °C yield 56% of product **16b** after 40 hours reaction time (judged by ^{1}H-NMR spectroscopy). After 120 hours of reaction time, full conversion of the starting material to the desired product was achieved. Thus, lower catalyst loadings still enable the highly selective conversion of the substrate, but require increased reaction times to reach full conversion.

Characterization of products

(iodomethyl)cyclopentane S3b

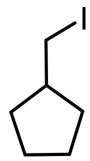

The compound was prepared according to the general procedure catalytic reactions under thermal conditions, starting from 6-iodo-1-hexene (10.4 mg, 0.05 mmol, 1 equiv.), catalyst **4** (3.0 mg, 5.0 µmol, 10 mol%), yielding the title product (94%, NMR yield). The NMR data match previously reported data for the title product. [19]

^{1}H NMR (250 MHz, C_6D_6) δ = 2.74 (d, *J* = 6.9 Hz, 2H), 1.85 – 1.73 (m, 1H), 1.58 – 1.49 (m, 2H), 1.46 –1.25 (m, 4H), 1.01 – 0.83 (m, 2H) ppm.

(cyclopentylideneiodomethyl)trimethylsilane 16b

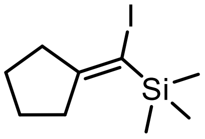

The compound was prepared according to the general procedure catalytic reactions under thermal conditions, starting from **16a** (14.0 mg, 0.05 mmol, 1 equiv.), catalyst **4** (3.0 mg, 5.0 µmol, 10 mol%), yielding the title product (91%, NMR yield). The NMR data match previously reported data for the title product. [6]

^{1}H NMR (250 MHz, C_6D_6) δ 2.48 – 2.33 (m, 2H), 2.18 – 1.91 (m, 2H), 1.61 – 1.43 (m, 2H), 1.36 – 1.26 (m, 2H), 0.26 (s, 9H).

(cyclopentylideneiodomethyl)cyclohexane 19b

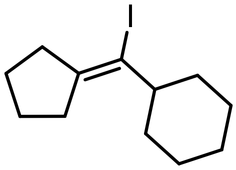

The compound was prepared according to the general procedure catalytic reactions under thermal conditions, starting from **19a** (14.5 mg, 0.05 mmol, 1 equiv.), catalyst **4** (3.0 mg, 5.0 µmol, 10 mol%), yielding the title product (96%, NMR yield). The NMR data match previously reported data for the title product. [6]

^{1}H NMR (300 MHz, $CDCl_3$) δ 2.69 – 2.02 (m, 4H), 1.89 – 1.12 (m, 15H).

(cyclopropyliodomethylene)cyclopentane 20b

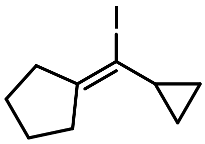

The compound was prepared according to the general procedure catalytic reactions under thermal conditions, starting from **20a** (12.4 mg, 0.05 mmol, 1 equiv.), catalyst **4** (3.0 mg, 5.0 µmol, 10 mol%), yielding the title product (75%, NMR yield). The NMR data match previously reported data for the title product. [6]

^{1}H NMR (250 MHz, C_6D_6) δ 2.89 – 2.70 (m, 1H), 2.33 – 2.23 (m, 2H*overlapped with sideproduct), 2.16 – 2.09 (m, 2H*), 1.57 – 1.42 (m, 5H), 1.40 – 1.31 (m, 2H), 0.80 – 0.61 (m, 2H), 0.54 – 0.43 (m, 2H).

3-(1-iodoethylidene)hexahydro-4*H*-furo[2,3-b]pyran 21b

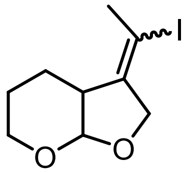

The compound was prepared according to the general procedure catalytic reactions under thermal conditions, starting from **21a** (7.0 mg, 0.025 mmol, 1 equiv.), catalyst **4** (1.5 mg, 2.5 µmol, 10 mol%), yielding the title product (76%, NMR yield). The NMR data match previously reported data for the title product.[7]

^{1}H NMR (300 MHz, $CDCl_3$) δ 5.29 – 5.19 (d, J= 4.0 Hz, 1 H), 4.41 – 4.21 (d, J= 14.2 Hz, 1H), 4.14 – 3.97 (dd, J= 14.2 Hz; 2.3 Hz, 1H), 3.95 – 3.80 (m, 1H), 3.75 – 3.62 (m, 1H), 2.78 – 2.68 (m, 1H), 2.49 (s, 3H), 1.93 – 1.80 (m, 1H), 1.69 – 1.39 (m, 3H).

3-(cyclopentylideneiodomethyl)thiophene 22b

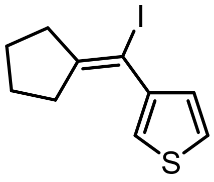

The compound was prepared according to the general procedure catalytic reactions under thermal conditions, starting from **22a** (14.5 mg, 0.05 mmol, 1 equiv.), catalyst **4** (3.0 mg, 5.0 µmol, 10 mol%), yielding the title product (47%, NMR yield). The NMR data match previously reported data for the title product.[6]

^{1}H NMR (300 MHz, $CDCl_3$) δ 7.27 – 7.21 (m, 2H), 7.17 – 7.12 (m, 1H), 2.48 – 2.42 (m, 4H), 1.82 – 1.73 (m, 4H).

(cyclopentylideneiodomethyl)benzene 23b

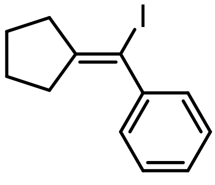

The compound was prepared according to the general procedure catalytic reactions under thermal conditions, starting from **23a** (14.2 mg, 0.05 mmol, 1 equiv.), catalyst **4** (3.0 mg, 5.0 µmol, 10 mol%), yielding the title product (90%, NMR yield). The NMR data match previously reported data for the title product.[20]

^{1}H NMR (300 MHz, $CDCl_3$) δ 7.34 – 7.17 (m, 4H), 2.54 – 2.39 (m, 2H), 2.38 – 2.21 (m, 2H), 1.88 – 1.63 (m, 4H).

This reaction was also performed on the 0.2 mmol scale under otherwise identical conditions, which gave the desired product **23b** in 85% NMR yield.

1-chloro-4-(cyclopentylideneiodomethyl)benzene 24b

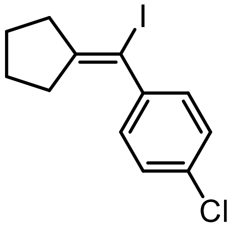

The compound was prepared according to the general procedure catalytic reactions under thermal conditions, starting from **24a** (15.8 mg, 0.05 mmol, 1 equiv.), catalyst **4** (3.0 mg, 5.0 µmol, 10 mol%), yielding the title product (89%, NMR yield). The NMR data match previously reported data for the title product. [20]

^{1}H NMR (300 MHz, $CDCl_3$) δ 7.30 – 7.20 (m, 4H*overlapped with solvent), 2.50 – 2.40 (m, 2H), 2.36 – 2.19 (m, 2H), 1.86 – 1.72 (m, 4H).

1-(cyclopentylideneiodomethyl)-4-(trifluoromethyl)benzene 25b

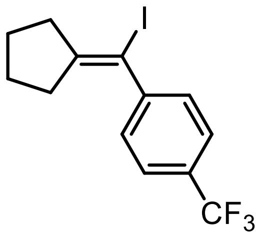

The compound was prepared according to the general procedure catalytic reactions under thermal conditions, starting from **25a** (17.6 mg, 0.05 mmol, 1 equiv.), catalyst **4** (3.0 mg, 5.0 μmol, 10 mol%), yielding the title product (87%, NMR yield). The NMR data match previously reported data for the title product. [20]

^{1}H NMR (300 MHz, $CDCl_3$) δ 7.57 – 7.39 (m, 4H), 2.64 – 2.11 (m, 4H), 1.92 – 1.63 (m, 4H).

^{19}F NMR (282 MHz, $CDCl_3$) δ –62.58.

1-(cyclopentylideneiodomethyl)-4-methoxybenzene 26b

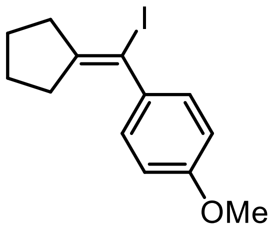

The compound was prepared according to the general procedure catalytic reactions under thermal conditions, starting from **26a** (15.6 mg, 0.05 mmol, 1 equiv.), catalyst **4** (3.0 mg, 5.0 μmol, 10 mol%), yielding the title product (92%, NMR yield). The NMR data match previously reported data for the title product. [20]

^{1}H NMR (300 MHz, $CDCl_3$) δ 7.30 – 7.17 (m, 2H), 6.86 – 6.77 (m, 2H), 3.80 (s, 3H), 2.52 – 2.22 (m, 4H), 1.87 – 1.66 (m, 4H).

2-(4-cyclopentylidene-4-iodobutoxy)tetrahydro-2H-pyran 27b

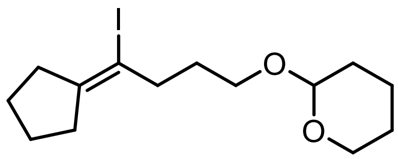

The compound was prepared according to the general procedure catalytic reactions under thermal conditions, starting from **27a** (17.5 mg, 0.05 mmol, 1 equiv.), catalyst **4** (3.0 mg, 5.0 μmol, 10 mol%), yielding the title product (97%, NMR yield). The NMR data match previously reported data for the title product. [6]

^{1}H NMR (250 MHz, C_6D_6) δ 4.71 – 4.54 (m, 1H), 3.94 – 3.76 (m, 2H), 3.48 – 3.28 (m, 2H), 2.45 – 2.19 (m, 4H), 2.06 – 1.96 (m, 2H), 1.75 – 1.43 (m, 10H), 1.41 – 1.28 (m, 4H).

1-(cyclopentylideneiodomethyl)-3-fluorobenzene 28b

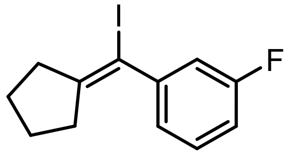

The compound was prepared according to the general procedure catalytic reactions under thermal conditions, starting from **28a** (15.1 mg, 0.05 mmol, 1 equiv.), catalyst **4** (3.0 mg, 5.0 μmol, 10 mol%), yielding the title product (13.1 mg, 0.043 mmol, 87% isolated yield) as a colorless oil.

^{1}H NMR (300 MHz, $CDCl_3$) δ 7.32 – 7.20 (m, 1H*overlapped with solvent), 7.12 – 6.84 (m, 3H), 2.55 – 2.41 (m, 2H), 2.38 – 2.22 (m, 2H), 1.87 – 1.70 (m, 4H).

^{13}C NMR (75 MHz, $CDCl_3$) δ 163.79 (d, J = 244.2 Hz), 153.71, 143.03, 129.48 (d, J = 8.6 Hz), 124.63 (d, J = 2.4 Hz), 115.89 (d, J = 22.1 Hz), 114.30 (d, J = 21.8 Hz), 41.87, 33.75, 28.63, 25.84.

^{19}F NMR (282 MHz, $CDCl_3$) δ –113.31.

HRMS (ESI): calc'd for ($C_{12}H_{13}FI$) $[M+H]^+$ 303.0046, found 303.0051.

4-cyclopentylidene-4-iodobutan-1-ol 29b

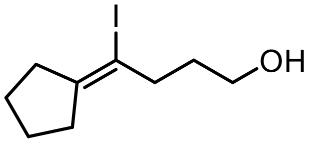

The compound was prepared according to the general procedure catalytic reactions under thermal conditions, starting from **29b** (13.3 mg, 0.05 mmol, 1 equiv.), catalyst **4** (3.0 mg, 5.0 μmol, 10 mol%), yielding the title product (11.7 mg, 0.044 mmol, 88% isolated yield). The NMR data match previously reported data for the title product.[6]

^{1}H NMR (250 MHz, C_6D_6) δ 3.71 – 3.05 (m, 2H), 2.44 – 2.18 (m, 3H), 2.18 – 1.85 (m, 3H), 1.67 – 1.11 (m, 9H).

5-cyclopentylidene-5-iodopentyl (R)-2-(6-methoxynaphthalen-2-yl)propanoate 30b

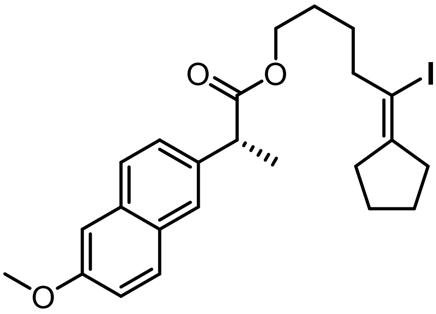

The compound was prepared according to the general procedure catalytic reactions under thermal conditions, starting from **30a** (24.6 mg, 0.05 mmol, 1 equiv.), catalyst **4** (3.0 mg, 5.0 μmol, 10 mol%), yielding the title product (21.9 mg, 0.045 mmol, 89% isolated yield) as a colorless oil.

^{1}H NMR (300 MHz, $CDCl_3$) δ 7.74 – 7.62 (m, 3H), 7.46 – 7.34 (m, 1H), 7.16 – 7.10 (m, 2H), 4.09 (t, J = 6.1 Hz, 2H), 3.96 – 3.76 (m, 4H), 2.48 – 2.17 (m, 5H), 1.90 – 1.33 (m, 12H).

^{13}C NMR (75 MHz, $CDCl_3$) δ 174.84, 157.77, 149.33, 135.93, 133.84, 129.44, 129.10, 127.26, 126.43, 126.08, 119.07, 105.77, 96.37, 64.73, 55.46, 45.69, 41.43, 41.20, 31.78, 28.39, 27.48, 25.84, 25.77, 18.65.

HRMS (ESI): calc'd for ($C_{24}H_{29}IO_3Na$) $[M+Na]^+$ 515.1054, found 515.1058.

5-cyclopentylidene-5-iodopentyl 2-(4-isobutylphenyl)propanoate 31b

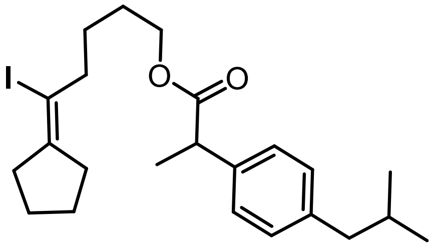

The compound was prepared according to the general procedure catalytic reactions under thermal conditions, starting from **31a** (23.4 mg, 0.05 mmol, 1 equiv.), catalyst **4** (3.0 mg, 5.0 μmol, 10 mol%), yielding the title product (20.1 mg, 0.043 mmol, 86% isolated yield) as a colorless oil.

^{1}H NMR (300 MHz, $CDCl_3$) δ 7.21 (d, J = 8.0 Hz, 2H), 7.09 (d, J = 8.2 Hz, 2H), 4.17 – 4.00 (m, 2H), 3.69 (d, J = 7.1 Hz, 1H), 2.50 – 2.37 (m, 3H), 2.32 – 2.22 (m, 3H), 2.01 – 1.05 (m, 14H), 0.90 (d, J = 6.6 Hz, 6H).

^{13}C NMR (75 MHz, $CDCl_3$) δ 174.93, 149.35, 140.60, 137.99, 129.44, 127.32, 96.45, 64.62, 45.37, 45.21, 41.46, 41.21, 31.85, 30.32, 28.43, 27.47, 25.87, 25.73, 22.55, 18.65.

HRMS (ESI): calc'd for ($C_{23}H_{33}IO_2Na$) $[M+Na]^+$ 491.1417, found 491.1418.

5-cyclopentylidene-5-iodopentyl 2-(1-(4-chlorobenzoyl)-5-methoxy-2-methyl-1H-indol-3-yl)acetate 32b

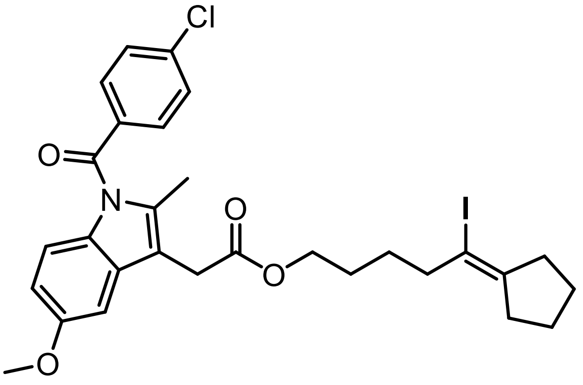

The compound was prepared according to the general procedure catalytic reactions under thermal conditions, starting from **32a** (30.9 mg, 0.05 mmol, 1 equiv.), catalyst **4** (3.0 mg, 5.0 μmol, 10 mol%), yielding the title product (25.6 mg, 0.041 mmol, 83% isolated yield) as a colorless oil.

^{1}H NMR (300 MHz, $CDCl_3$) δ 7.66 (d, J = 8.5 Hz, 2H), 7.47 (d, J = 6.7 Hz, 2H), 6.97 (d, J = 4.7 Hz, 1H), 6.87 (d, J = 9.0 Hz, 1H), 6.66 (dd, J = 9.0, 2.6 Hz, 1H), 4.12 (t, J = 6.2 Hz, 2H), 3.84 (s, 3H), 3.67 (s, 2H), 2.65 – 2.03 (m, 9H), 1.82 – 1.46 (m, 8H).

^{13}C NMR (75 MHz, $CDCl_3$) δ 168.43, 156.22, 149.49, 139.39, 136.05, 134.11, 131.32, 130.98, 130.83, 129.27, 115.10, 112.84, 111.84, 101.47, 96.26, 65.03, 55.89, 41.46, 41.19, 31.85, 30.59, 28.41, 27.54, 25.85, 13.56.

HRMS (ESI): calc'd for ($C_{29}H_{32}ClINO_4$) $[M+H]^+$ 620.1059, found 620.1060.

Additional substrates

Catalyst **4** was tested towards a range of additional substrates under the standard conditions (10 mol% catalyst, benzene, 80 °C, 20 h).

Attempts to target six- and seven-membered rings by starting from alkynes with iodo-pentyl and iodo-hexyl substituents (e.g. $I(CH_2)_nCCPh$, n = 5,6) have not led to the desired products in significant yields to date.

Tosylates such as $TsO(CH_2)_4CCPh$ did not give the desired cyclization product with the standard protocol reported in this work.

Bromides such as $Br(CH_2)_4CCPh$ gave the desired cyclization product in low spectroscopic yield of 10% (essentially, a stoichiometric reaction). Extensions of the reaction time to 72 h slightly increased the yield to 16%. Performing the reaction in the presence of 1 equivalent of LiI or $N(nBu)_4I$ (with respect to the substrate) for 20 h at 80 °C also slightly increased the yield of cyclization products to 15-16%.

In intermolecular reactions, *n*BuI and *i*PrI were reacted with $HCCSiMe_3$. While only minor amounts of the product *n*BuHC=$CISiMe_3$were detected by mass spectrometry in the case of the primary alkyl iodide, the secondary alkyl iodide gave the desired coupling product *i*PrHC=$CISiMe_3$[21] in low, but significant spectroscopic yield of 27%.

The substrates $I(CH_2)_4CC(p\text{-}C_4H_4X)$ (X = NMe_2, CN) gave low yields (26%) of the desired cyclized amine (X = NMe_2) and moderate yields (42%) of the desired cyclized cyano compound (X = CN).

Mechanistic considerations

Based on the observations in the catalyzed reactions, stoichiometric reactions between **4** and **16a** as well as EPR spectroscopic reaction monitoring along with literature precedents on related reactions[19] let us suggest the mechanistic scenario summarized in Scheme S2, covering a catalytic cycles (A) and (B). The formation of bismuth alkyl species (rather than the corresponding bismuth-iodo species) is expected, as the iodo compound would be the starting material **4-I**, which could not be detected in the course of these reactions. In addition, the formation of [Bi]–R + [Mn]–I is mildly exergonic (ΔG = –6.1 kcal·mol^{-1}), while the formation of [Bi]–I + [Mn]–R is slightly endergonic (ΔG = +1.1 kcal·mol^{-1}) according to DFT calculations, when catalyst **4** and substrate **16a** are considered. The manganese iodide species $MnI(CO)_5$ was detected by IR spectroscopy from stoichiometric reactions between **4** and **23a** (a closely related cyclization substrate, see main part).[22] Significant amounts of bismuth-containing degradation products (e.g.: low-valent bismuth species in the form of a dark precipitate or a dibismuthane[23]) were not observed, suggesting that a radical chain mechanism that is disjunct of any molecular bismuth compounds is not a major propagation pathway. In agreement with this, NMR spectroscopic analyses of stoichiometric reactions between **4** and **16a** showed two bismuth species with NOESY cross peaks between the phenyl groups and the CH_2 groups, which were tentatively assigned to the suggested intermediates **Int1** and **Int2**.

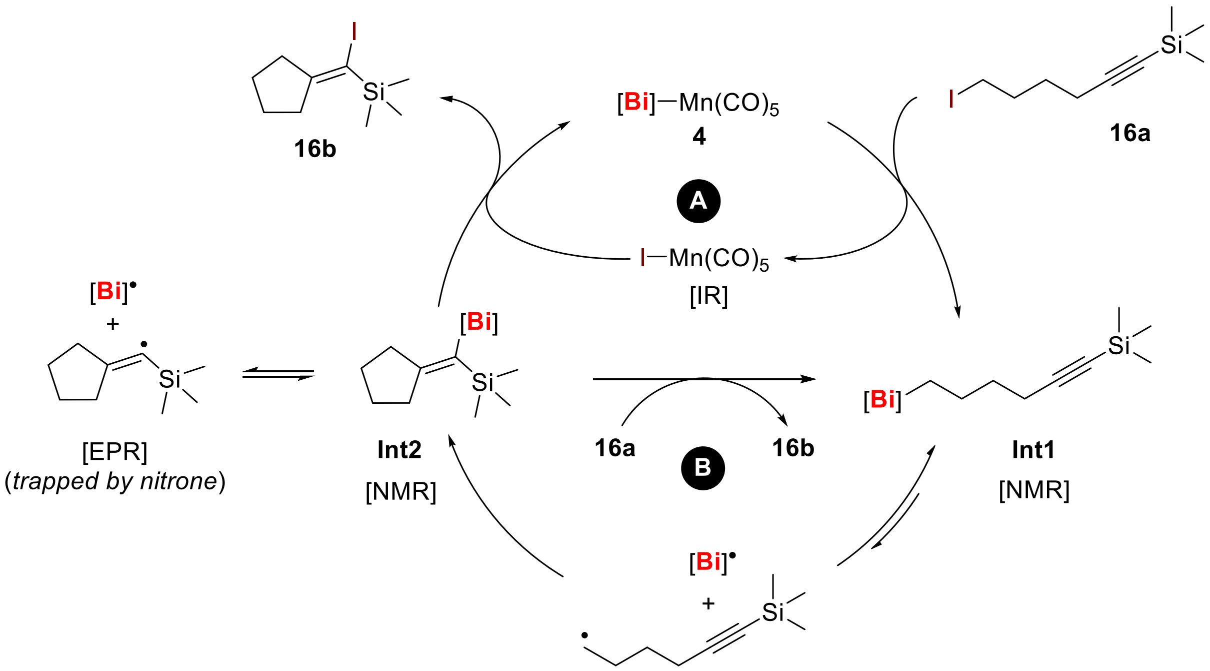

Scheme S2. Proposed mechanistic scenario for the transformation of **16a** into **16b** in the presence of catalytic amounts of **4**, with $MnI(CO)_5$ (A) or **16a** (B) as the iodine atom transfer reagent in the step of product formation.

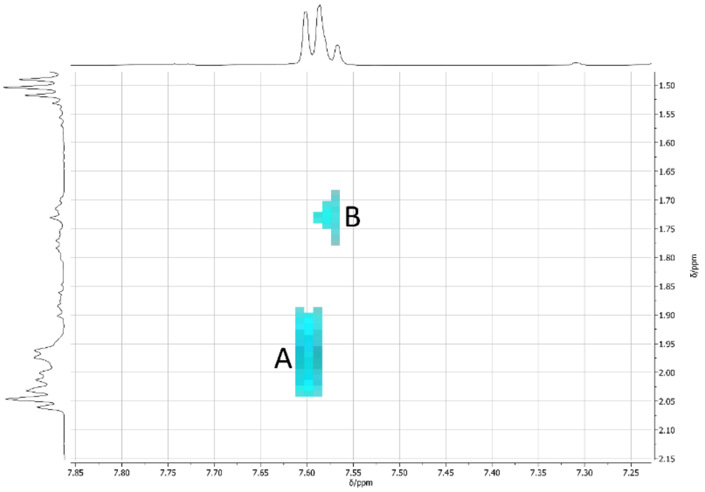

Figure S7. Excerpt of the ^{1}H ^{1}H-NOESY-NMR spectrum (500 MHz) of a reaction between compound **23a** and 1 equiv. of the catalyst **4** after 1 h of heating to 80 °C.

EPR spectroscopic measurements

EPR spectroscopic experiments were performed with an MS5000 X-band CW EPR spectrometer. All experiments were carried out at room temperature under inert conditions. Simulations of the EPR spectra were performed with the EasySpin software package, running in the MATLAB software environment (R2022a; 9.12.0.1927505) provided by MathWorks.[24]

An EPR tube was charged with **16a** (7.0 mg, 0.025 mmol, 10 equiv.), phenyl *N*-tert-butyl nitrone (PBN) (44 mg, 0.025 mmol, 10 equiv.) and catalyst **4** (1.5 mg, 2.5 µmol, 1.0 equiv.) in C_6D_6 (0.50 mL). The tube was placed on a heating block heated at 70 °C for 5 minutes and then characterized by EPR spectroscopy. Further details of the EPR spectroscopic experiments are given in the main part.

Note: a bismuth-centered radical was not detected. This is typically explained due to the short life time, low concentration, relatively high temperature, and (potentially) fast relaxation times due to spin orbit coupling of such species.[25]

Computational screening of catalyst candidates

Computational details

All geometry optimizations and single point energy calculations were performed using the Gaussian16[26] suite of programs at the M06-L+GD3/def2-TZVP$^{(C,H,N,O,S,F,Si)}$ / LanL2DZ$^{(Bi,Mn,I)}_{(Benzene,SMD)}$ // M06-L+GD3/def2-SVP$^{(C,H,N,O,S,F,Si)}$ / LanL2DZ$^{(Bi,Mn,I)}$ level of theory.[27–34] Optimzed structures for catalyst screenings are shown in Chart S1 and Chart S2. All computed values are presented in Table S2. Cartesian coordinates are listed at the end of this file.

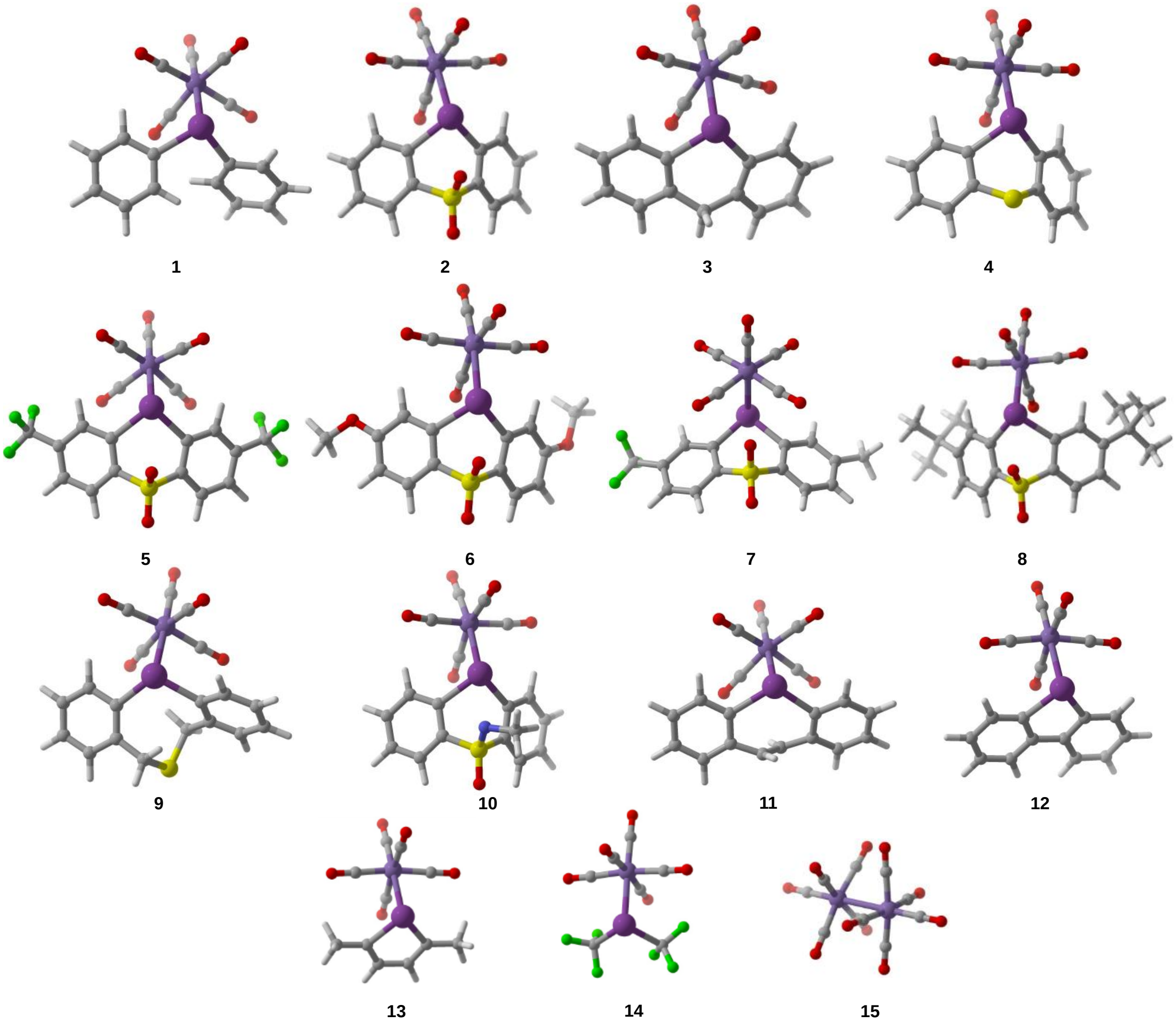

Chart S1. Optimized structures of catalyst candidates **1–15**.

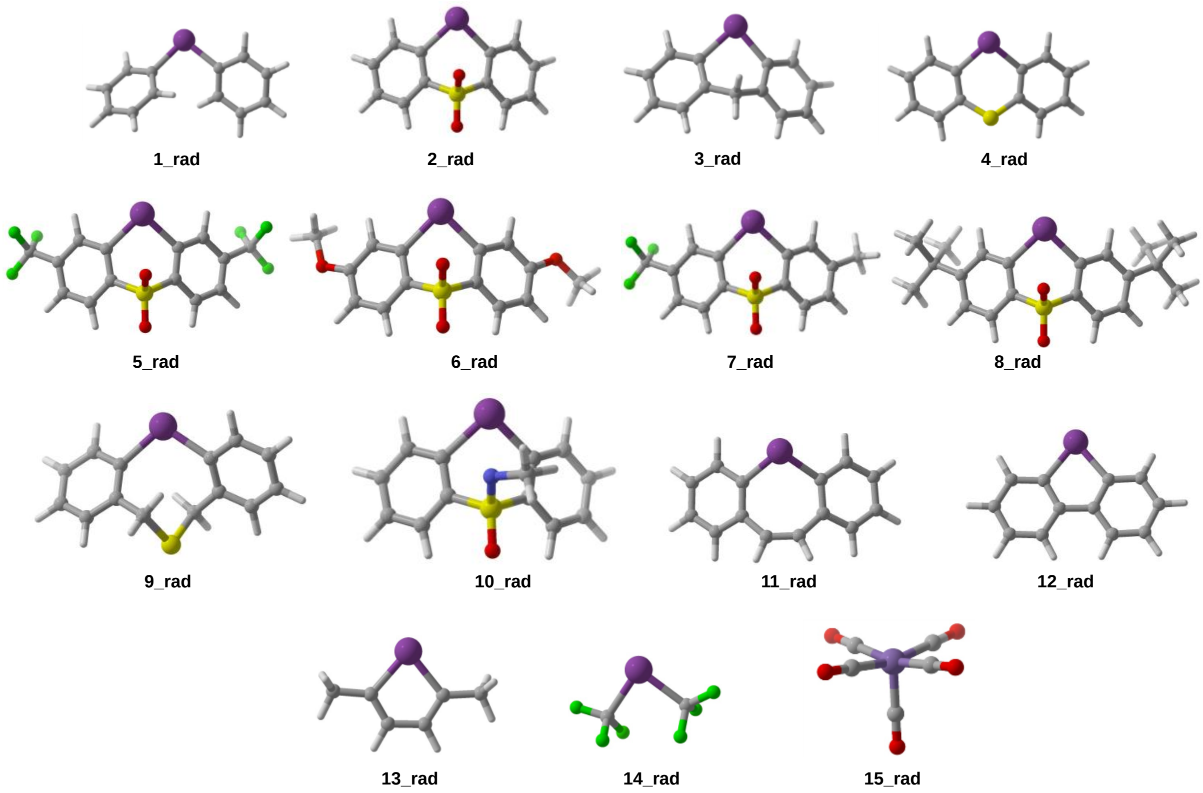

Chart S2. Optimized structures of radicals of catalyst candidates **1–15**.

Structure	G_{corr}	G_{298} gas phase	E_0 benzene	G_{298} benzene
**1**	0.165140	-1138.427	-1139.7603	-1139.5951
**1_rad**	0.137022	-468.17018	-468.8077	-468.67067
**2**	0.15367	-1685.561	-1687.238	-1687.0844
**2_rad**	0.126519	-1015.3067	-1016.2911	-1016.1645
**3**	0.174219	-1176.5034	-1177.8817	-1177.7074
**3_rad**	0.147063	-506.25132	-506.93525	-506.78819
**4**	0.146295	-1535.3145	-1536.7573	-1536.611
**4_rad**	0.118139	-865.06263	-865.80937	-865.69123
**5**	0.150669	-2359.0669	-2361.5231	-2361.3724
**5_rad**	0.124265	-1688.8099	-1690.5742	-1690.45
**6**	0.212796	-1914.3578	-1916.3529	-1916.1401
**6_rad**	0.185048	-1244.1006	-1245.4031	-1245.218
**7**	0.176412	-2061.5769	-2063.7095	-2063.5331
**7_rad**	0.148561	-1391.3206	-1392.7602	-1392.6116
**8**	0.36687	-1999.6017	-2001.8255	-2001.4586
**8_rad**	0.337008	-1329.3436	-1330.8735	-1330.5365
**9**	0.200922	-1613.8213	-1615.4029	-1615.202
**9_rad**	0.172593	-943.56590	-944.45113	-944.27854
**10**	0.190183	-1704.9139	-1706.6332	-1706.443
**10_rad**	0.162916	-1034.6582	-1035.6839	-1035.521
**11**	0.177846	-1214.5558	-1215.9786	-1215.8008
**11_rad**	0.150709	-544.29743	-545.02591	-544.87521
**12**	0.148153	-1137.2655	-1138.5747	-1138.4265
**12_rad**	0.120452	-467.00752	-467.62278	-467.50233
**13**	0.110197	-908.80498	-909.83915	-909.72896
**13_rad**	0.082902	-238.54619	-238.88635	-238.80345
**14**	0.016097	-1350.3346	-1351.8241	-1351.808
**14_rad**	-0.013267	-680.07046	-680.86647	-680.87974
**15**	0.032522	-1340.4576	-1341.8334	-1341.8009
**15_rad**	0.001976	-670.20823	-670.87912	-670.87714
**$Mn(CO)_5$alkyl**	0.212028	-1312.2821	-1313.6565	-1313.4445
**$Mn(CO)_5I$**	0.001123	-681.64199	-682.31652	-682.31540
**$Bi(C_6H_4)_2S$alkyl**	0.327292	-1507.1525	-1508.6005	-1508.27317
**$Bi(C_6H_4)_2SI$**	0.115952	-876.50113	-877.24844	-877.13248
**I-alkyl**	0.183824	-653.46921	-654.15164	-653.96782

Table S2. G_{corr} is the thermal correction to Gibbs Free Energy and $G_{298\ gas\ phase}$ is the sum of electronic and thermal free energies at the M06-L+GD3/def2svp level of theory in gas phase (values in hartree); $E_{0\ benzene}$ is the electronic energy obtained at the M06-L+GD3/def2tzvp/LANL2DZ(Bi)(SMD)//M06L+GD3/def2svp level of theory in benzene as solvent; SMD, solvation model based on density (values in hartree); $G_{298\ benzene}$ is the sum of $E_{0\ benzene}$ and G_{corr} (values in hartree). Alkyl = $(CH_2)_4CCSiMe_3$.

References

(1) Dolomanov, O. V.; Bourhis, L. J.; Gildea, R. J.; Howard, J. A. K.; Puschmann, H. OLEX2 : a complete structure solution, refinement and analysis program. *J Appl Crystallogr* **2009**, *42*, 339–341.

(2) Sheldrick, G. M. A short history of SHELX. *Acta Crystallogr. A* **2008**, *64*, 112–122.

(3) Sheldrick, G. M. Crystal structure refinement with SHELXL. *Acta Crystallogr. C* **2015**, *71*, 3–8.

(4) Ingenito, R.; Burton, C.; Langella, A.; Chen, X.; Zytko, K.; Pessi, A.; Wang, J.; Bianchi, E. Novel potent apoA-I peptide mimetics that stimulate cholesterol efflux and pre-beta particle formation in vitro. *Bioorg. Med. Chem. Lett.* **2010**, *20*, 236–239.

(5) Hodgson, D. M.; Labande, A. H.; Pierard, F. Y. T. M.; Expósito Castro, M. A. The scope of catalytic enantioselective tandem carbonyl ylide formation-intramolecular 3 + 2 cycloadditions. *J. Org. Chem.* **2003**, *68*, 6153–6159.

(6) Shen, Y.; Cornella, J.; Juliá-Hernández, F.; Martin, R. Visible-Light-Promoted Atom Transfer Radical Cyclization of Unactivated Alkyl Iodides. *ACS Catal.* **2017**, *7*, 409–412.

(7) Yang, X.; Yu, W. Promoting effect of water on light and phenanthroline-diphosphine Cu(I) complex-initiated iodine atom transfer cyclisation. *Chem. Commun.* **2022**, *58*, 11693–11696.

(8) Monks, B. M.; Cook, S. P. Palladium-catalyzed alkyne insertion/Suzuki reaction of alkyl iodides. *J. Am. Chem. Soc.* **2012**, *134*, 15297–15300.

(9) Willett, B. C.; Moore, W. M.; Salajegheh, A.; Peters, D. G. Electrochemical reduction and intramolecular cyclization of 6-iodo-1-phenyl-1-hexyne and 6-bromo-1-phenyl-1-hexyne at mercury cathodes in dimethylformamide. *J. Am. Chem. Soc.* **1979**, *101*, 1162–1167.

(10) Zhou, S.; Yuan, F.; Guo, M.; Wang, G.; Tang, X.; Zhao, W. Switchable Synthetic Strategy toward Trisubstituted and Tetrasubstituted Exocyclic Alkenes. *Org. Lett.* **2018**, *20*, 6710–6714.

(11) Zhong, Z.; Wang, Z.-Y.; Ni, S.-F.; Dang, L.; Lee, H. K.; Peng, X.-S.; Wong, H. N. C. Ligand-Free Iron-Catalyzed Carbon (sp^2)-Carbon (sp^2) Oxidative Homo-Coupling of Alkenyllithiums. *Org. Lett.* **2019**, *21*, 700–704.

(12) Osi, A.; Mahaut, D.; Tumanov, N.; Fusaro, L.; Wouters, J.; Champagne, B.; Chardon, A.; Berionni, G. Taming the Lewis Superacidity of Non-Planar Boranes: C-H Bond Activation and Non-Classical Binding Modes at Boron. *Angew. Chem. Int. Ed.* **2022**, *61*, e202112342.

(13) Corrie, T. J. A.; Ball, L. T.; Russell, C. A.; Lloyd-Jones, G. C. Au-Catalyzed Biaryl Coupling To Generate 5- to 9-Membered Rings: Turnover-Limiting Reductive Elimination versus π-Complexation. *J. Am. Chem. Soc.* **2017**, *139*, 245–254.

(14) Wood, T. K.; Piers, W. E.; Keay, B. A.; Parvez, M. Synthesis and comparative characterization of 9-boraanthracene, 5-boranaphthacene, and 6-borapentacene stabilized by the H_2IMes carbene. *Chem. Eur. J.* **2010**, *16*, 12199–12206.

(15) Olah, G. A.; Marinez, E. R.; Prakash, G. K. S. Trifluoromethanesulfonic Acid Catalyzed Preparation of Symmetrical Diaryl Sulfoxides from Arenes and Thionyl Chloride. *Synlett* **1999**, *1999*, 1397–1398.

(16) Banerjee, S.; Karunananda, M. K.; Bagherzadeh, S.; Jayarathne, U.; Parmelee, S. R.; Waldhart, G. W.; Mankad, N. P. Synthesis and characterization of heterobimetallic complexes with direct Cu-M bonds (M = Cr, Mn, Co, Mo, Ru, W) supported by N-heterocyclic carbene ligands: a toolkit for catalytic reaction discovery. *Inorg. Chem.* **2014**, *53*, 11307–11315.

(17) Jurrat, M.; Maggi, L.; Lewis, W.; Ball, L. T. Modular bismacycles for the selective C-H arylation of phenols and naphthols. *Nat. Chem.* **2020**, *12*, 260–269.

(18) Suzuki, H.; Murafuji, T.; Azuma, N. Synthesis and reactions of some new heterocyclic bismuth-(III) and -(V) compounds. 5,10-Dihydrodibenzo[b,e]bismine and related systems. *J. Chem. Soc., Perkin Trans. 1* **1992**, 1593.

(19) Ramler, J.; Krummenacher, I.; Lichtenberg, C. Bismuth Compounds in Radical Catalysis: Transition Metal Bismuthanes Facilitate Thermally Induced Cycloisomerizations. *Angew. Chem. Int. Ed.* **2019**, *58*, 12924–12929.

(20) Weng, W.-Z.; Liang, H.; Liu, R.-Z.; Ji, Y.-X.; Zhang, B. Visible-Light-Promoted Manganese-Catalyzed Atom Transfer Radical Cyclization of Unactivated Alkyl Iodides. *Org. Lett.* **2019**, *21*, 5586–5590.

(21) Khripach, V. A.; Zhabinskiy, V. N.; Olkhovick, V. K. Highly stereoselective synthesis of steroidal 22α-allylic alcohols via 22-aldehydes and 1-silyl-1-iodo-1-alkenes: A new efficient route to the side chain construction of brassinolide. *Tetrahedron Letters* **1990**, *31*, 4937–4940.

(22) Abel, E. W.; Wilkinson, G. 291. Carbonyl halides of manganese and some related compounds. *J. Chem. Soc.* **1959**, 1501.

(23) Ramler, J.; Krummenacher, I.; Lichtenberg, C. Well-Defined, Molecular Bismuth Compounds: Catalysts in Photochemically Induced Radical Dehydrocoupling Reactions. *Chem. Eur. J.* **2020**, *26*, 14551–14555.

(24) Stoll, S.; Schweiger, A. EasySpin, a comprehensive software package for spectral simulation and analysis in EPR. *J. Magn. Reson.* **2006**, *178*, 42–55.

(25) Ishida, S.; Hirakawa, F.; Furukawa, K.; Yoza, K.; Iwamoto, T. Persistent antimony- and bismuth-centered radicals in solution. *Angew. Chem. Int. Ed.* **2014**, *53*, 11172–11176.

(26) Frisch, M. J.; Trucks, G. W.; Schlegel, H. B.; Scuseria, G. E.; Robb, M. A.; Cheeseman, J. R.; Scalmani, G.; Barone, V.; Petersson, G. A.; Nakatsuji, H.; Li, X.; Caricato, M.; Marenich, A. V.; Bloino, J.; Janesko, B. G.; Gomperts, R.; Mennucci, B.; Hratchian, H. P.; Ortiz, J. V.; Izmaylov, A. F.; Sonnenberg, J. L.; Williams-Young, D.; Ding, F.; Lipparini, F.; Egidi, F.; Goings, J.; Peng, B.; Petrone, A.; Henderson, T.; Ranasinghe, D.; Zakrzewski, V. G.; Gao, J.; Rega, N.; Zheng, G.; Liang, W.; Hada, M.; Ehara, M.; Toyota, K.; Fukuda, R.; Hasegawa, J.; Ishida, M.; Nakajima, T.; Honda, Y.; Kitao, O.; Nakai, H.; Vreven, T.; Throssell, K.; Montgomery, J. A., Jr.; Peralta, J. E.; Ogliaro, F.; Bearpark, M. J.; Heyd, J. J.; Brothers, E. N.; Kudin, K. N.; Staroverov, V. N.; Keith, T. A.; Kobayashi, R.; Normand, J.; Raghavachari, K.; Rendell, A. P.; Burant, J. C.; Iyengar, S. S.; Tomasi, J.; Cossi, M.; Millam, J. M.; Klene, M.; Adamo, C.; Cammi, R.; Ochterski, J. W.; Martin, R. L.; Morokuma, K.; Farkas, O.; Foresman, J. B.; Fox, D. J. *Gaussian 16*; Gaussian, Inc., 2016.

(27) Cramer, C. J.; Truhlar, D. G. Density functional theory for transition metals and transition metal chemistry. *Phys. Chem. Chem. Phys.* **2009**, *11*, 10757–10816.

(28) Marenich, A. V.; Cramer, C. J.; Truhlar, D. G. Universal solvation model based on solute electron density and on a continuum model of the solvent defined by the bulk dielectric constant and atomic surface tensions. *J. Phys. Chem. B* **2009**, *113*, 6378–6396.

(29) Weigend, F. Accurate Coulomb-fitting basis sets for H to Rn. *Phys. Chem. Chem. Phys.* **2006**, *8*, 1057–1065.

(30) Weigend, F.; Ahlrichs, R. Balanced basis sets of split valence, triple zeta valence and quadruple zeta valence quality for H to Rn: Design and assessment of accuracy. *Phys. Chem. Chem. Phys.* **2005**, *7*, 3297–3305.

(31) Zhao, Y.; Truhlar, D. G. Density functionals with broad applicability in chemistry. *Acc. Chem. Res.* **2008**, *41*, 157–167.

(32) Hay, P. J.; Wadt, W. R. Ab initio effective core potentials for molecular calculations. Potentials for K to Au including the outermost core orbitals. *J. Chem. Phys.* **1985**, *82*, 299–310.

(33) Hay, P. J.; Wadt, W. R. Ab initio effective core potentials for molecular calculations. Potentials for the transition metal atoms Sc to Hg. *J. Chem. Phys.* **1985**, *82*, 270–283.

(34) Zhao, Y.; Truhlar, D. G. A new local density functional for main-group thermochemistry, transition metal bonding, thermochemical kinetics, and noncovalent interactions. *J. Chem. Phys.* **2006**, *125*, 194101.

Cartesian coordinates

1

Bi	-0.35399600	-0.00214600	-1.16693200
Mn	1.97858700	-0.34020600	0.46896300
O	1.71296600	2.53490800	1.26605500
O	1.99968500	-3.13699600	-0.57829700
O	-0.01155200	-1.02350600	2.59701300
O	4.37472800	-0.74808400	2.14154300
O	3.38418400	0.53705300	-2.01771400
C	1.79082200	1.43540900	0.94469700
C	3.44029500	-0.58528700	1.48831800
C	-1.80865700	-1.37391300	-0.12573300
C	1.97386000	-2.06092100	-0.16797700
C	0.73856300	-0.77340200	1.76207400
C	2.85032000	0.19578000	-1.05678200
C	-3.10184800	-0.91234200	0.15682600
C	-1.50837900	-2.71771100	0.13464800
H	-0.51655400	-3.12407100	-0.08514400
C	-2.46503500	-3.57088400	0.68498000
H	-2.20764400	-4.61344400	0.88817200
C	-4.06071600	-1.76578600	0.70573300
H	-5.06187300	-1.38535500	0.92419500
C	-1.09615900	1.85027400	-0.12545500
C	-1.60623600	1.84720100	1.17992200
C	-3.74322100	-3.09559200	0.97559200
H	-4.49176600	-3.76243500	1.40977500
C	-0.96211700	3.07445700	-0.79281300
H	-0.56339100	3.11142900	-1.81305200
C	-1.97199600	3.04122800	1.80082700
H	-2.36537400	3.02319400	2.82048100
C	-1.32732900	4.27045000	-0.17168900
H	-1.21565800	5.21735200	-0.70595100
C	-1.83052100	4.25520600	1.12751900
H	-2.11282100	5.19003900	1.61736300
H	-1.72245900	0.90616500	1.72773800
H	-3.37542300	0.12835700	-0.04520400

1_rad

Bi	0.00478800	-1.21881700	-0.00464500
C	-1.65505400	0.28619600	-0.05125700
C	-1.53460900	1.52066800	-0.70956200
C	-2.88781700	-0.03621100	0.53822100
H	-3.01956000	-0.99165400	1.05773200
C	-3.96263100	0.85123000	0.48523400
H	-4.91242700	0.58431400	0.95631400
C	-2.60879100	2.40836400	-0.76399100
H	-2.49620500	3.36452700	-1.28153900
C	1.65093300	0.29990000	0.05017700
C	1.50022000	1.55877800	0.65374700
C	-3.82469900	2.07729600	-0.16496800
H	-4.66532100	2.77396500	-0.20726000
C	2.90821700	-0.04021200	-0.47489800
H	3.06427100	-1.01504600	-0.94997100
C	2.56930600	2.45193700	0.71802400
H	2.43309700	3.42700700	1.19315400
C	3.97785300	0.85253400	-0.41168500
H	4.94712900	0.57156600	-0.83157900
C	3.81010900	2.10246100	0.18406700
H	4.64707900	2.80306500	0.23519100
H	0.53708400	1.84944900	1.08537600
H	-0.59077800	1.79700400	-1.19057400

2

Bi	0.02769400	0.02222600	-1.04650800
C	-1.10850700	-1.56217500	0.17352500
C	-0.55378700	-2.66367800	0.82982900
H	0.52204300	-2.84275900	0.81736500
C	-1.35706100	-3.56798000	1.53047200
H	-0.89047400	-4.42004200	2.03030100
C	-2.73675300	-3.38996800	1.60284200
H	-3.35817100	-4.10040800	2.15163600
C	-2.49856700	-1.41611300	0.26834100
C	-3.31989100	-2.29530700	0.96941500
H	-4.39674200	-2.11386900	1.00051200
C	-2.54809200	1.37006800	0.25979000
C	-3.39985500	2.23054500	0.94704700

H	-4.46815800	2.00610300	0.99121500
C	-2.85825700	3.36521700	1.54707900
H	-3.50476200	4.06043200	2.08637500
C	-1.49039100	3.60823100	1.44548400
H	-1.05915800	4.49858000	1.90913900
C	-0.65439100	2.72348200	0.75710200
H	0.40831000	2.96083500	0.70322100
C	-1.16671000	1.57520600	0.14935300
Mn	2.58035900	-0.00314300	0.33080700
C	1.40798300	-0.15402200	1.74592500
O	0.71581600	-0.21869200	2.66402500
C	2.59418800	-1.80095400	-0.04514800
O	2.69009800	-2.91660800	-0.31570200
C	4.12540800	-0.16869500	1.24120500
O	5.10971800	-0.27873400	1.82800400
C	3.20900900	0.26953000	-1.36933300
O	3.58285300	0.44847900	-2.44403000
C	2.63929500	1.81842500	0.53862800
O	2.77467200	2.95560600	0.66168700
S	-3.25020500	-0.03945700	-0.57695400
O	-4.69929600	-0.06394600	-0.39451600
O	-2.64373100	-0.03042600	-1.92670100

2_rad

Bi	-0.00002400	-1.61445600	0.21234800
C	1.58763800	-0.09756600	-0.29320800
C	2.75997200	-0.47359300	-0.95939100
H	2.94418100	-1.52591200	-1.19985900
C	3.70930800	0.47928800	-1.33567000
H	4.61893200	0.16146100	-1.85107000
C	3.49921700	1.83110800	-1.06630100
H	4.24163100	2.57419800	-1.36511100
C	1.41040500	1.26698200	-0.02715400
C	2.33840800	2.23352700	-0.40706900
H	2.14446400	3.28231800	-0.17045300
C	-1.41044300	1.26704000	-0.02703300
C	-2.33836100	2.23361100	-0.40707900
H	-2.14438000	3.28240200	-0.17050500
C	-3.49911700	1.83120700	-1.06639500
H	-4.24141400	2.57430900	-1.36540000
C	-3.70925700	0.47938100	-1.33568500
H	-4.61878700	0.16156800	-1.85127400
C	-2.76003800	-0.47352300	-0.95924300
H	-2.94427800	-1.52585400	-1.19961700
C	-1.58773200	-0.09750800	-0.29303400
S	0.00002300	1.78546800	0.94510400
O	0.00005100	3.24703700	1.03310000
O	0.00010600	0.94898800	2.15319000

3

Bi	0.20864500	-0.00855700	-1.28281900
C	1.33398700	1.64708200	-0.26269100
C	0.75419700	2.91963100	-0.17648400
H	-0.18980700	3.13344100	-0.68625400
C	1.35565600	3.94252500	0.55368200
H	0.88335600	4.92619500	0.60916600
C	2.55627100	3.69460400	1.21497100
H	3.03372200	4.47841500	1.80746700
C	2.57219300	1.40839100	0.36086600
C	3.15664800	2.44388100	1.10542600
H	4.11292600	2.25993800	1.60486600
C	2.69333400	-1.20929600	0.35680600
C	3.39842500	-2.21156100	1.03965700
H	4.35149900	-1.95724500	1.51378800
C	2.92464900	-3.51881600	1.11086200
H	3.49677200	-4.27666800	1.65105100
C	1.72931800	-3.85593300	0.48019500
H	1.35338900	-4.88123400	0.51189200
C	1.00776100	-2.86682400	-0.18406200
H	0.06330300	-3.14333300	-0.66617300
C	1.46002100	-1.54220000	-0.23361600
Mn	-2.04148900	-0.05390300	0.54811000
C	-0.77105000	0.60571200	1.71803000
O	0.00289000	0.96476700	2.48730400
C	-2.38153500	1.60474100	-0.16495700
O	-2.64811300	2.63153200	-0.61277400
C	-3.46774000	0.06165300	1.63641800

O	-4.38331000	0.14331000	2.33054200
C	-2.86236900	-0.83512400	-0.89464600
O	-3.36153900	-1.33702300	-1.80334900
C	-1.62575100	-1.73151300	1.18393200
O	-1.42966900	-2.77142000	1.63105600
C	3.35792900	0.13356400	0.18337500
H	3.78023600	0.15278700	-0.84096500
H	4.24056600	0.17313400	0.83761500

3_rad

Bi	-0.00007400	-1.38640600	0.12192900
C	1.55980000	0.20229100	-0.04305000
C	2.79478700	-0.07583300	-0.64497800
H	3.02162000	-1.09036000	-0.99016200
C	3.74917300	0.92433400	-0.82476500
H	4.70612500	0.69077800	-1.29792400
C	3.47042300	2.22265300	-0.40229600
H	4.20682900	3.01758600	-0.54230500
C	1.28364500	1.51199700	0.39904500
C	2.24789300	2.50764600	0.20465400
H	2.03693200	3.52683800	0.54344000
C	-1.28341500	1.51212200	0.39901500
C	-2.24750200	2.50790900	0.20433600
H	-2.03634800	3.52715500	0.54282200
C	-3.47007900	2.22302100	-0.40256500
H	-4.20628400	3.01808600	-0.54285700
C	-3.74912900	0.92463300	-0.82462300
H	-4.70617600	0.69113000	-1.29764600
C	-2.79495900	-0.07568200	-0.64459100
H	-3.02202400	-1.09026400	-0.98948600
C	-1.55984700	0.20237200	-0.04283500
C	0.00008600	1.84291900	1.11916000
H	0.00010100	1.29135500	2.07998300
H	0.00011100	2.90711900	1.39498000

4

Bi	0.13103100	-0.00006600	-1.01587400
C	1.34030300	1.54291700	0.12072000
C	0.90479100	2.60747600	0.91440500
H	-0.15740400	2.76246800	1.11269500
C	1.81724900	3.50567500	1.47049300
H	1.45406800	4.33521700	2.08165200
C	3.18377000	3.34578100	1.24842900
H	3.89881400	4.04829800	1.68235500
C	2.72160900	1.37687700	-0.06611800
C	3.64169600	2.27190600	0.48910600
H	4.71134900	2.11549000	0.33114300
C	2.72177600	-1.37667500	-0.06611000
C	3.64197400	-2.27159900	0.48909900
H	4.71160700	-2.11504400	0.33114200
C	3.18418200	-3.34555400	1.24839100
H	3.89931400	-4.04799000	1.68230300
C	1.81767900	-3.50563800	1.47043200
H	1.45460000	-4.33525600	2.08155000
C	0.90510700	-2.60754200	0.91436300
H	-0.15707200	-2.76269700	1.11260700
C	1.34049100	-1.54289600	0.12072400
Mn	-2.38052100	-0.00001300	0.33712300
C	-1.22656500	0.00021700	1.77996000
O	-0.54479100	0.00030800	2.70616200
C	-2.35940300	1.83395900	0.22011900
O	-2.42322500	2.97826500	0.11325400
C	-3.91586000	0.00023200	1.27781200
O	-4.89352000	0.00039200	1.88562200
C	-3.08109800	-0.00041500	-1.35884000
O	-3.50368700	-0.00068400	-2.42939400
C	-2.35945500	-1.83403500	0.22096700
O	-2.42332500	-2.97840000	0.11465800
S	3.34131600	0.00013700	-1.03825600

4_rad

Bi	0.00001100	-1.41592700	-0.16827500
C	-1.60026400	0.09947300	0.13333400
C	-2.83655700	-0.32209500	0.64940500
H	-2.98418700	-1.37680900	0.90650000
C	-3.89386700	0.56496100	0.83292300
H	-4.84872700	0.20674000	1.22444200

C	-3.72238300	1.91213300	0.51399900
H	-4.53926200	2.62291200	0.65850600
C	-1.43741000	1.46400200	-0.17231500
C	-2.49929200	2.36078600	0.02578200
H	-2.35473000	3.41966700	-0.20302300
C	1.43736700	1.46402300	-0.17229300
C	2.49919400	2.36086200	0.02596300
H	2.35453400	3.41976600	-0.20266700
C	3.72228200	1.91224000	0.51417700
H	4.53906400	2.62308800	0.65892700
C	3.89389800	0.56502300	0.83282500
H	4.84875200	0.20683400	1.22436800
C	2.83665600	-0.32209700	0.64912700
H	2.98439200	-1.37684100	0.90603400
C	1.60031400	0.09946700	0.13318300
S	-0.00002200	2.17649500	-0.93768200

5

Bi	0.00046700	0.31766100	-1.07236700
C	-1.62227300	-0.89741900	-0.08300100
C	-2.86821200	-0.91741400	-0.71336600
H	-3.06031700	-0.28499900	-1.58792000
C	-3.90218500	-1.73919800	-0.25258200
C	-3.69588400	-2.58760600	0.83251300
H	-4.49949900	-3.23783000	1.18034300
C	-1.44748200	-1.74735500	1.01866700
C	-2.45838200	-2.59418500	1.47091500
H	-2.26770000	-3.24261300	2.32851200
C	1.44968500	-1.74627200	1.01886300
C	2.46099000	-2.59269400	1.47095900
H	2.27058000	-3.24145100	2.32836900
C	3.69852800	-2.58530600	0.83263300
H	4.50245700	-3.23521100	1.18033100
C	3.90444700	-1.73650700	-0.25222600
C	2.87007500	-0.91513300	-0.71285400
H	3.06189200	-0.28245000	-1.58727200
C	1.62409000	-0.89593500	-0.08255700
Mn	-0.00214500	2.66736400	0.48699500
C	-1.30162400	3.20205900	-0.69377500
O	-2.11574600	3.51918700	-1.44275400
C	-1.34976100	1.87262400	1.49376100
O	-2.24839100	1.45713600	2.06973900
C	-0.00380300	4.19705900	1.44030200
O	-0.00485300	5.17575500	2.04409500
C	1.34707300	1.87512900	1.49357000
O	2.24644700	1.46117300	2.06949200
C	1.29605000	3.20545900	-0.69366400
O	2.10931700	3.52494800	-1.44256000
S	0.00100400	-1.64260400	2.06974800
O	0.00136100	-2.80531600	2.95739000
O	0.00049600	-0.28664300	2.63408700
C	5.22668700	-1.68581900	-0.96484200
C	-5.22442800	-1.68934700	-0.96525500
F	-5.75085800	-0.45568300	-0.92227400
F	-5.08741000	-2.00571000	-2.26307900
F	-6.11929700	-2.53022100	-0.43701500
F	5.08987600	-2.00200100	-2.26273400
F	5.75246800	-0.45189000	-0.92160500
F	6.12197900	-2.52633000	-0.43673700

5_rad

Bi	-0.00002200	-1.55913900	-0.83657500
C	-1.58325300	-0.05503200	-0.26502900
C	-2.74882400	-0.45108900	0.39290800
H	-2.94094600	-1.50842400	0.60338800
C	-3.69707000	0.49252700	0.80609700
C	-3.49065700	1.85259000	0.58163800
H	-4.23414300	2.57978700	0.91033800
C	-1.40771500	1.31896900	-0.48515600
C	-2.33177900	2.27193000	-0.06974000
H	-2.14103400	3.32842200	-0.27097100
C	1.40778100	1.31894500	-0.48531700
C	2.33195600	2.27188100	-0.07009100
H	2.14126600	3.32836700	-0.27140900
C	3.49087200	1.85252200	0.58120500
H	4.23446000	2.57969600	0.90972800
C	3.69720200	0.49246800	0.80579300

C	2.74886400	-0.45112400	0.39276300
H	2.94095400	-1.50845600	0.60329000
C	1.58326400	-0.05505200	-0.26511500
S	-0.00001600	1.86785200	-1.44610200
O	-0.00000500	3.32975900	-1.48966400
O	-0.00009800	1.06073300	-2.67271400
C	4.93112200	-0.00154900	1.50692000
C	-4.93111600	-0.00151800	1.50698300
F	-5.61463300	-0.86325700	0.73726000
F	-4.61748100	-0.65869800	2.63550400
F	-5.76277800	0.99201500	1.83741300
F	4.61739100	-0.65650000	2.63671600
F	5.61346900	-0.86524500	0.73836300
F	5.76385900	0.99176700	1.83528500

6

0 1

Bi	0.16270600	0.01651300	-1.27782900
C	-1.62632900	-0.91201400	-0.17027500
C	-1.65355000	-2.14134600	0.47107000
H	-0.78821200	-2.80240700	0.52364500
C	-2.82027900	-2.60543400	1.11009200
C	-3.98220900	-1.82440000	1.11661400
H	-4.89328200	-2.16984500	1.60550500
C	-2.80633500	-0.14930400	-0.14583800
C	-3.96533300	-0.58276700	0.48614800
H	-4.85221200	0.05539300	0.47366600
C	-1.60202500	2.34933000	-0.03425300
C	-2.00549700	3.48624400	0.66854400
H	-3.05615700	3.78524600	0.64699600
C	-1.06235200	4.21766800	1.37076100
H	-1.33231800	5.11587100	1.92787900
C	0.27839300	3.80294600	1.37138800
C	0.66410100	2.65102400	0.66306800
H	1.71018200	2.35308400	0.67896200
C	-0.27532700	1.90739800	-0.05000200
Mn	2.37164700	-1.17079200	0.20160600
C	1.18987700	-0.77473300	1.56019000
O	0.50320900	-0.51860600	2.44892300
C	1.58441400	-2.78021100	-0.21219500
O	1.17908600	-3.81732100	-0.50606200
C	3.63109200	-2.01696600	1.17018600
O	4.43338700	-2.55814100	1.79450100
C	3.12100900	-1.21272600	-1.46962700
O	3.57720000	-1.22227500	-2.52771800
C	3.23475100	0.42510900	0.43934400
O	3.86088500	1.38311600	0.58617300
S	-2.79413400	1.42322800	-0.96481600
O	-4.10958100	2.05356500	-0.87861100
O	-2.14660400	1.16760300	-2.27445400
O	-2.71814100	-3.81417900	1.69589900
O	1.13308300	4.56333000	2.08138900
C	2.48520300	4.17728300	2.14456600
H	2.95799900	4.16499700	1.14933900
H	2.99395300	4.91749700	2.76988100
H	2.60528800	3.18128300	2.60124100
C	-3.84400300	-4.33393600	2.35910000
H	-3.55121200	-5.30952500	2.75969300
H	-4.16202700	-3.68753600	3.19320200
H	-4.69543700	-4.47217700	1.67309200

6_rad

Bi	-0.20224200	-1.58257800	-0.54954500
C	-1.61628200	0.08613400	0.00761100
C	-2.80552800	-0.18836600	0.69180600
H	-3.07717800	-1.22228300	0.92072800
C	-3.65120500	0.85633200	1.09794900
C	-3.29763000	2.18877900	0.83466900
H	-3.97043500	2.98139300	1.16572400
C	-1.29387700	1.42253400	-0.25099200
C	-2.11906900	2.47066300	0.16177700
H	-1.82746600	3.49933100	-0.06289400
C	1.50081200	1.12911500	-0.28970100
C	2.53521400	1.97501700	0.09232100
H	2.45853200	3.04271100	-0.12715000
C	3.65875800	1.45471800	0.73342000
H	4.46944600	2.12341300	1.02406000

C	3.72874300	0.07896000	0.98668000
C	2.66661300	-0.76233000	0.60691300
H	2.76493200	-1.82509400	0.84774400
C	1.54472900	-0.25224400	-0.03731600
S	0.14535800	1.79811700	-1.23256000
O	0.29705000	3.25347400	-1.30897200
O	0.04498100	0.97969000	-2.45050600
O	-4.81639000	0.67355400	1.75458100
O	4.76834600	-0.52675400	1.59760600
C	-5.22732400	-0.63843900	2.04127900
H	-4.51154200	-1.16045700	2.69764200
H	-6.18823500	-0.56192100	2.56036800
H	-5.36637500	-1.23582900	1.12514700
C	5.85619100	0.26499200	2.00358000
H	6.58183000	-0.40973400	2.46902600
H	6.33764500	0.76998700	1.15011100
H	5.55646300	1.02764500	2.74106000

7

Bi	0.34709900	0.20918200	-1.17782400
C	-1.47701400	-0.58337200	-0.11203200
C	-2.72143300	-0.15650500	-0.58009200
H	-2.78979400	0.60854000	-1.36228200
C	-3.91049700	-0.68811900	-0.06947100
C	-3.87432900	-1.68693200	0.90057000
H	-4.80286500	-2.10946000	1.28657800
C	-1.46913800	-1.57679000	0.87759900
C	-2.64471800	-2.13639500	1.37523600
H	-2.58292500	-2.91315700	2.14023200
C	1.27111500	-2.42768500	0.51128400
C	2.01144400	-3.58960800	0.73646800
H	1.71311100	-4.26336800	1.54252000
C	3.11747900	-3.86209200	-0.06007900
H	3.69808500	-4.77101600	0.11886200
C	3.49948300	-2.98813200	-1.08628500
C	2.71523200	-1.84614900	-1.30642800
H	2.99740500	-1.18290800	-2.13408500
C	1.60243800	-1.53750200	-0.51756900
Mn	1.20471200	2.24458100	0.58303000
C	0.03780200	3.28219900	-0.37949200
O	-0.69933700	3.91786900	-0.99417600
C	-0.23159200	1.76216300	1.66310400
O	-1.16221100	1.56322500	2.30080400
C	1.75836900	3.57353700	1.66530200
O	2.11336300	4.42607100	2.35161000
C	2.32559000	0.96791900	1.34227800
O	3.10516400	0.24253800	1.76429500
C	2.49474500	2.50199400	-0.69356400
O	3.29920600	2.64405900	-1.50545900
S	0.03595800	-2.03698900	1.74031800
O	-0.24111300	-3.24902700	2.51270600
O	0.48952700	-0.82001800	2.42858600
C	4.71327100	-3.26041400	-1.91474200
C	-5.21345500	-0.16401100	-0.60256600
F	-5.33382300	1.15438900	-0.38051000
F	-5.30090700	-0.33996800	-1.93155900
F	-6.27067700	-0.76537900	-0.04669100
H	4.69233800	-2.71765400	-2.86828600
H	5.62833900	-2.94880000	-1.38728600
H	4.82707300	-4.33076000	-2.13289000

7_rad

Bi	0.62331400	-1.59952700	-0.50826200
C	-1.01380800	-0.06489300	-0.25678600
C	-2.28023400	-0.42892600	0.20339300
H	-2.52102500	-1.47766800	0.40727900
C	-3.27100700	0.53606200	0.42215200
C	-3.00864400	1.88639500	0.19711100
H	-3.78621300	2.63082300	0.37315000
C	-0.78184400	1.29973200	-0.48288200
C	-1.74939800	2.27332100	-0.25895700
H	-1.51055700	3.32104000	-0.45495800
C	1.99484600	1.26638700	-0.01647100
C	2.85543000	2.21398200	0.53281500
H	2.71935100	3.26898400	0.28377600
C	3.87728500	1.79017200	1.37680900
H	4.56002800	2.52678200	1.80901500

C	4.04633300	0.43158800	1.67877800
C	3.14839500	-0.49250100	1.12224200
H	3.27077200	-1.55028100	1.38316400
C	2.11441200	-0.09977900	0.26705900
S	0.77912000	1.80581600	-1.20530700
O	0.80542900	3.26714100	-1.28349200
O	0.95711600	0.97195600	-2.40172300
C	5.16520600	-0.02005100	2.56198700
C	-4.61265000	0.07686800	0.91675300
F	-5.16833900	-0.80625900	0.07114400
F	-4.50784200	-0.54354300	2.10417400
F	-5.47585100	1.08767800	1.06746300
H	5.32151800	0.66543500	3.40574100
H	4.99072800	-1.02450500	2.96760300
H	6.11638900	-0.05671300	2.00859000

8

Bi	-0.08664400	0.29372700	-1.08041400
C	-1.77866300	-0.78602900	-0.06579500
C	-3.03735400	-0.68559100	-0.66065400
H	-3.17531000	0.00822900	-1.49937800
C	-4.14641500	-1.43579000	-0.23186000
C	-3.94452200	-2.34738600	0.80979400
H	-4.76709900	-2.96720400	1.16949600
C	-1.63104700	-1.69632300	0.98950600
C	-2.69784300	-2.48155900	1.41823900
H	-2.54417300	-3.18534400	2.23925800
C	1.26114000	-1.88474100	0.93821700
C	2.26865300	-2.73593300	1.38637800
H	2.07278900	-3.38945600	2.23944100
C	3.51507800	-2.72239400	0.76328100
H	4.29365700	-3.38470600	1.14467900
C	3.77523000	-1.87016000	-0.31557300
C	2.71978500	-1.06352500	-0.77278400
H	2.90200100	-0.41189100	-1.63683100
C	1.46612200	-1.03986900	-0.16042300
Mn	0.35693700	2.57316400	0.54232600
C	-0.82063300	3.37334700	-0.61205600
O	-1.55953700	3.86695300	-1.34496100
C	-1.10124400	1.96763400	1.52570600
O	-2.05269000	1.67507200	2.09353300
C	0.63057600	4.06189300	1.51583300
O	0.80451200	5.01979100	2.13042200
C	1.56370900	1.56216200	1.53741500
O	2.40296000	1.02201000	2.09967400
C	1.72654300	2.82000000	-0.64686500
O	2.59398000	2.93019800	-1.39871000
S	-0.16419900	-1.73511000	2.00835300
O	-0.22502300	-2.92858400	2.85485900
O	-0.06915500	-0.40630800	2.63362500
C	5.14258100	-1.76863200	-0.98908100
C	-5.50050300	-1.23363500	-0.91084800
C	-5.92818100	0.22959400	-0.74222800
H	-6.02032600	0.50030500	0.31957200
H	-5.21151200	0.92690800	-1.19889800
H	-6.90400900	0.40479100	-1.21917900
C	-6.57993000	-2.12874000	-0.30791000
H	-6.73426800	-1.92704200	0.76202000
H	-7.54034800	-1.95450500	-0.81335200
H	-6.34298800	-3.19676000	-0.42028400
C	-5.37263200	-1.56042400	-2.40364000
H	-4.64044800	-0.91514900	-2.90944000
H	-5.05966400	-2.60295000	-2.56081600
H	-6.33739200	-1.42052400	-2.91363800
C	5.00734600	-2.10571600	-2.47846900
H	4.63152200	-3.12871800	-2.62594200
H	4.32182400	-1.42252100	-2.99945100
H	5.98332800	-2.03255300	-2.98088800
C	5.65683000	-0.33126400	-0.83256300
H	4.98927900	0.40292800	-1.30653100
H	5.75032300	-0.05397700	0.22775400
H	6.64793000	-0.22082300	-1.29740300
C	6.15821800	-2.72116300	-0.36408300
H	7.12986900	-2.62129700	-0.86823000
H	6.32240700	-2.50903000	0.70232200
H	5.85036900	-3.77297200	-0.45645600

8_rad

Bi	0.00000100	-1.48588100	-0.91534500
C	-1.58842500	-0.01929000	-0.27964200
C	-2.75726000	-0.45196200	0.34832100
H	-2.92205600	-1.52708300	0.48610200
C	-3.73704300	0.43983400	0.82208600
C	-3.50367000	1.81062600	0.66113900
H	-4.23300300	2.54034300	1.01582200
C	-1.40711300	1.36293400	-0.42565100
C	-2.34378300	2.27643300	0.04148500
H	-2.16239900	3.34524000	-0.09490400
C	1.40710700	1.36293600	-0.42563900
C	2.34377200	2.27643600	0.04150600
H	2.16238400	3.34524300	-0.09487600
C	3.50365900	1.81062900	0.66115900
H	4.23298900	2.54034700	1.01585000
C	3.73703900	0.43983600	0.82209500
C	2.75725700	-0.45196000	0.34832600
H	2.92205700	-1.52708100	0.48610200
C	1.58842200	-0.01928900	-0.27963500
S	0.00000000	1.97028600	-1.34284400
O	-0.00000100	3.43413800	-1.29263400
O	0.00000600	1.25347300	-2.62615700
C	4.99539600	-0.11310300	1.49232800
C	-4.99539400	-0.11310500	1.49233300
C	-5.73644900	-1.01890400	0.50143600
H	-5.12081800	-1.86963300	0.17665300
H	-6.64769200	-1.43092200	0.96035500
H	-6.03655200	-0.46542900	-0.40019100
C	-5.94308900	0.99870100	1.93508400
H	-6.83666200	0.56702400	2.40802900
H	-5.47823800	1.67032400	2.67144800
H	-6.28667400	1.61097500	1.08867300
C	-4.59210500	-0.92915100	2.72653000
H	-3.94283400	-1.77785800	2.46883700
H	-4.05189700	-0.30981200	3.45736700
H	-5.48204000	-1.33859200	3.22769700
C	5.73644700	-1.01888900	0.50141800
H	6.64767200	-1.43093900	0.96033700
H	5.12080500	-1.86959300	0.17660100
H	6.03657600	-0.46539600	-0.40018400
C	5.94309000	0.99870000	1.93508500
H	5.47824500	1.67031100	2.67146200
H	6.83667200	0.56702300	2.40801500
H	6.28666400	1.61098700	1.08867800
C	4.59212100	-0.92916400	2.72651800
H	4.05191600	-0.30983700	3.45736600
H	3.94285200	-1.77787200	2.46882000
H	5.48206100	-1.33860400	3.22767400

9

Bi	0.01559500	-0.07369100	-1.21980500
Mn	2.24677600	-0.23008800	0.60657400
S	-3.68791500	0.49858400	1.40478200
O	1.19502600	2.13768100	2.10167300
O	2.97189000	-2.57922500	-1.09437400
O	0.52762400	-1.90874000	2.38732900
O	4.67274100	-0.42642500	2.27427700
O	3.33963900	1.65657800	-1.43914600
C	1.57617600	1.23221700	1.50618100
C	3.72436700	-0.34766700	1.62591800
C	-1.33097700	-1.69774100	-0.38615700
C	2.68784900	-1.67539100	-0.43978800
C	1.17656700	-1.28038700	1.67631400
C	2.93631200	0.92007800	-0.65172000
C	-2.71325400	-1.59114000	-0.13623200
C	-0.72576000	-2.96206700	-0.29582000
H	0.34382600	-3.07813000	-0.50462700
C	-3.46392600	-0.29749700	-0.22236800
H	-4.48517700	-0.45827200	-0.59526400
H	-2.99007100	0.42639900	-0.90559100
C	-1.44736900	-4.09593200	0.06342100
H	-0.94637500	-5.06453800	0.12852500
C	-3.42591300	-2.74145800	0.24136800
H	-4.49396000	-2.64599700	0.46026600
C	-0.80332400	1.87595000	-0.42327400
C	-1.53850600	2.11619000	0.75268500

C	-2.80911700	-3.98153200	0.34197600
H	-3.39026500	-4.86007900	0.63100100
C	-1.95882100	1.01844200	1.67844900
H	-1.92659700	1.34761500	2.72698100
H	-1.30863000	0.13104600	1.59918100
C	-0.42441000	2.96572900	-1.22472800
H	0.15708300	2.80043300	-2.14024500
C	-1.89913900	3.43459500	1.06892000
H	-2.48783800	3.61471200	1.97367500
C	-0.76716100	4.27185000	-0.88411000
H	-0.45380700	5.10364700	-1.51933200
C	-1.51596700	4.50455400	0.26926300
H	-1.79645500	5.52268400	0.54850600

9_rad
0 2

Bi	-0.00066000	-1.48750300	0.00114300
S	0.00133500	3.08752900	0.00363800
C	-1.79144500	-0.12115600	-0.03672000
C	-1.90187800	1.17050700	-0.59344400
C	-2.94416300	-0.72285400	0.50179900
H	-2.88411400	-1.72821300	0.93542400
C	-0.73073700	1.89894600	-1.17467200
H	-1.02906300	2.51438800	-2.03518900
H	0.05772800	1.21393300	-1.52668100
C	-4.17423200	-0.07274700	0.50754500
H	-5.05224100	-0.56450800	0.93342900
C	-3.14662900	1.81831800	-0.57048100
H	-3.22027800	2.82773300	-0.98717100
C	1.79143800	-0.12274200	0.03503300
C	1.90389100	1.16798900	0.59342300
C	-4.27443900	1.21016600	-0.03215800
H	-5.23289900	1.73445900	-0.03631800
C	0.73428600	1.89588200	1.17835000
H	1.03435900	2.50939200	2.03963600
H	-0.05410500	1.21060300	1.53000500
C	2.94231100	-0.72404200	-0.50774800
H	2.88057900	-1.72868700	-0.94280000
C	3.14877200	1.81544200	0.56795200
H	3.22396900	2.82428400	0.98584700
C	4.17255500	-0.07429100	-0.51611800
H	5.04901000	-0.56547200	-0.94557900
C	4.27479200	1.20770300	0.02538300
H	5.23333100	1.73164400	0.02749300

10

Bi	-0.02047900	-0.04874700	-0.98723200
C	0.95766400	1.64632300	0.19337900
C	0.34243900	2.77030600	0.74926800
H	-0.73432100	2.92053100	0.66329500
C	1.08623000	3.73011800	1.44141400
H	0.57491400	4.59752500	1.86523000
C	2.46289000	3.58884100	1.60315000
H	3.03605700	4.34227600	2.14693800
C	2.34112800	1.54614000	0.36907700
C	3.10642000	2.47657800	1.06612000
H	4.18317600	2.32211700	1.16726200
C	2.47426300	-1.24027200	0.61177100
C	3.29308300	-2.02849000	1.41500200
H	4.34911900	-1.76995900	1.52405900
C	2.73144100	-3.13567600	2.04820700
H	3.34882700	-3.77597200	2.68173100
C	1.38094200	-3.42300900	1.85927800
H	0.93643500	-4.29360800	2.34737200
C	0.57997400	-2.61101400	1.04978800
H	-0.46942700	-2.88166500	0.92972600
C	1.11364100	-1.49281800	0.40459500
Mn	-2.70356100	-0.02377100	0.27467800
C	-1.59671300	0.25312900	1.71838500
O	-0.94454000	0.39848600	2.65876400
C	-2.73394400	1.73987300	-0.22752700
O	-2.83333600	2.83389400	-0.57977500
C	-4.30350700	0.16051300	1.07577500
O	-5.32703600	0.28291200	1.59092800
C	-3.17821900	-0.43685900	-1.44273900
O	-3.45189400	-0.70568400	-2.53100800
C	-2.72388900	-1.82415700	0.60924300

O	-2.82858100	-2.95561500	0.80723500
S	3.18415400	0.13718500	-0.30109700
O	4.63015800	0.18768700	-0.03210800
N	2.57246200	0.08654700	-1.74204500
C	2.98170800	-1.04066800	-2.56416100
H	2.75263100	-2.02990500	-2.12040100
H	2.44695400	-0.97617900	-3.52079700
H	4.05926100	-1.02221100	-2.79168900

10_rad

Bi	0.13191300	-1.61114900	0.14917400
C	1.67185400	-0.04088400	-0.35118600
C	2.88039100	-0.31373300	-1.00058200
H	3.14417800	-1.34366400	-1.26188500
C	3.76231500	0.71678300	-1.33486400
H	4.70266400	0.48015000	-1.83901400
C	3.44876400	2.04443900	-1.04220700
H	4.13997200	2.84585700	-1.31123100
C	1.39970200	1.29896600	-0.05743500
C	2.24968000	2.34699700	-0.39912200
H	1.97014800	3.37197000	-0.14402900
C	-1.41225000	1.16259700	-0.29438700
C	-2.34858200	2.08817200	-0.74760700
H	-2.22983600	3.14258200	-0.48641000
C	-3.42034800	1.63241500	-1.51438200
H	-4.17117200	2.33759200	-1.87762500
C	-3.53162300	0.27303300	-1.80977400
H	-4.37214100	-0.08496300	-2.40970000
C	-2.57558400	-0.63606100	-1.35021800
H	-2.68289500	-1.69424900	-1.61077400
C	-1.49375000	-0.20717500	-0.57228000
S	-0.09581700	1.69773400	0.81691600
O	-0.22607600	3.15697300	0.99123800
N	-0.02477400	0.68903700	2.00180000
C	-1.19637800	0.59521300	2.84835500
H	-2.12562400	0.33307400	2.30361100
H	-1.01811600	-0.19549200	3.58977100
H	-1.39599400	1.52518900	3.40680800

11

Bi	0.06755700	-0.32229800	-1.22940500
Mn	-0.22737600	1.94898500	0.52071200
O	-2.36906100	0.55508300	2.07601800
O	1.99304000	2.98675600	-1.19076000
O	1.72188500	0.61651900	2.36153800
O	-0.46553400	4.43248700	2.09426100
O	-2.26097600	2.62782500	-1.56251500
C	-1.54992300	1.06692200	1.45482500
C	-0.37399800	3.45973400	1.48423900
C	2.04205200	-1.04728400	-0.29493100
C	1.13694400	2.58586400	-0.53309000
C	0.99052700	1.11500500	1.62860600
C	-1.46918500	2.38219200	-0.76315100
C	2.06867100	-2.17743700	0.55060100
C	3.22764800	-0.35267700	-0.54566600
H	3.23924400	0.52089000	-1.20703900
C	0.73253800	-2.79429000	0.63418800
C	4.41957500	-0.75268300	0.06546900
H	5.34072700	-0.19676800	-0.12583600
C	3.26055400	-2.57493500	1.15519200
H	3.27080700	-3.44872900	1.81161600
C	-1.68590500	-1.48912900	-0.29311900
C	-1.60686300	-2.26711000	0.88828700
C	4.43240000	-1.85272900	0.92052000
H	5.36374500	-2.15787900	1.40309700
C	-0.25348300	-2.39232800	1.45044800
C	-2.93626100	-1.26260400	-0.87909300
H	-3.02397800	-0.65633900	-1.78903600
C	-2.78038100	-2.76924400	1.46368900
H	-2.71688900	-3.35554000	2.38437100
C	-4.09643000	-1.78849700	-0.30776900
H	-5.06658700	-1.60184200	-0.77493600
C	-4.01688400	-2.53871500	0.86549600
H	-4.92474400	-2.94472200	1.31738400
H	0.41615500	-3.29379700	-0.29470300
H	-0.02568900	-1.88887700	2.40112400

11_rad

Bi	-0.00002800	-1.29262700	-0.27895700
C	1.64169000	0.14510900	0.19291900
C	1.70072400	1.47033000	-0.29909800
C	2.71738900	-0.35586400	0.94032300
H	2.68326300	-1.38386700	1.31819900
C	0.67763700	2.14264500	-1.10115000
C	3.84358600	0.41698200	1.21502200
H	4.66654600	0.00052200	1.80097500
C	2.86569800	2.22339900	-0.03804200
H	2.92921600	3.24118100	-0.43463900
C	-1.64153200	0.14524700	0.19317800
C	-1.70057400	1.47045600	-0.29889500
C	3.91607900	1.71736400	0.71663700
H	4.79665900	2.33488900	0.90794700
C	-0.67754800	2.14272500	-1.10100800
C	-2.71730200	-0.35577200	0.94046100
H	-2.68321700	-1.38383500	1.31820600
C	-2.86566500	2.22344100	-0.03804200
H	-2.92918900	3.24117800	-0.43470200
C	-3.84364500	0.41699100	1.21491700
H	-4.66664300	0.00051700	1.80080000
C	-3.91614700	1.71734400	0.71646700
H	-4.79673700	2.33484400	0.90776300
H	1.11127100	2.89005500	-1.77676400
H	-1.11121400	2.89020100	-1.77652300

12

Bi	0.06127000	0.00000300	-1.33135700
Mn	-1.97937300	0.00000100	0.66705500
O	-1.74818100	-2.97324500	0.45186200
O	-1.74868900	2.97323000	0.45096000
O	0.31253400	0.00039400	2.59698000
O	-4.06726800	0.00007800	2.75105500
O	-3.87095100	-0.00050500	-1.64599000
C	-1.83069400	-1.82850600	0.53452600
C	-3.25233600	0.00004900	1.93738100
C	1.44866400	1.40952200	-0.29715400
C	-1.83100600	1.82850100	0.53401800
C	-0.55343400	0.00025300	1.84364100
C	-3.14745700	-0.00030000	-0.75125700
C	2.47199200	0.73748000	0.41216800
C	1.41518200	2.80414600	-0.33605800
H	0.63173200	3.32721700	-0.89232400
C	2.37803400	3.55327400	0.34154600
H	2.34245100	4.64486500	0.31325900
C	3.43268500	1.50920800	1.08476700
H	4.23280800	1.01931800	1.64464200
C	1.44867500	-1.40951200	-0.29717400
C	2.47199500	-0.73747500	0.41215900
C	3.38437000	2.89940600	1.05268300
H	4.14141500	3.47844300	1.58650400
C	1.41521600	-2.80413600	-0.33612500
H	0.63177300	-3.32720000	-0.89240200
C	3.43269400	-1.50921000	1.08473500
H	4.23280700	-1.01932600	1.64462400
C	2.37808000	-3.55327000	0.34144900
H	2.34251700	-4.64486200	0.31312500
C	3.38440100	-2.89940800	1.05260700
H	4.14145200	-3.47845100	1.58640700

12_rad

Bi	0.00002400	-1.44711000	-0.00001800
C	-1.40281700	0.26744200	-0.00006600
C	-0.73699000	1.51976000	-0.00009600
C	-2.79971300	0.21637100	0.00003000
H	-3.31773600	-0.74772300	0.00003900
C	-3.55353400	1.39003500	0.00013600
H	-4.64518000	1.34175200	0.00023500
C	-1.51278200	2.68819900	-0.00000800
H	-1.02719800	3.66729200	-0.00003900
C	1.40280900	0.26748800	0.00001900
C	0.73693900	1.51978600	-0.00012100
C	-2.90437200	2.62530800	0.00012300
H	-3.48682300	3.54961000	0.00021600
C	2.79970900	0.21645900	0.00018700
H	3.31776700	-0.74761900	0.00034000

C	1.51268700	2.68825100	-0.00021700
H	1.02704700	3.66734900	-0.00042700
C	3.55349400	1.39014300	0.00019700
H	4.64514900	1.34188300	0.00041400
C	2.90428200	2.62540100	-0.00004900
H	3.48669700	3.54972100	-0.00009700

13

Mn	1.65090300	-0.00000500	0.23724900
O	0.24898200	-0.00010900	2.88794000
O	2.52357400	0.00022300	-2.62083200
O	1.43971200	-2.97935100	0.12599800
O	4.36387900	-0.00012000	1.39141500
O	1.43977500	2.97936300	0.12643000
C	0.75922900	-0.00008100	1.86033800
C	3.30423200	-0.00007300	0.94024700
C	2.19250500	0.00012800	-1.51921400
C	1.50018300	-1.83101400	0.17155700
C	1.50021500	1.83101700	0.17181400
C	-1.91029700	1.43927400	0.61009500
C	-2.58497100	0.72621700	1.54379700
C	-2.58496900	-0.72617200	1.54381400
C	-1.91028900	-1.43925400	0.61013100
H	-3.16494100	1.24747300	2.31894000
H	-3.16493100	-1.24741000	2.31897400
Bi	-0.98670000	-0.00000400	-0.84718400
C	-1.92833500	-2.92179100	0.50340300
H	-0.92656800	-3.37347700	0.56259300
H	-2.53587400	-3.37093100	1.30452500
H	-2.35195300	-3.26302200	-0.45505100
C	-1.92835800	2.92180500	0.50331000
H	-2.53590200	3.37097200	1.30441200
H	-0.92659000	3.37348500	0.56249900
H	-2.35198500	3.26300400	-0.45516500

13_rad

C	-1.43227500	0.99083000	0.00000000
C	-0.72784800	2.15023000	0.00000200
C	0.72784600	2.15023000	0.00000300
C	1.43227300	0.99082800	0.00000100
H	-1.25297800	3.11657000	-0.00000300
H	1.25297600	3.11657200	-0.00000400
Bi	0.00000000	-0.72082900	0.00000000
C	2.91577300	0.89323100	-0.00000200
H	3.30144800	0.35142700	-0.87874500
H	3.38622600	1.88915300	-0.00006500
H	3.30144800	0.35153600	0.87880400
C	-2.91577300	0.89322900	-0.00000200
H	-3.30145100	0.35164200	0.87889700
H	-3.38623100	1.88917100	-0.00020900
H	-3.30144000	0.35128500	-0.87868900

14

Bi	0.80281700	-0.00052600	-0.90345200
Mn	-1.75860900	-0.01260800	0.18472600
C	-2.30555000	0.39912000	-1.52276200
O	-2.64456600	0.65831100	-2.58972400
C	-1.74944700	-1.80381900	-0.26303500
O	-1.77653000	-2.91150600	-0.56189500
C	-3.42987500	-0.06324600	0.86277800
O	-4.49609800	-0.09742500	1.29055500
C	-0.94894800	-0.40065800	1.80748300
O	-0.50195000	-0.63972800	2.83409400
C	-1.54145300	1.78982800	0.53066100
O	-1.45387900	2.91234200	0.74774400
C	1.73577800	-1.60919100	0.51497100
C	1.69075300	1.64394500	0.48446200
F	1.46170600	2.86062300	-0.04423800
F	1.19674600	1.66232900	1.73250800
F	3.01870600	1.49633300	0.58446000
F	0.87154500	-2.59475800	0.83129900
F	2.78299500	-2.19155400	-0.09156200
F	2.18030400	-1.09441100	1.66696100

14_rad

Bi	-0.00001100	0.88613800	-0.00000200
C	-1.59036400	-0.82139300	0.00120800

C	1.59037900	-0.82137100	-0.00120900
F	1.72223500	-1.38809800	-1.20770600
F	2.80114700	-0.35607700	0.35272000
F	1.26867100	-1.79426900	0.86171100
F	-2.80138700	-0.35593900	-0.35164600
F	-1.26917600	-1.79376400	-0.86250600
F	-1.72139600	-1.38884000	1.20745100

15

Mn	1.45052500	-0.00058200	0.00001300
C	1.30457800	1.26007100	-1.34493000
O	1.23299400	2.04441100	-2.18125800
C	1.30354300	1.34466700	1.26014000
O	1.23078200	2.18137000	2.04408000
C	3.24269600	-0.00074900	0.00044100
O	4.39397800	-0.00038800	0.00067900
C	1.30295800	-1.26092800	1.34531000
O	1.23023100	-2.04477700	2.18188600
C	1.30304500	-1.34575700	-1.26041100
O	1.23014200	-2.18273300	-2.04390600
Mn	-1.45104700	0.00035600	-0.00008500
C	-1.30280000	-0.05858600	-1.84235900
O	-1.22920300	-0.09488600	-2.98828100
C	-1.30273400	1.84263500	-0.05916300
O	-1.22897400	2.98860400	-0.09528100
C	-3.24329100	0.00070800	-0.00088500
O	-4.39455900	0.00064500	-0.00147400
C	-1.30410200	0.05961200	1.84240500
O	-1.23107100	0.09661200	2.98815200
C	-1.30456500	-1.84203400	0.05916800
O	-1.23218600	-2.98788000	0.09584100

15_rad

Mn	-0.00033100	-0.00081400	-0.18776600
C	1.72240400	-0.63610200	-0.40781900
O	2.79370300	-1.03008500	-0.54071000
C	0.63568300	1.72091100	-0.41177400
O	1.03058600	2.79144400	-0.54731500
C	0.00080900	0.00184900	1.61817100
O	0.00209000	0.00557600	2.77017500
C	-1.72305200	0.63551700	-0.40784800
O	-2.79356000	1.03156800	-0.54060900
C	-0.63633700	-1.72392800	-0.40810700
O	-1.03141500	-2.79464300	-0.54174100

$Mn(CO)_5$alkyl

C	1.28442600	0.63598000	0.00032200
H	1.12301200	1.28048600	-0.87793300
H	1.12328700	1.27996400	0.87901800
C	0.29841900	-0.51000000	0.00020000
H	0.45788800	-1.16189400	-0.87817600
H	0.45835500	-1.16247300	0.87807900
C	-1.14763300	-0.03361500	0.00069300
H	-1.33193100	0.60816800	-0.87682700
H	-1.33116000	0.60864000	0.87803500
C	-2.14887000	-1.18829600	0.00154000
H	-1.96523400	-1.83525200	0.87784900
H	-1.96532700	-1.83653900	-0.87384100
C	-3.53528800	-0.76305000	0.00127100
C	-4.69680400	-0.37905100	0.00096000
Si	-6.44633500	0.19076200	-0.00021200
C	-6.49225000	1.98988200	-0.52710500
H	-5.91161100	2.62464700	0.15745000
H	-6.07404100	2.12494500	-1.53483300
H	-7.52350100	2.37347300	-0.54130400
C	-7.42410700	-0.86092200	-1.20592800
H	-7.39811700	-1.92373700	-0.92596800
H	-8.47961800	-0.55148700	-1.23719500
H	-7.02420100	-0.78022300	-2.22680100
C	-7.14439800	0.00700500	1.73039000
H	-7.11292400	-1.03869800	2.06825500
H	-6.57768600	0.60471100	2.45856400
H	-8.19285100	0.33831900	1.77296600
Mn	3.43378800	0.07389600	-0.00006900
C	3.16584700	0.10140200	-1.81746500
C	5.21731300	-0.26955600	0.00010400
C	3.65581000	1.90506100	0.00343200

C	3.16403300	0.09458100	1.81715000
C	2.98788200	-1.71197800	-0.00364100
O	6.34957000	-0.47753700	0.00025500
O	2.91312000	0.10998000	2.93718600
O	2.91624000	0.12108600	-2.93772400
O	2.70355200	-2.82374100	-0.00585600
O	3.74543900	3.04824000	0.00561700

$Mn(CO)_5I$

Mn	0.82572300	0.00009100	0.00029300
C	0.68006200	0.33155200	1.82605800
O	0.58530200	0.53510100	2.94705500
C	0.68463600	-1.82603800	0.33076000
O	0.59444300	-2.94771000	0.53306500
C	2.61962000	0.00008700	0.00109400
O	3.77007100	0.00000100	0.00170100
C	0.68124500	-0.33118600	-1.82547300
O	0.58737800	-0.53457200	-2.94658100
C	0.68463800	1.82592000	-0.33186000
O	0.59438500	2.94718300	-0.53639900
I	-1.92069600	-0.00008100	-0.00002800

$Bi(C_6H_4)_2$Salkyl

Bi	-1.82403200	-0.21657300	-1.18793300
C	-2.86333100	-1.47989700	0.35076300
C	-2.36603700	-2.68621800	0.85194400
H	-1.39454100	-3.06358500	0.51599300
C	-3.10147000	-3.43724600	1.77037700
H	-2.70367300	-4.38403000	2.14375800
C	-4.34355100	-2.97886100	2.20700600
H	-4.92325000	-3.56139000	2.92682100
C	-4.10493900	-1.01908000	0.81306200
C	-4.84314100	-1.76509500	1.73974700
H	-5.80363800	-1.38586800	2.09668200
C	-3.54369800	1.70288400	0.60904600
C	-3.89421500	2.80024100	1.40489700
H	-4.91512800	2.88972400	1.78373300
C	-2.93605200	3.76198600	1.71803500
H	-3.21243900	4.62037400	2.33471100
C	-1.62573100	3.61641400	1.26408800
H	-0.86979200	4.36231000	1.52146700
C	-1.28020400	2.51626700	0.47766400
H	-0.24632000	2.41588700	0.12971600
C	-2.23622700	1.55987600	0.12360500
S	-4.81821500	0.50907400	0.20079100
C	0.23821800	-0.55734100	-0.29884400
H	0.37654700	-1.64790000	-0.20203300
H	0.20454400	-0.15510100	0.72750600
C	1.37166200	0.05305100	-1.10116000
H	1.38579000	-0.36452800	-2.12502600
H	1.20845100	1.13783400	-1.24448600
C	2.73590200	-0.15478400	-0.46245700
H	2.92386100	-1.23261300	-0.32384500
H	2.74240100	0.27516200	0.55303100
C	3.87443200	0.45612800	-1.27784200
H	3.68762000	1.53543700	-1.42208000
H	3.87009600	0.02878200	-2.29659900
C	5.18624700	0.27247000	-0.68723100
C	6.28147100	0.10454400	-0.16870200
Si	7.93141300	-0.14327600	0.60680800
C	8.71127300	-1.69078300	-0.10976700
H	8.09279400	-2.57917800	0.08233800
H	8.84024000	-1.60942900	-1.19853300
H	9.70324700	-1.87550900	0.32921200
C	9.00368700	1.34992600	0.23721700
H	8.55945200	2.27325700	0.63563500
H	10.00285700	1.24179000	0.68532900
H	9.13877100	1.49058300	-0.84472900
C	7.70398300	-0.33337700	2.45841000
H	7.23494800	0.55756200	2.89985300
H	7.06432100	-1.19454200	2.69902700
H	8.66950900	-0.48540400	2.96384700

$Bi(C_6H_4)_2SI$

Bi	-0.37854600	0.00001100	-1.04533600
C	0.75175400	1.55257200	0.12056300
C	0.23954600	2.61479900	0.86513200

H	-0.84224900	2.74888400	0.96604400
C	1.11015300	3.51163600	1.48792500
H	0.70612500	4.34685900	2.06481000
C	2.49066300	3.34512900	1.37712300
H	3.16796600	4.04849500	1.86679900
C	2.13807000	1.38218300	0.03130100
C	3.01449700	2.27323600	0.65541600
H	4.09389700	2.12312700	0.57977100
C	2.13802500	-1.38223800	0.03130200
C	3.01442500	-2.27329100	0.65545700
H	4.09383100	-2.12318900	0.57985300
C	2.49055600	-3.34517500	1.37715400
H	3.16783700	-4.04853500	1.86687000
C	1.11003600	-3.51167900	1.48789600
H	0.70597500	-4.34689800	2.06476400
C	0.23945800	-2.61483700	0.86506900
H	-0.84234500	-2.74891600	0.96593300
C	0.75170400	-1.55261000	0.12052500
S	2.78090800	-0.00003700	-0.92722300
I	-2.72187900	0.00002800	0.68291400

I-alkyl

C	-2.45446100	0.68612500	-0.00045000
H	-2.34331500	1.32031900	0.88964500
H	-2.34353600	1.31963500	-0.89106200
C	-1.50135900	-0.48029700	-0.00009800
H	-1.68600900	-1.12000200	0.87895800
H	-1.68604400	-1.12057300	-0.87872900
C	-0.05374400	-0.01216700	-0.00025900
H	0.13898200	0.62707800	0.87720100
H	0.13905200	0.62638700	-0.87820400
C	0.93346800	-1.17823700	0.00027800
H	0.74269200	-1.82368400	-0.87539800
H	0.74256600	-1.82294500	0.87647100
C	2.32398700	-0.76626500	0.00021700
C	3.48947100	-0.39510700	0.00018100
Si	5.24777800	0.15161500	0.00002700
C	6.04420400	-0.37852300	1.61219400
H	5.53462400	0.06630800	2.47881800
H	6.01262100	-1.47030900	1.73703800
H	7.09966600	-0.07018400	1.65335500
C	6.12698100	-0.64704700	-1.45031300
H	5.66676700	-0.36206300	-2.40716200
H	7.18458400	-0.34564600	-1.48769600
H	6.09694400	-1.74404200	-1.38491000
C	5.29399900	2.01896200	-0.16220400
H	4.81724200	2.35437100	-1.09431600
H	4.76933000	2.50880500	0.67051600
H	6.32950300	2.39107000	-0.16661100
I	-4.56414800	0.06524900	0.00008300

IR spectra

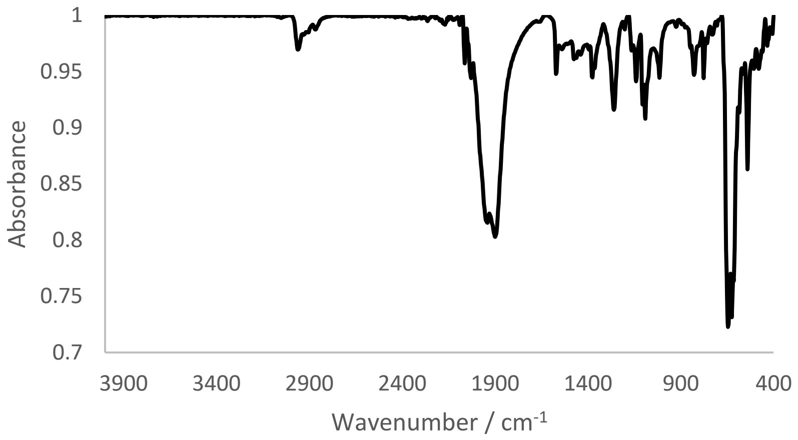

Figure S8. IR spectra of compound **2**.

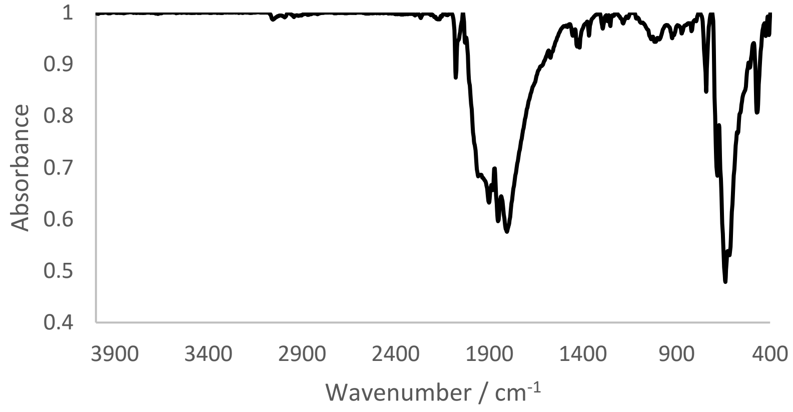

Figure S9. IR spectra of compound **3**.

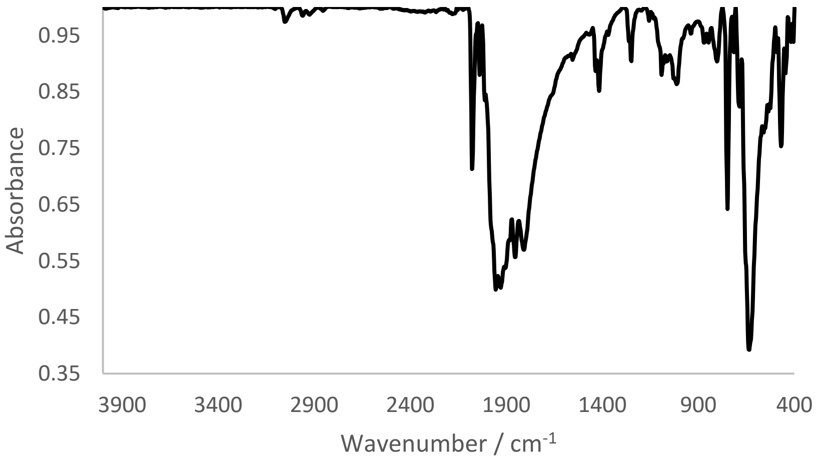

Figure S10. IR spectra of compound **4**.

Copies of NMR spectra

SI-1
^{1}H NMR (300 MHz, $CDCl_3$)

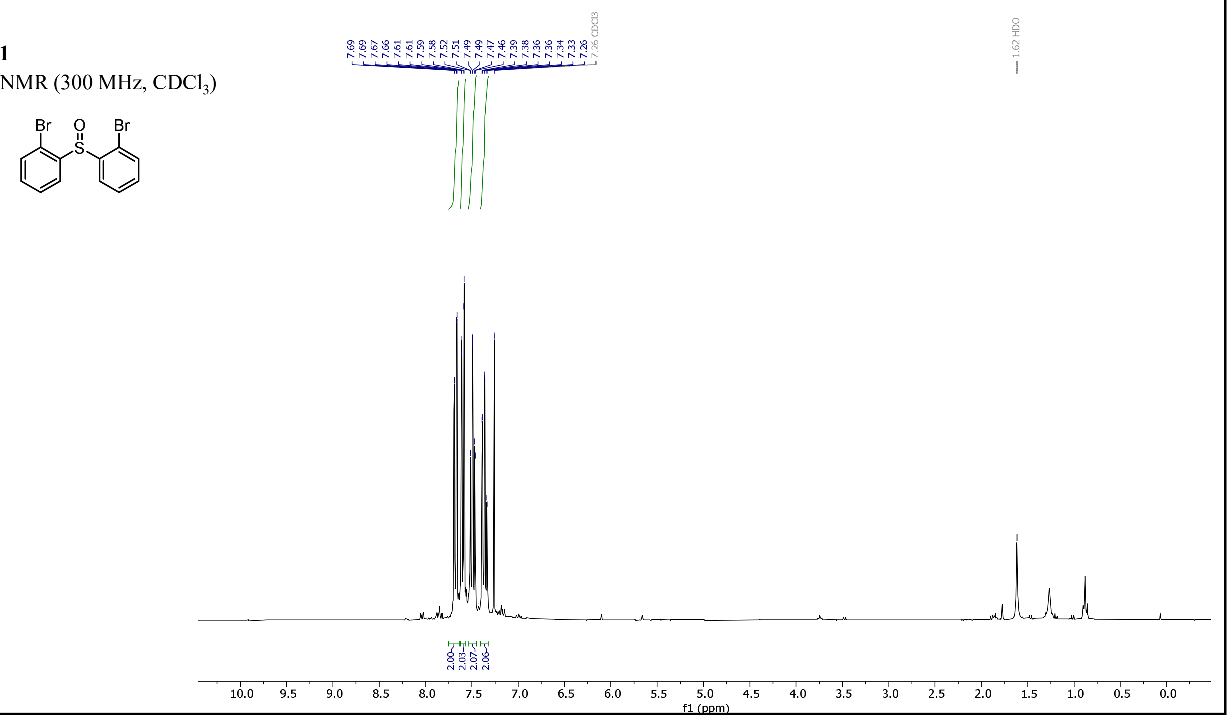

L1
^{1}H NMR (300 MHz, CD_2Cl_2)

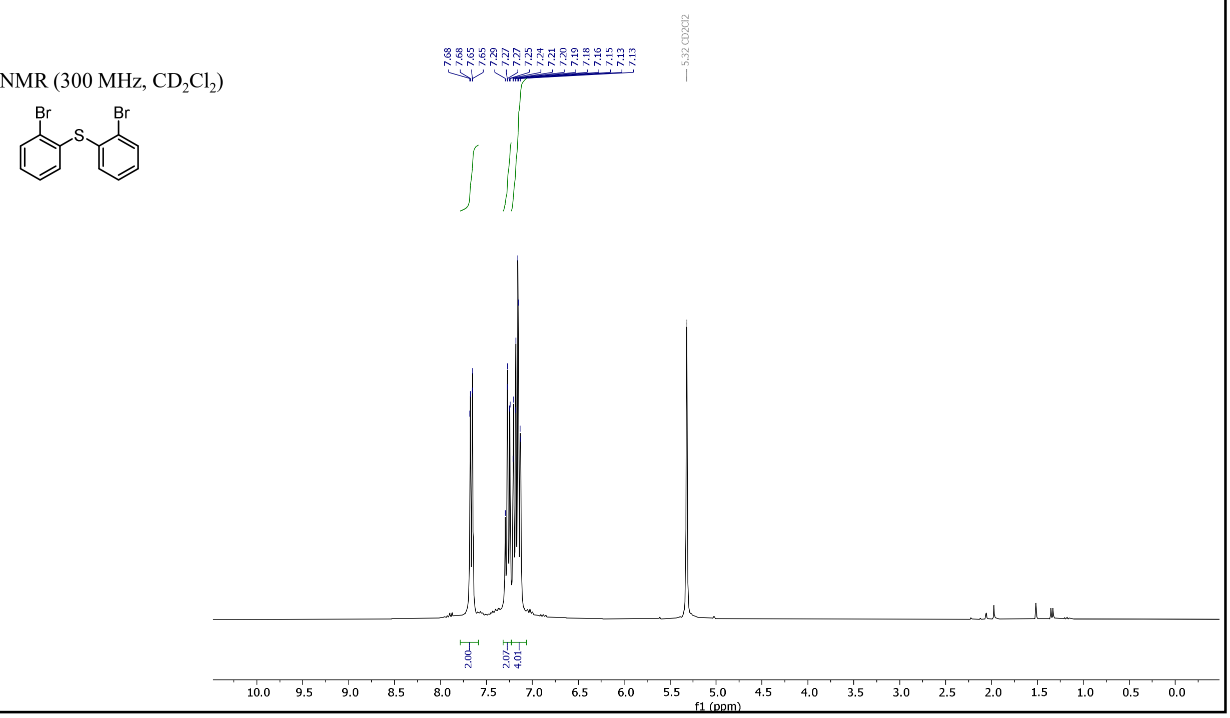

SI-2

^{1}H NMR (300 MHz, $CDCl_3$)

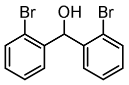

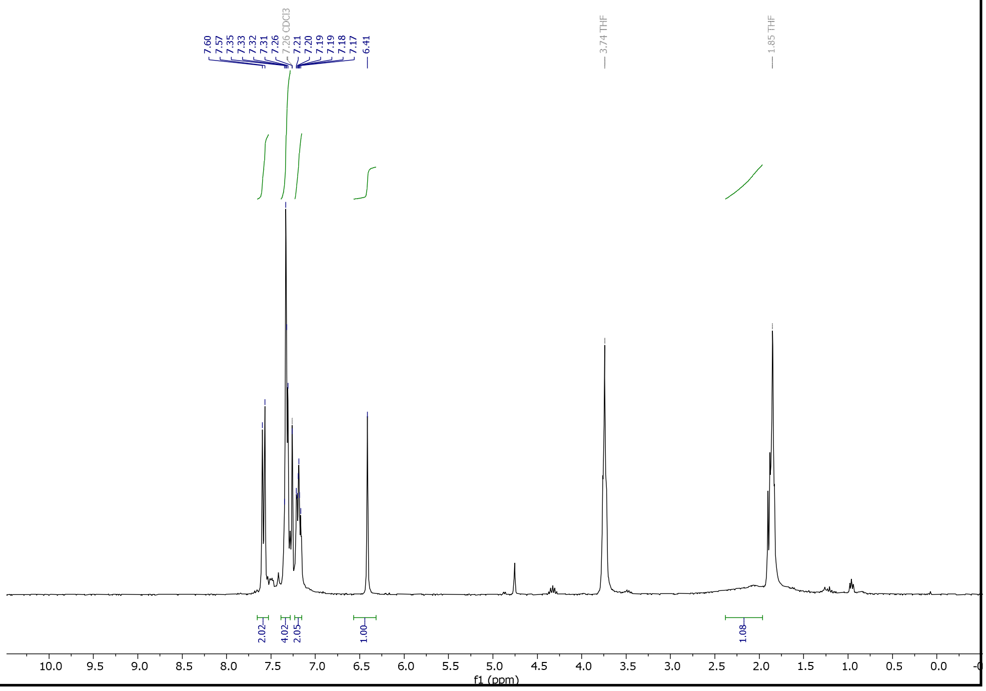

L2

^{1}H NMR (300 MHz, $CDCl_3$)

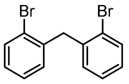

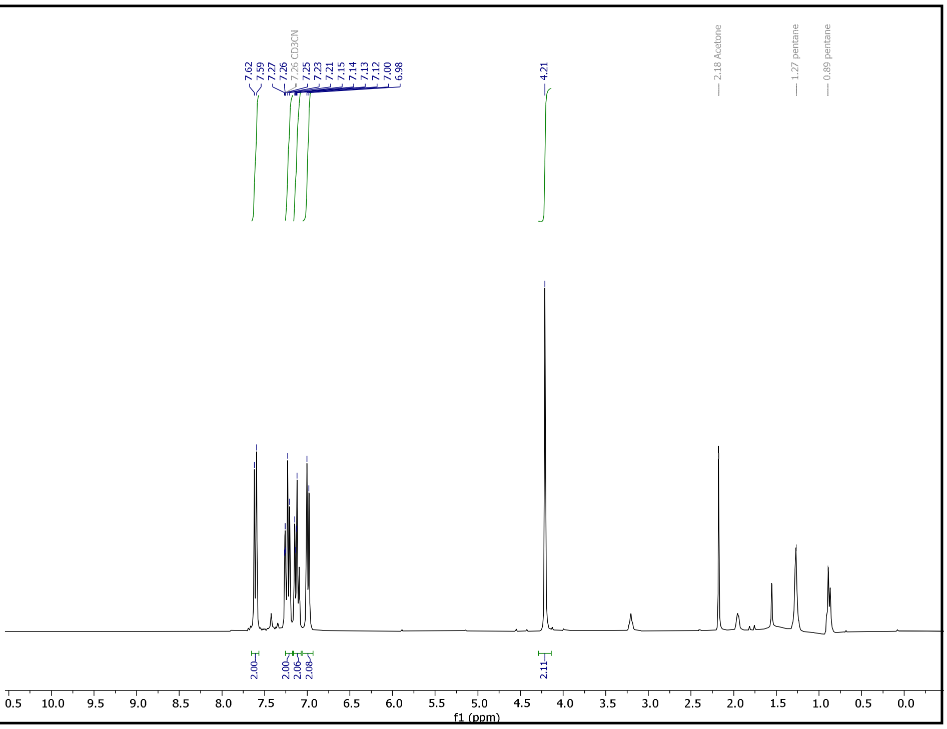

2-Ar

^1H NMR (300 MHz, $CDCl_3$)

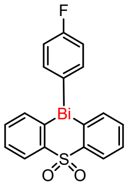

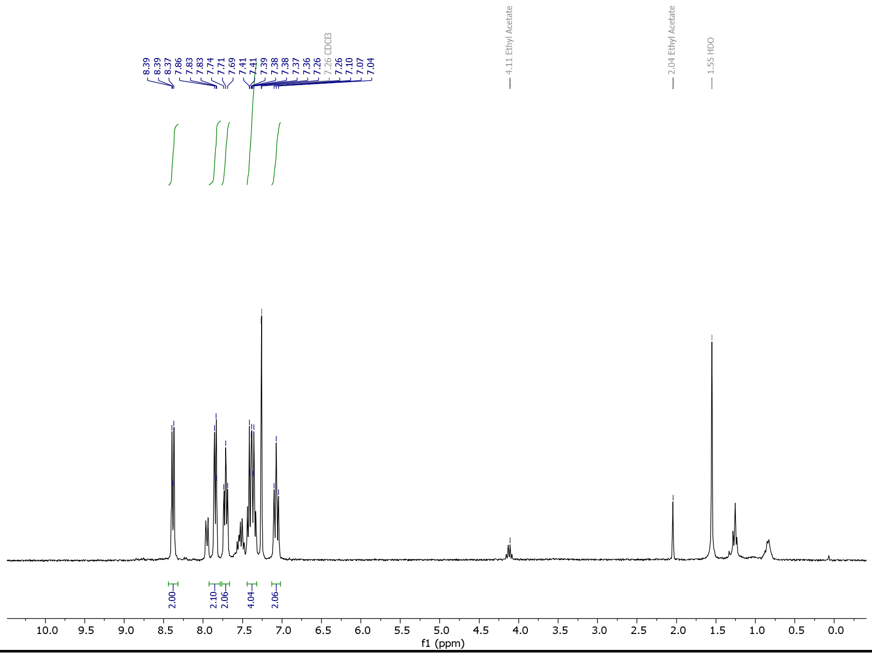

2-I

^1H NMR (300 MHz, DMSO)

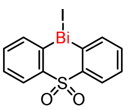

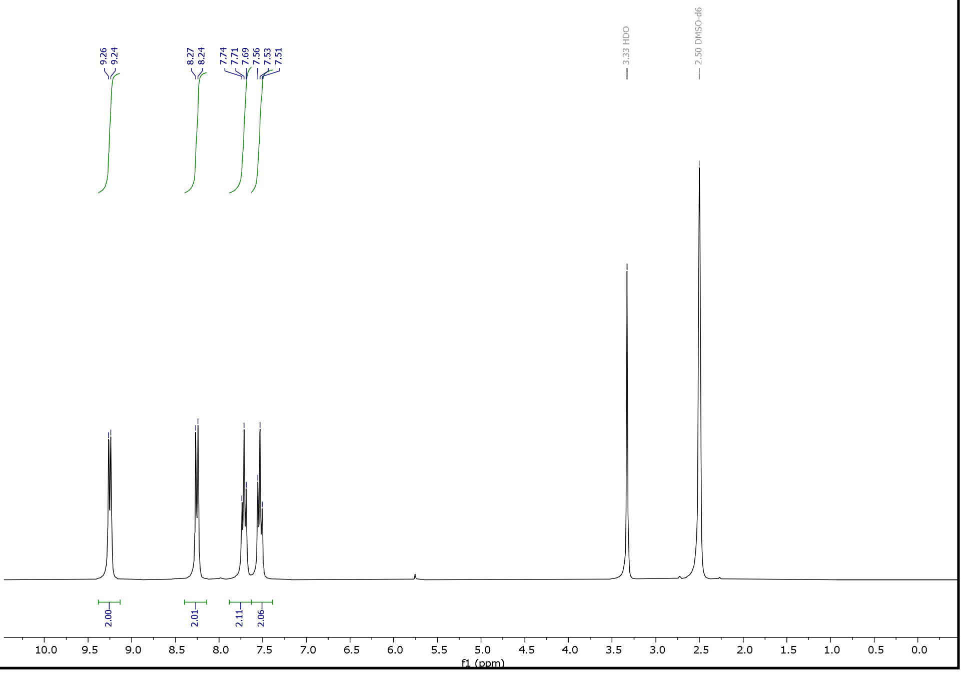

2-I

^{1}H NMR (300 MHz, $CDCl_3$)

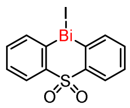

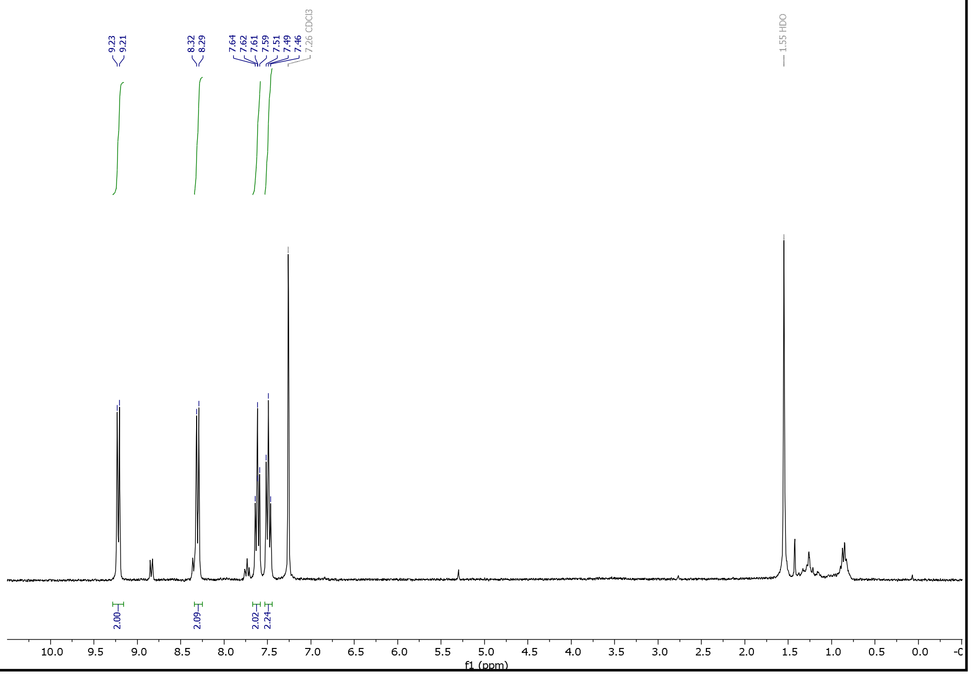

2

^{1}H NMR (300 MHz, CD_3CN)

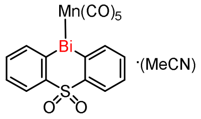

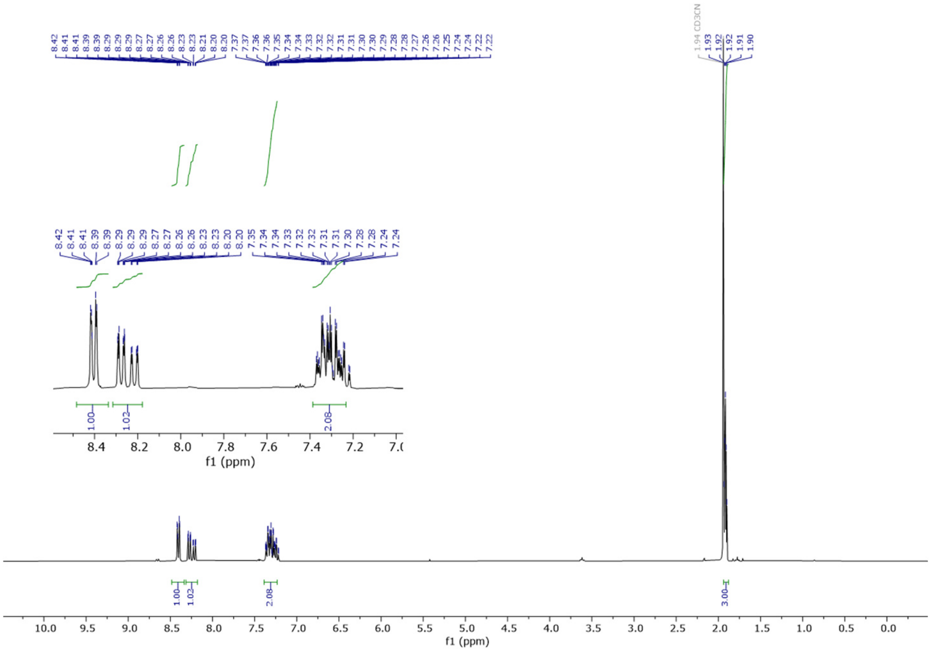

2

^{13}C NMR (75 MHz, CD_3CN)

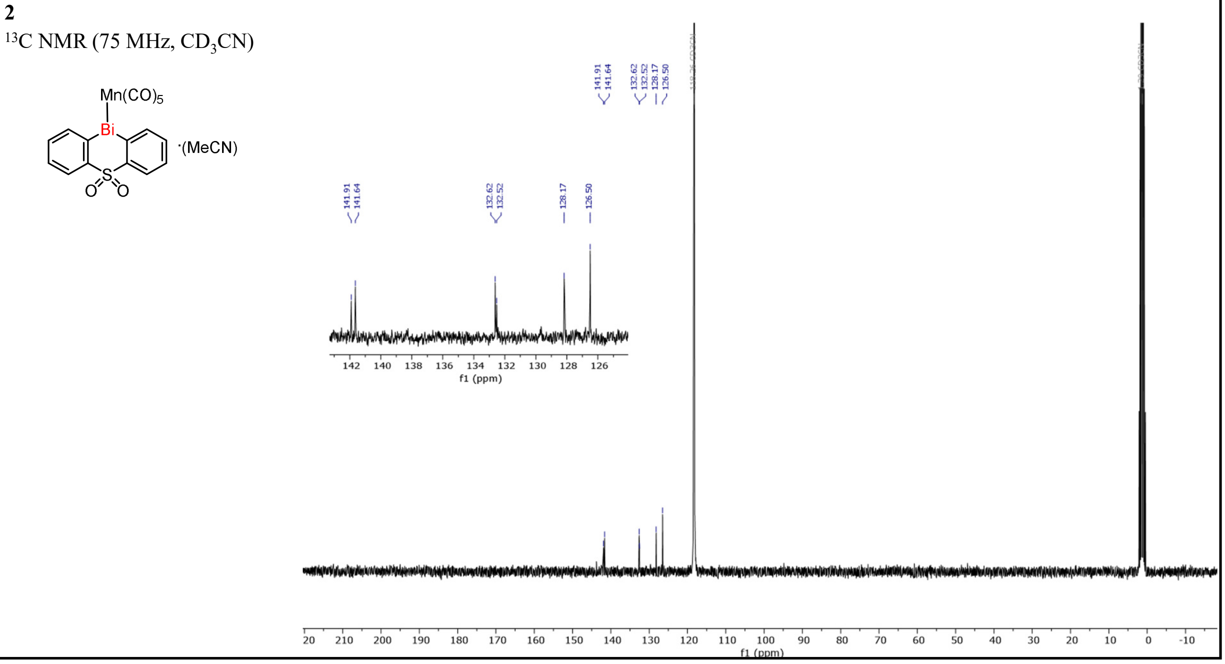

3-Ar

^{1}H NMR (300 MHz, CD_2Cl_2)

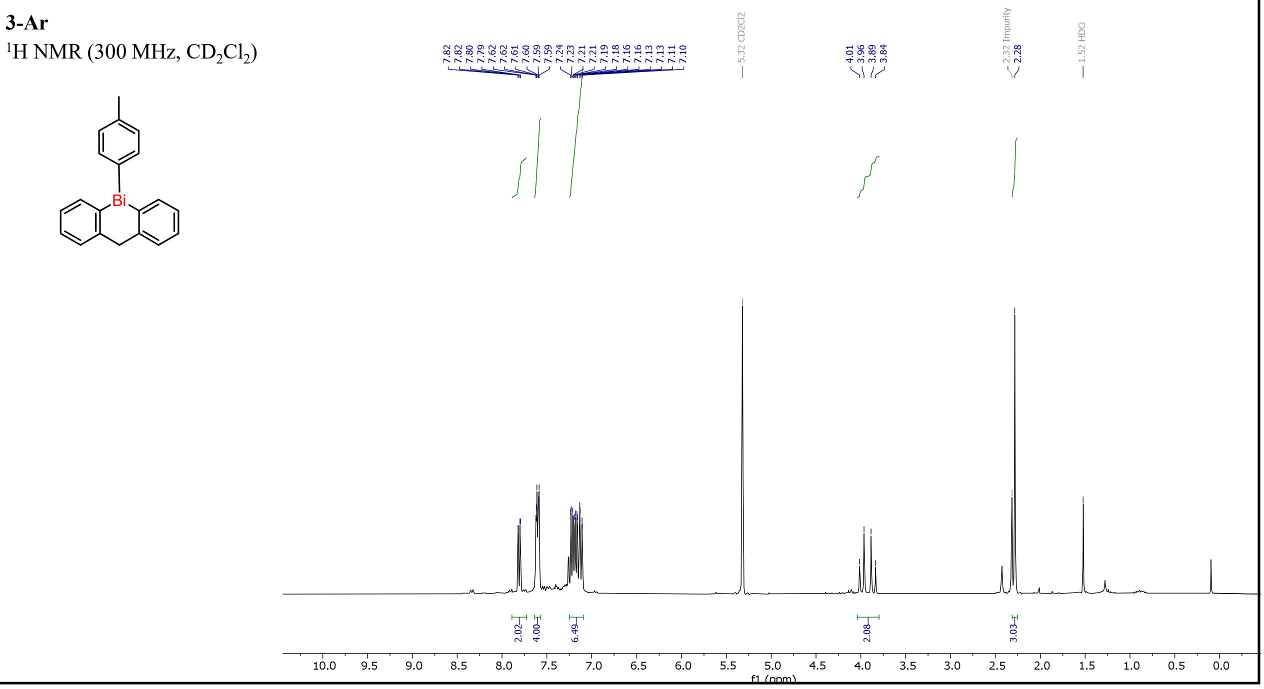

3-Ar

^{13}C NMR (75 MHz, CD_2Cl_2)

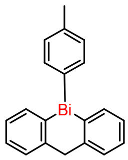

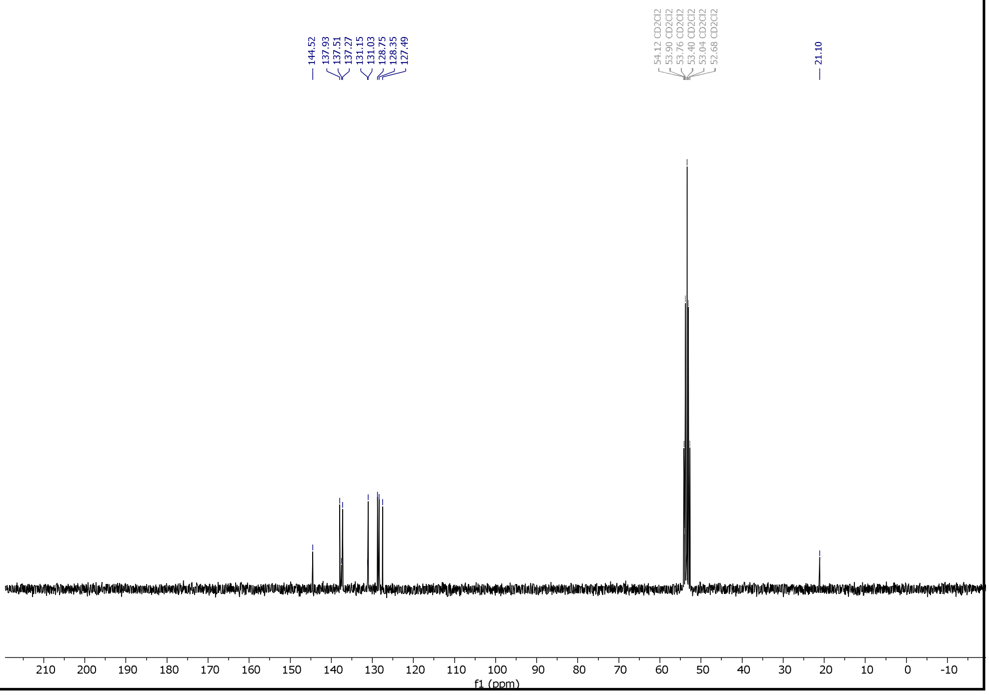

3-I

^{1}H NMR (300 MHz, DMSO)

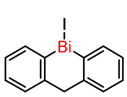

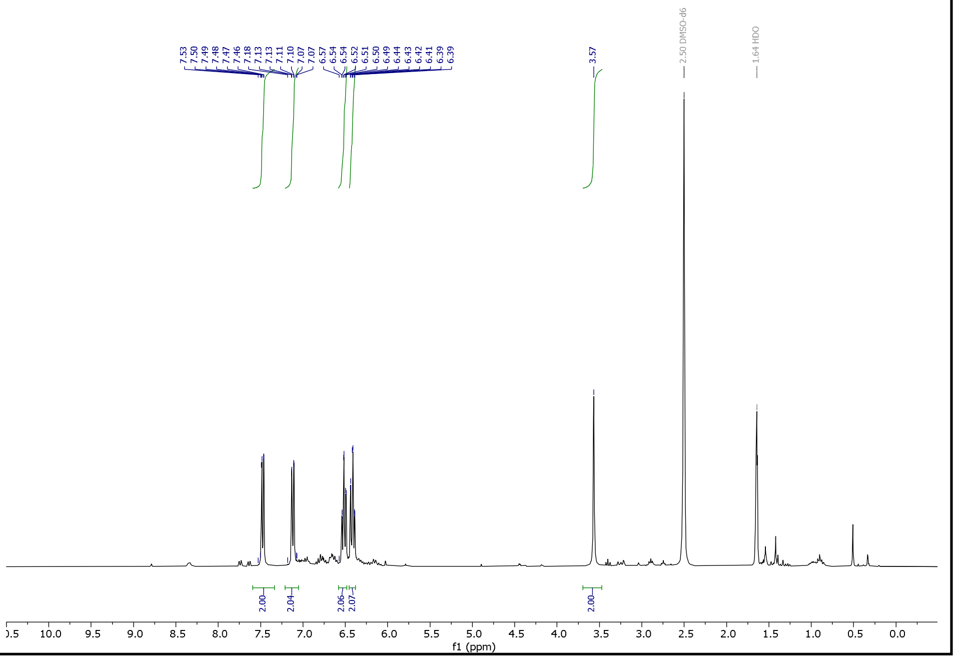

3-I

^{13}C NMR (75 MHz, DMSO)

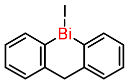

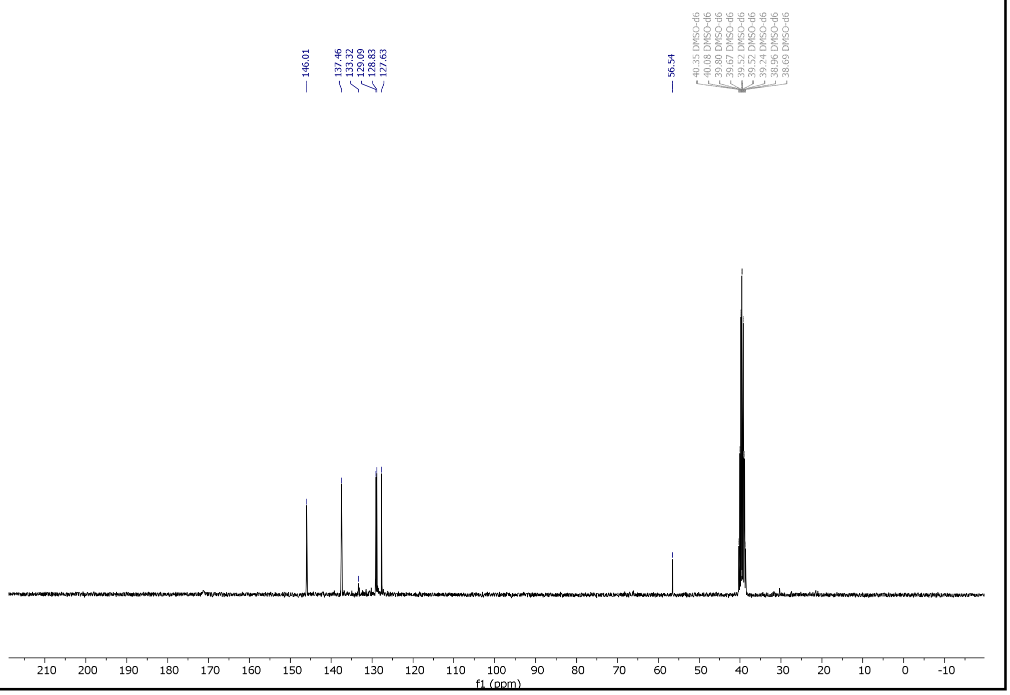

3

^{1}H NMR (300 MHz, CD_3CN)

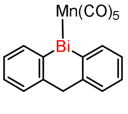

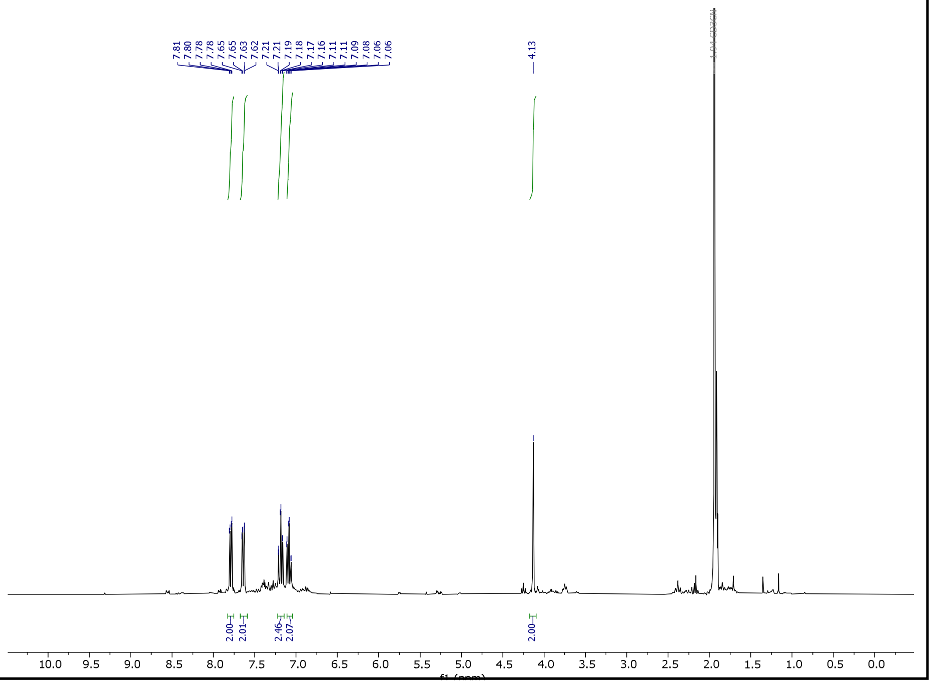

3

^{13}C NMR (75 MHz, CD_3CN)

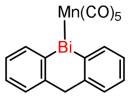

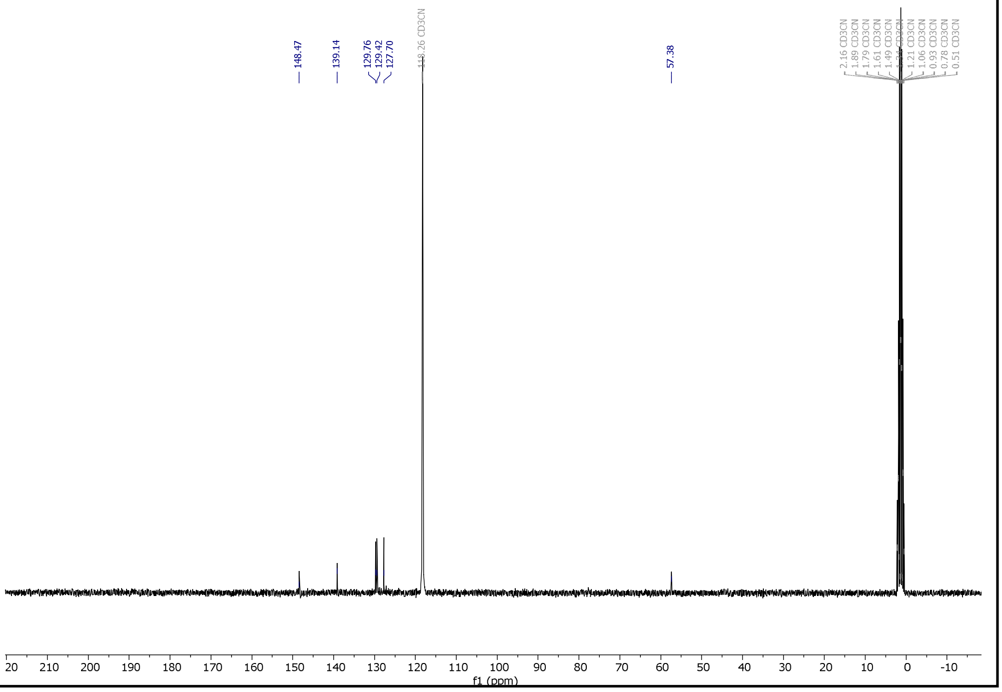

4-Ar

^{1}H NMR (300 MHz, CD_2Cl_2)

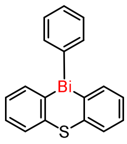

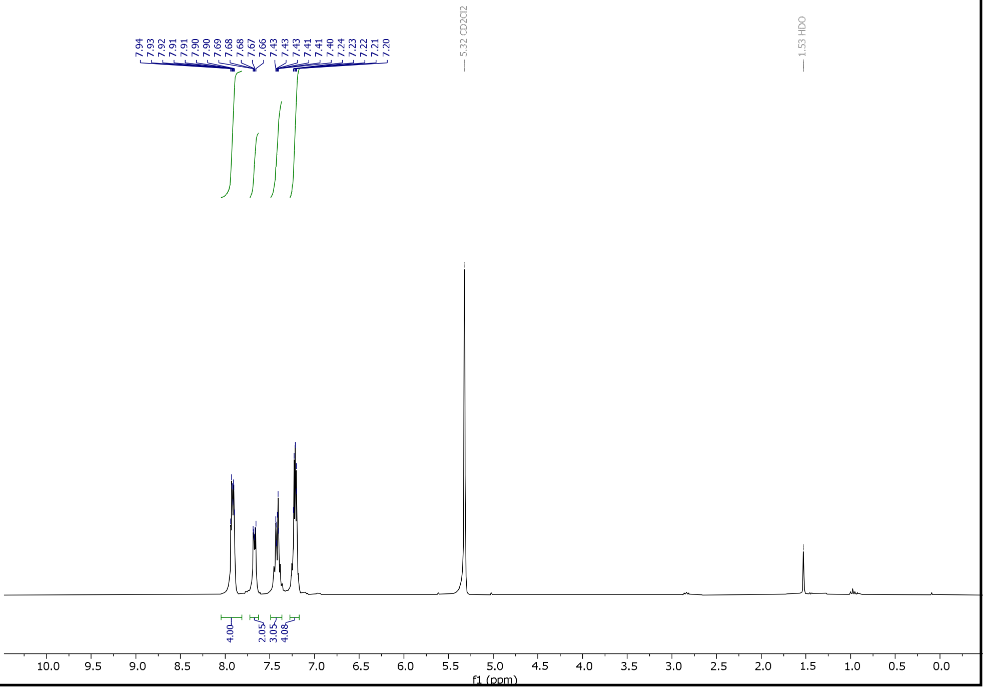

4-Ar

^{13}C NMR (75 MHz, CD_2Cl_2)

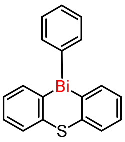

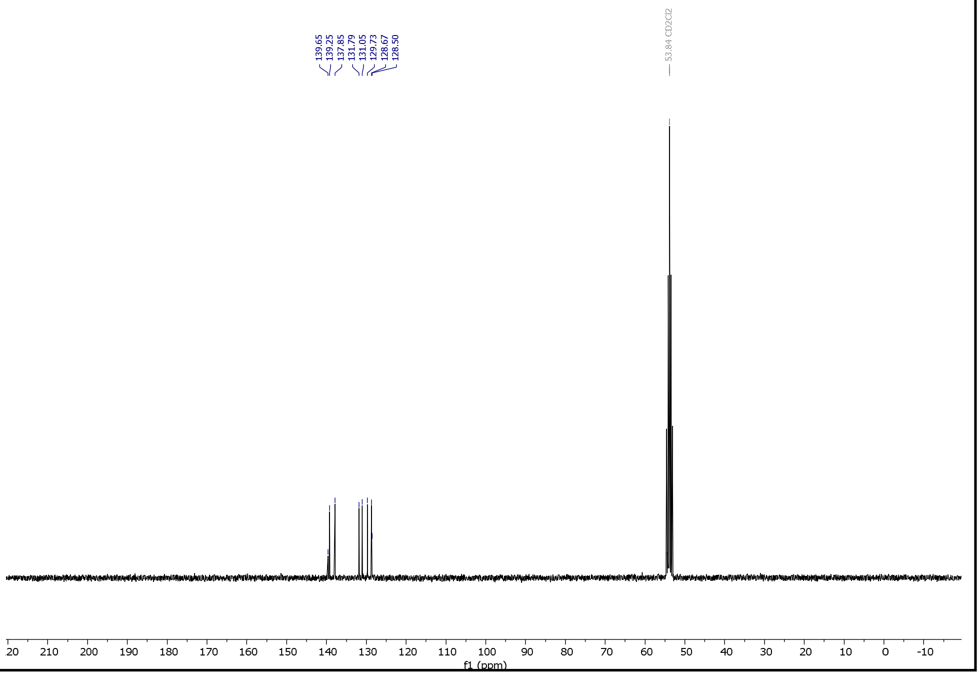

4-I

^{1}H NMR (300 MHz, CD_2Cl_2)

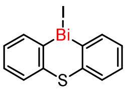

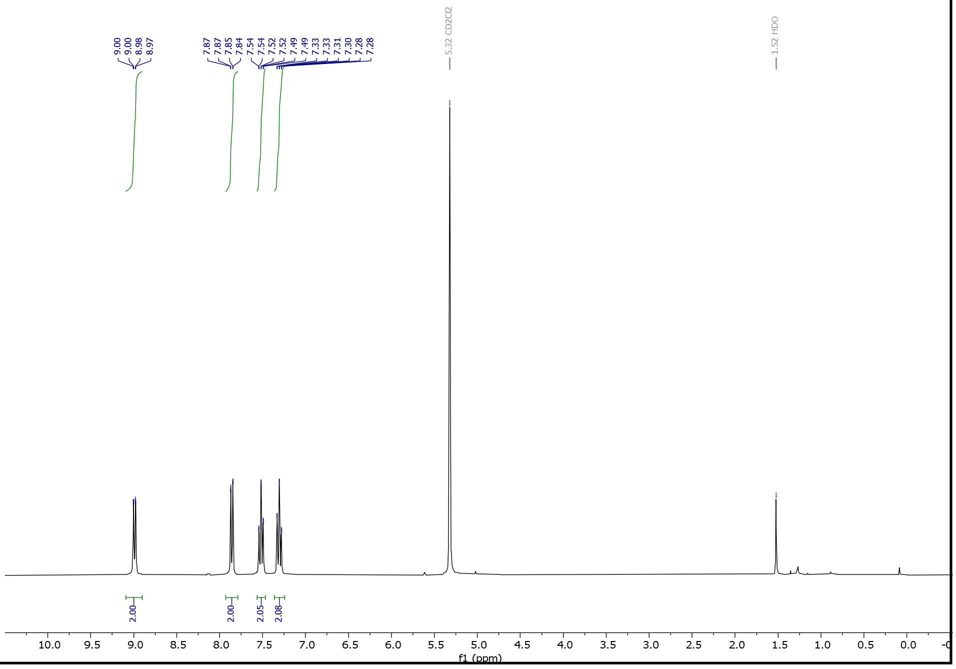

4-I

^{13}C NMR (75 MHz, CD_2Cl_2)

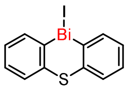

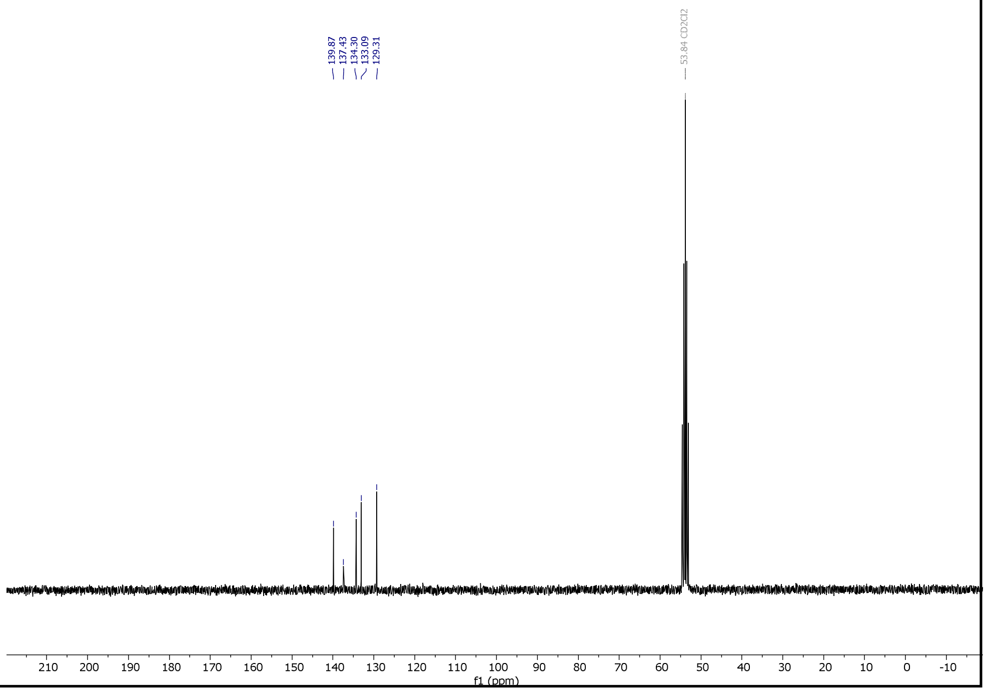

4

^{1}H NMR (300 MHz, CD_3CN)

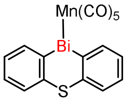

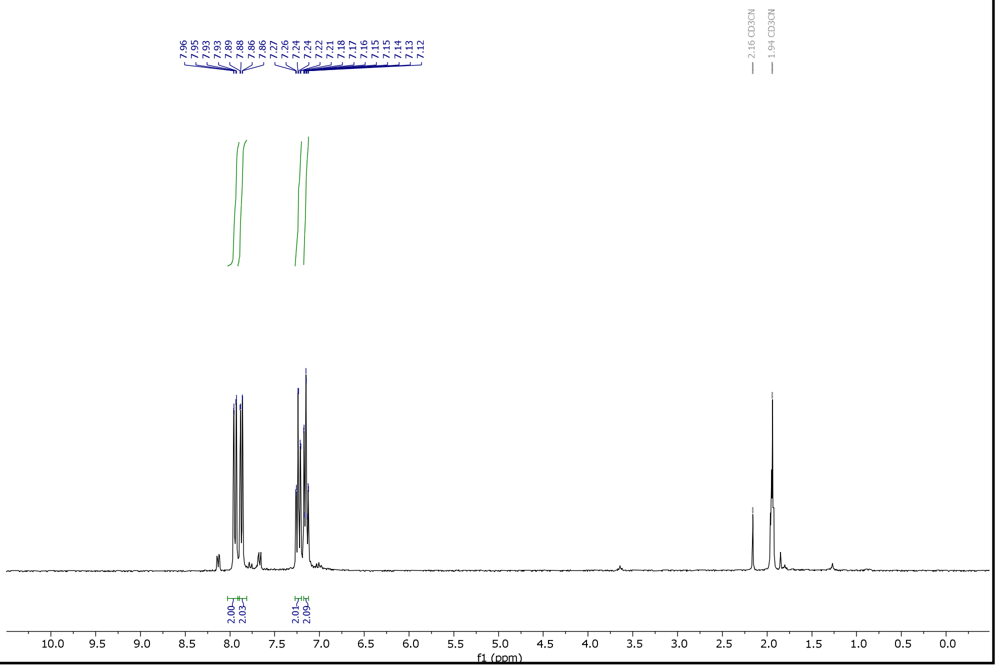

4

^{13}C NMR (75 MHz, CD_3CN)

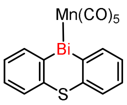

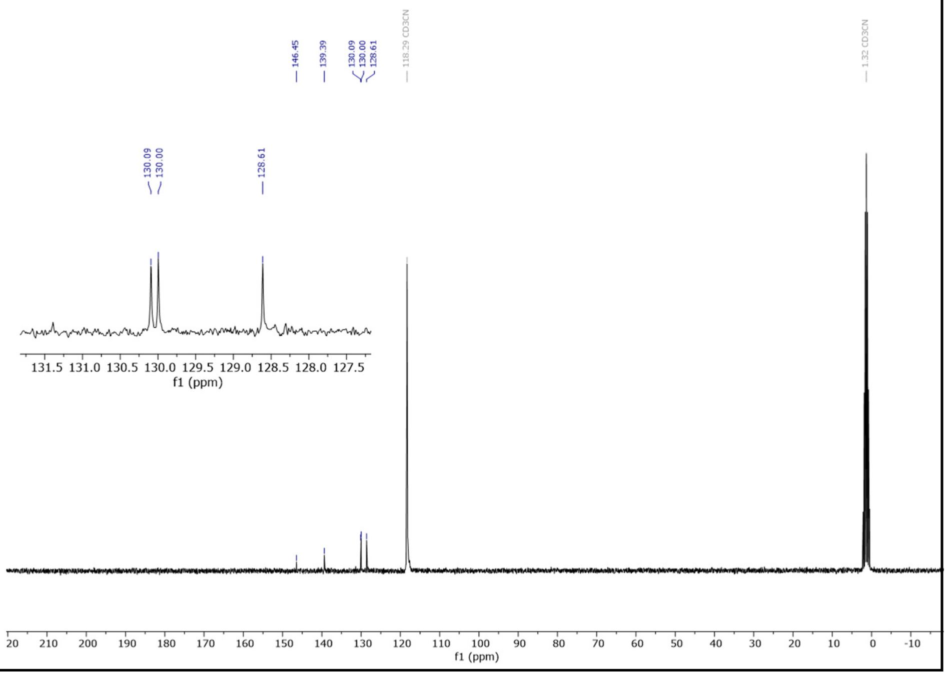

S3a

^{1}H NMR (300 MHz, $CDCl_3$)

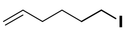

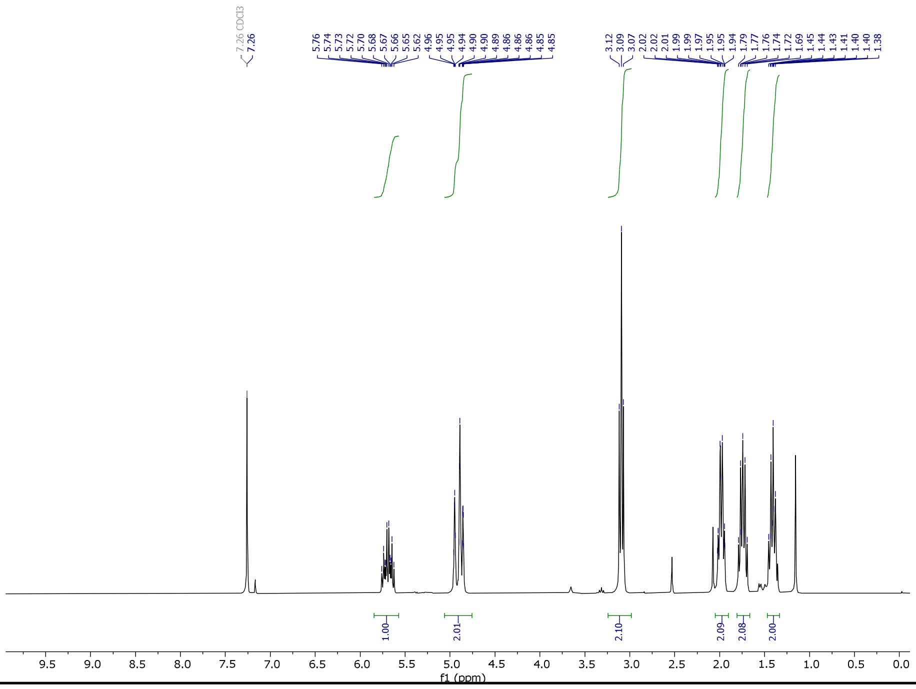

16a

^{1}H NMR (300 MHz, $CDCl_3$)

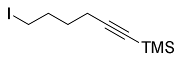

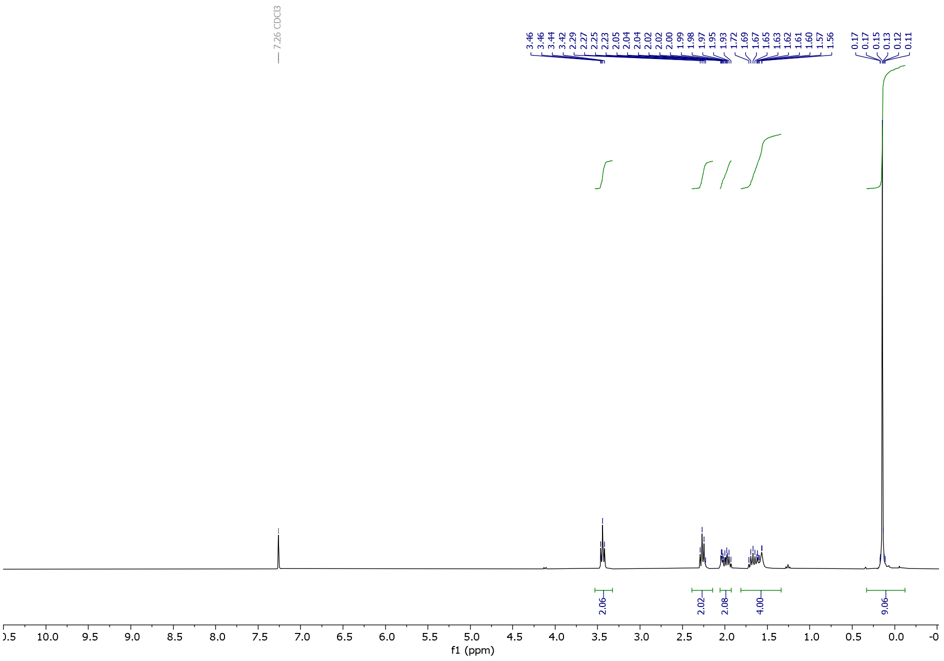

19a

^{1}H NMR (300 MHz, CD_2Cl_2)

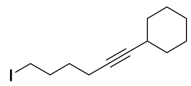

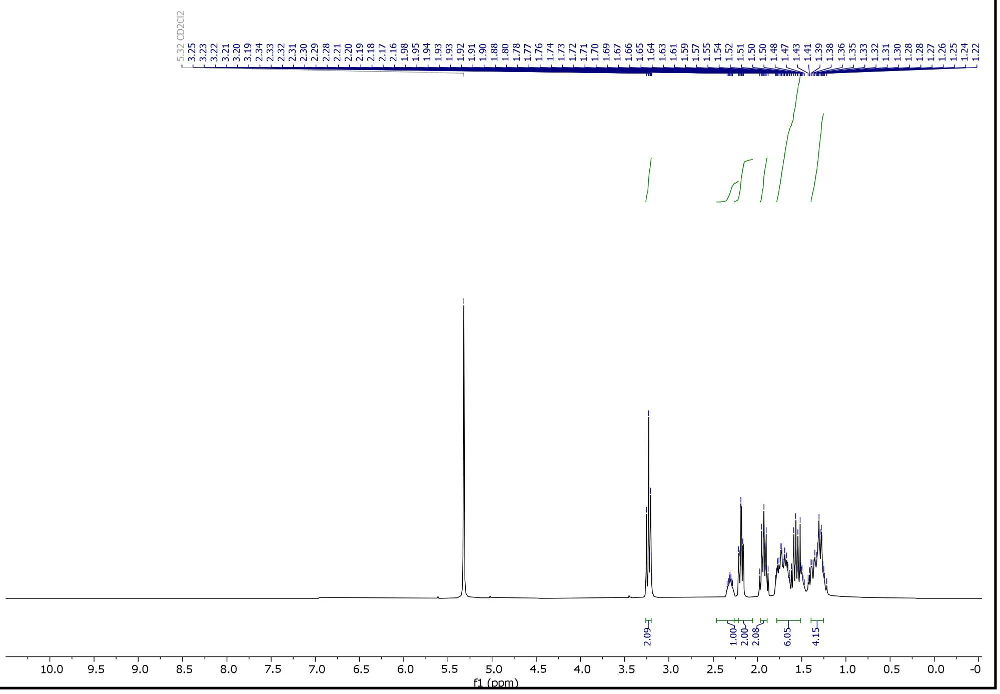

20a

^{1}H NMR (300 MHz, CD_2Cl_2)

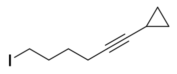

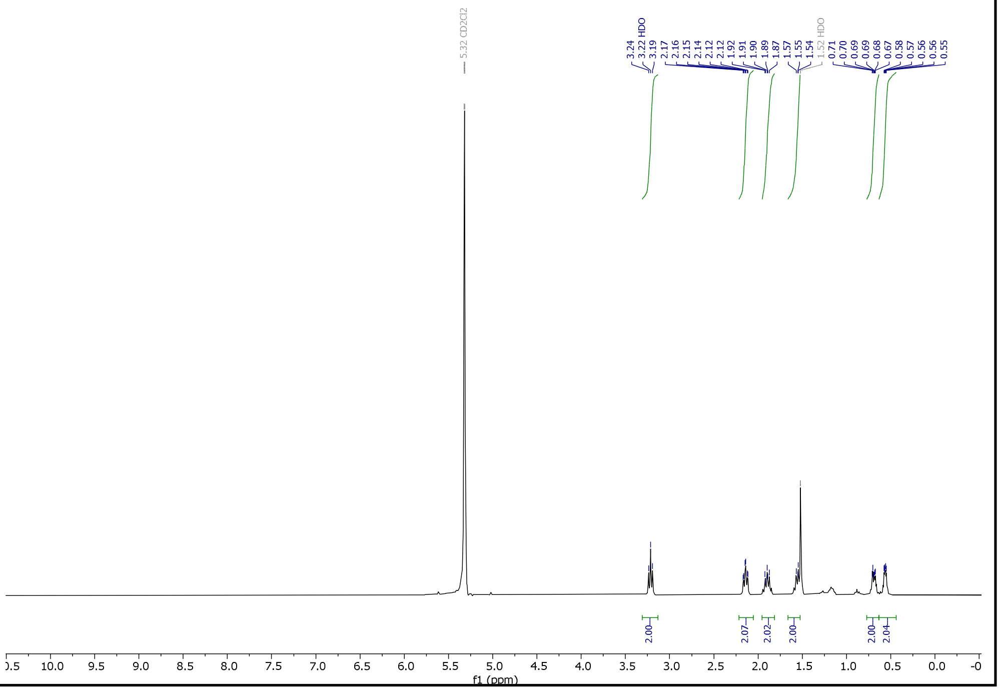

21a

^{1}H NMR (300 MHz, $CDCl_3$

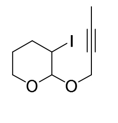

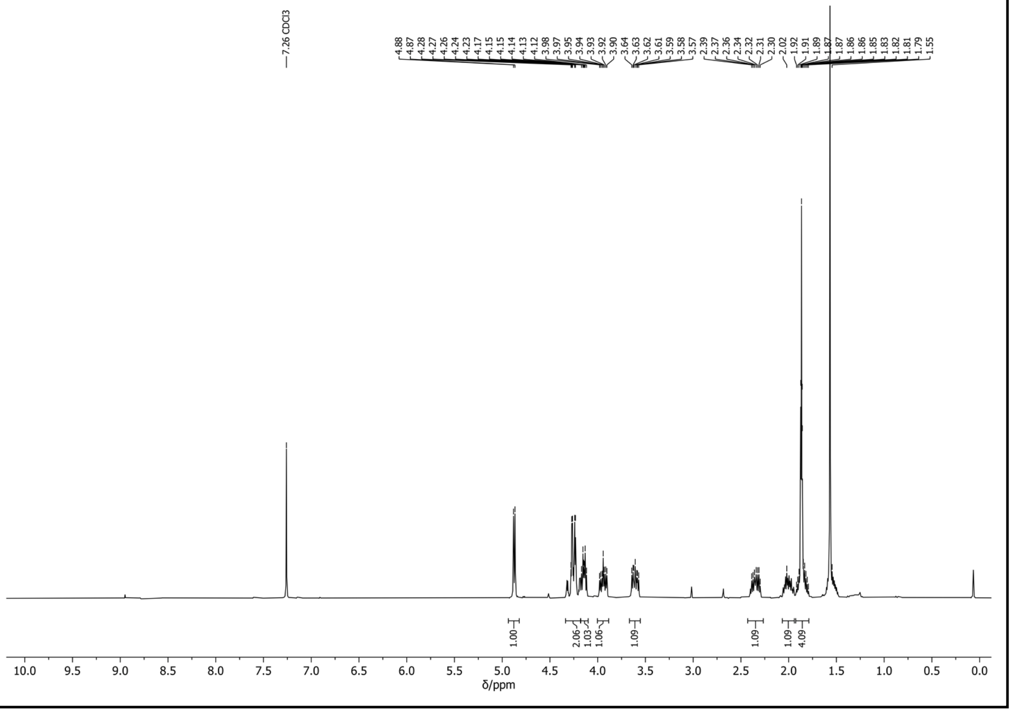

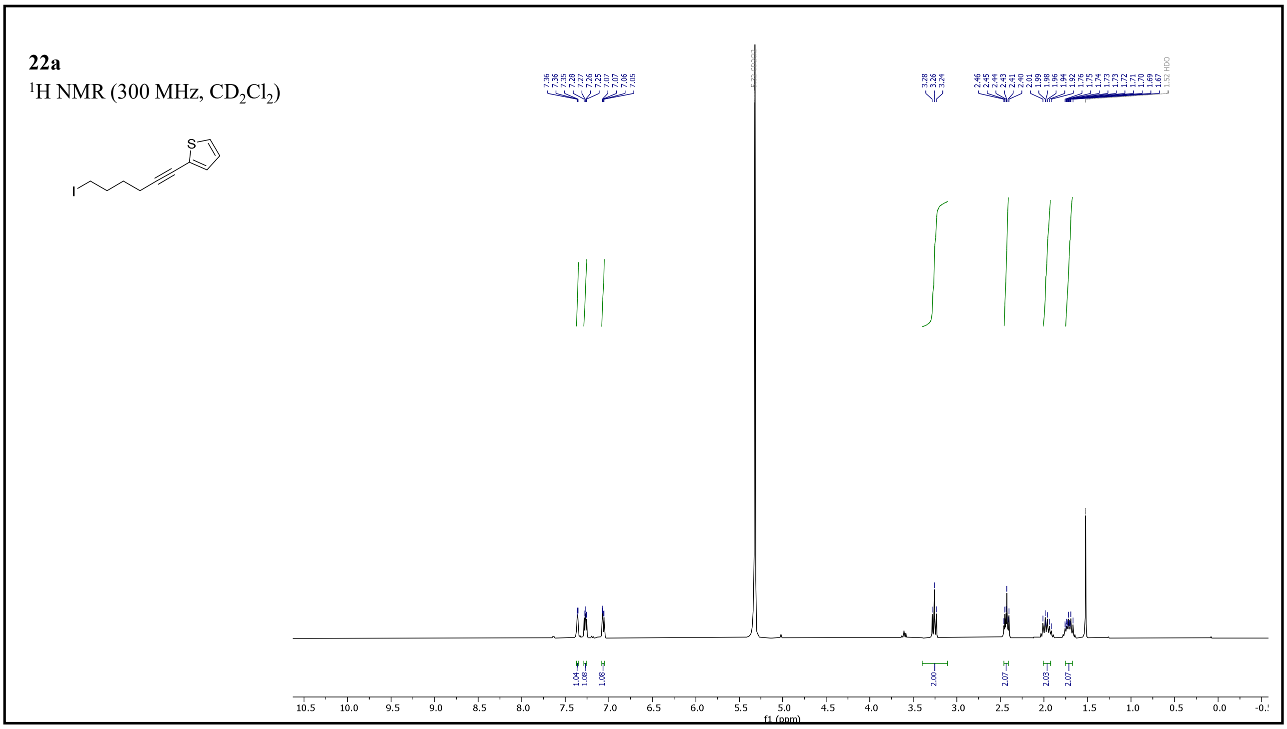
22a
^{1}H NMR (300 MHz, CD_2Cl_2)
f1 (ppm)

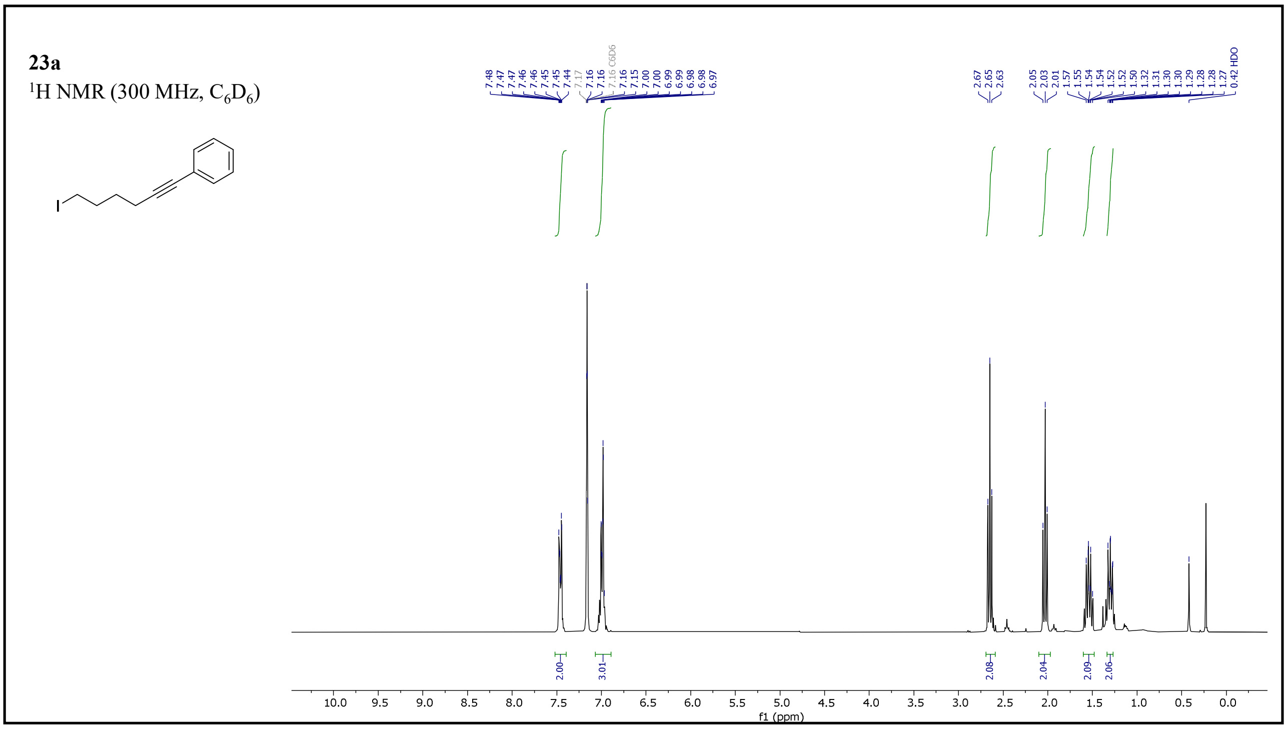
23a
^{1}H NMR (300 MHz, C_6D_6)
f1 (ppm)

24a

^{1}H NMR (300 MHz, CD_2Cl_2)

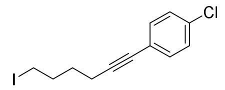

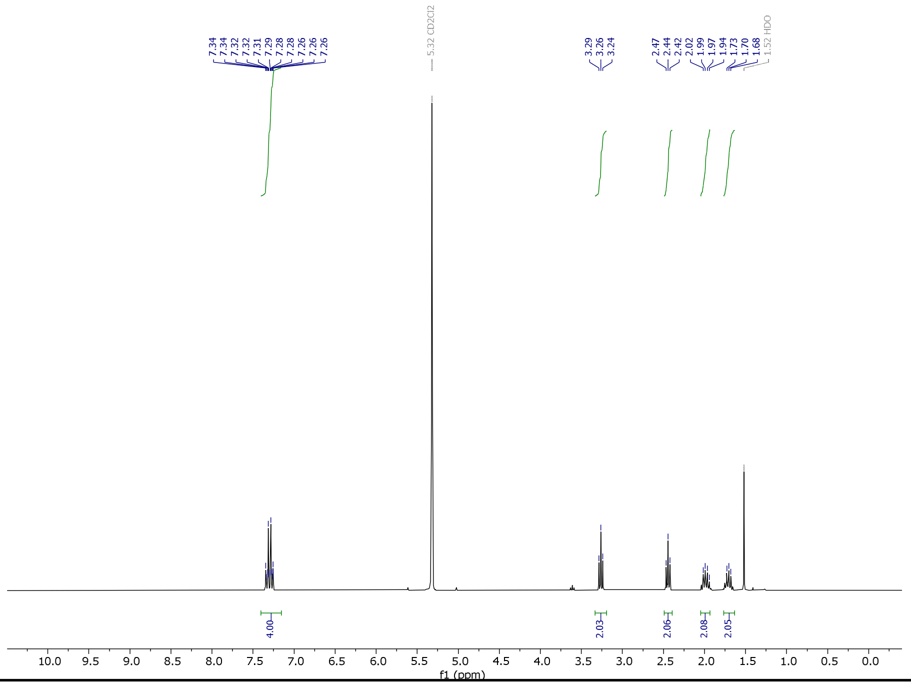

25a

^{1}H NMR (300 MHz, CD_2Cl_2)

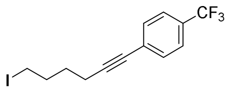

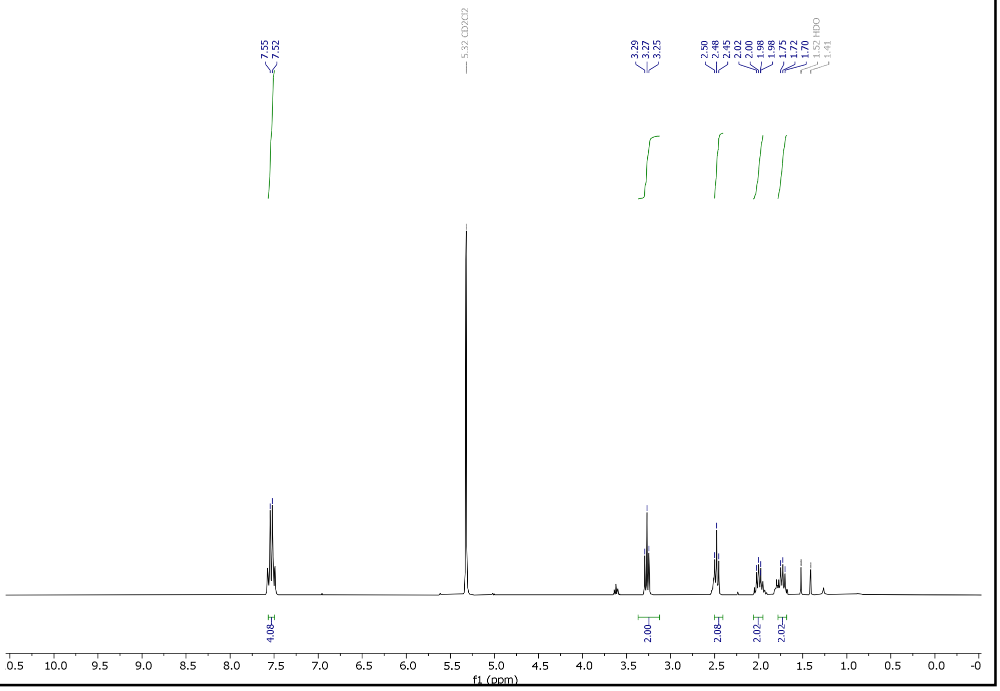

26a

^{1}H NMR (300 MHz, CD_2Cl_2)

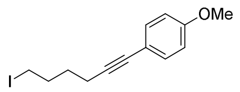

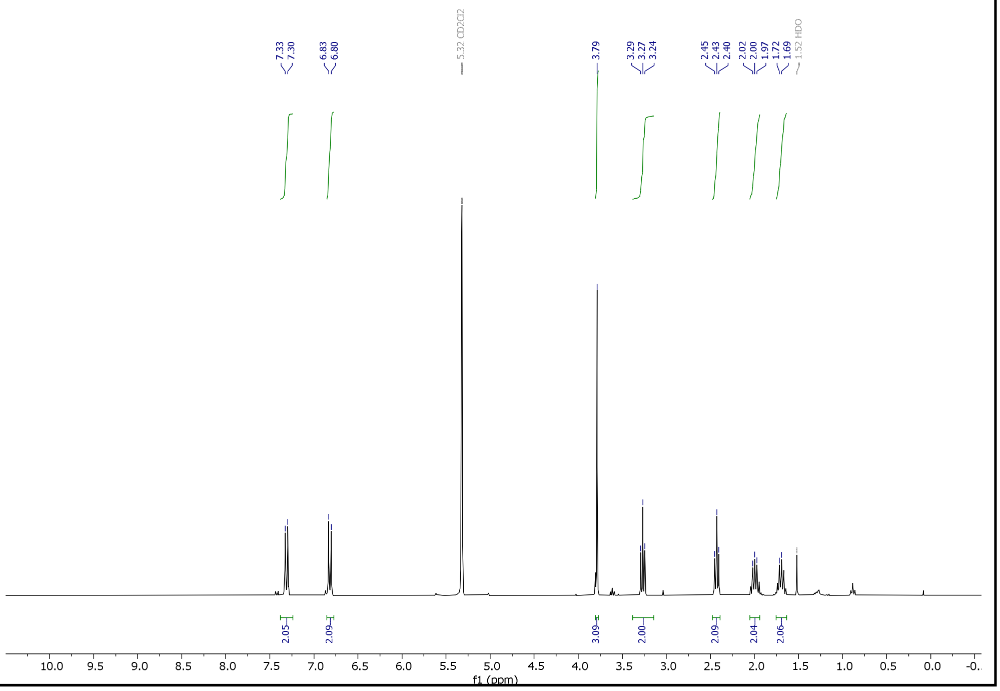

27a

^{1}H NMR (300 MHz, CD_2Cl_2)

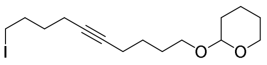

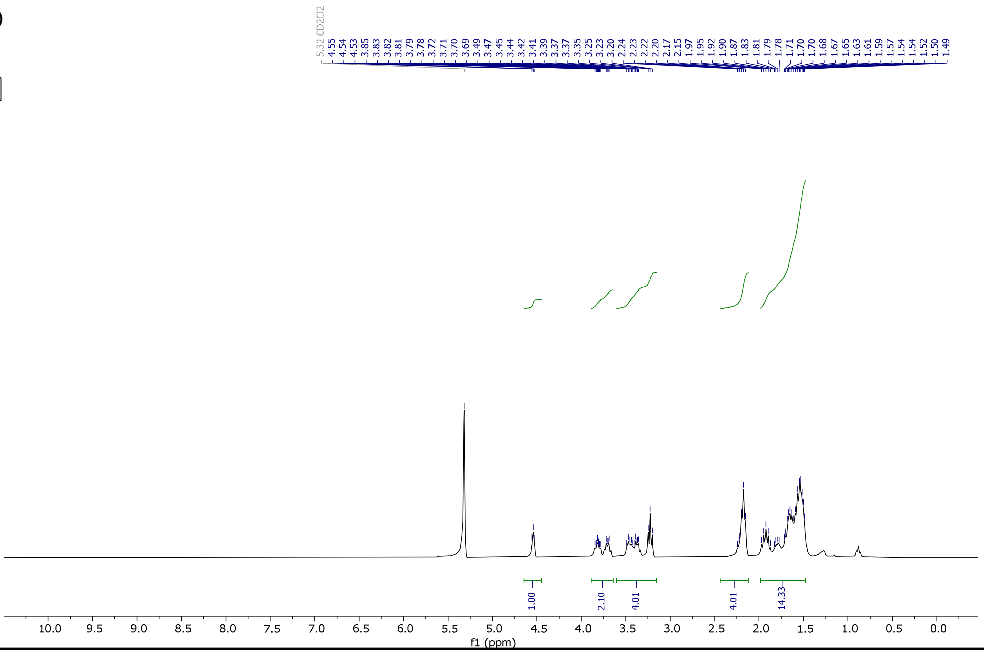

28a

^{1}H NMR (300 MHz, CD_2Cl_2)

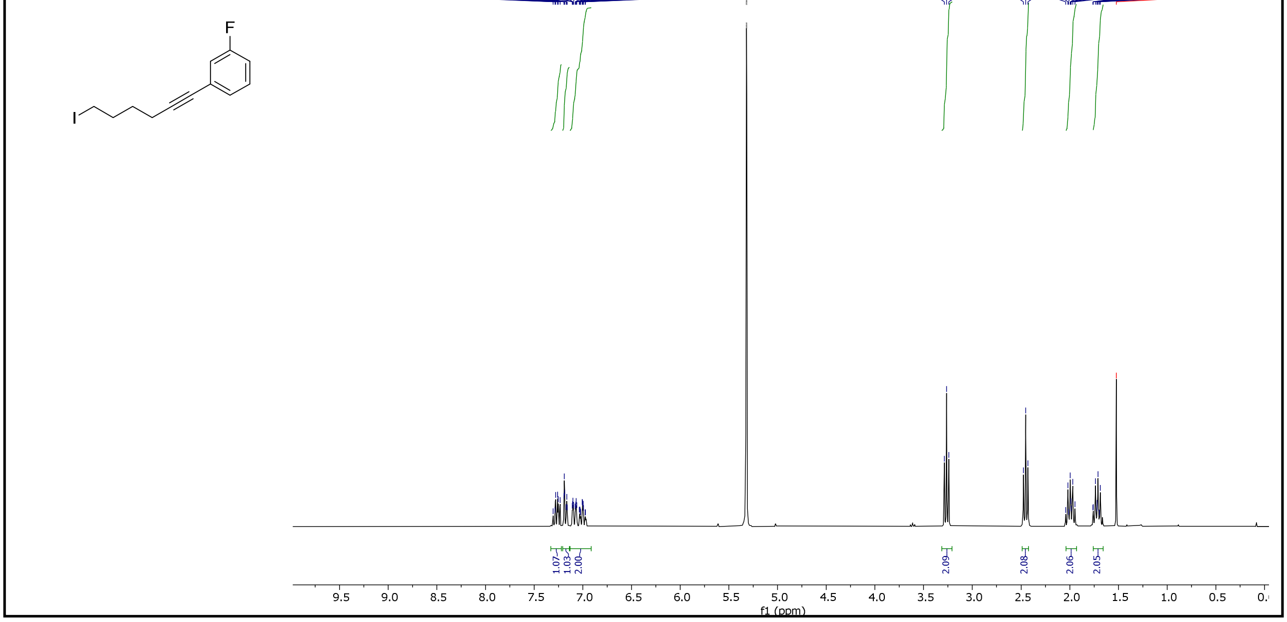

29a

^{1}H NMR (300 MHz, C_6D_6)

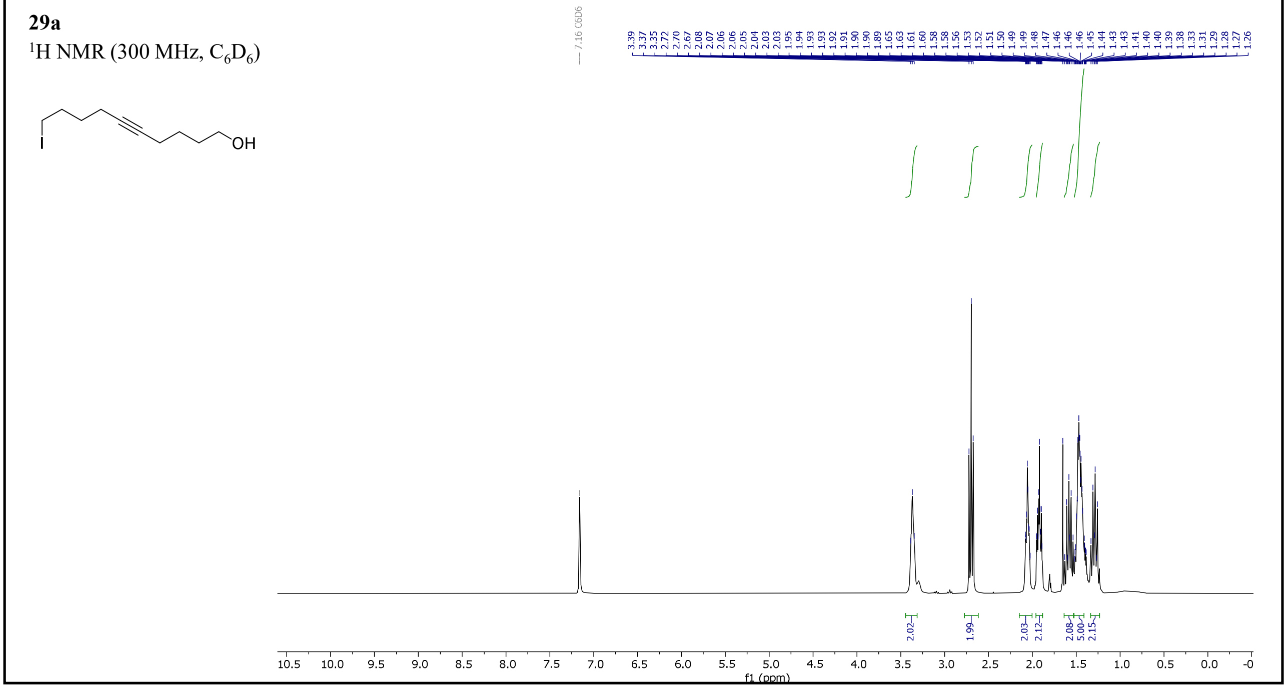

30a

^{1}H NMR (300 MHz, $CDCl_3$)

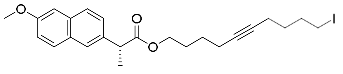

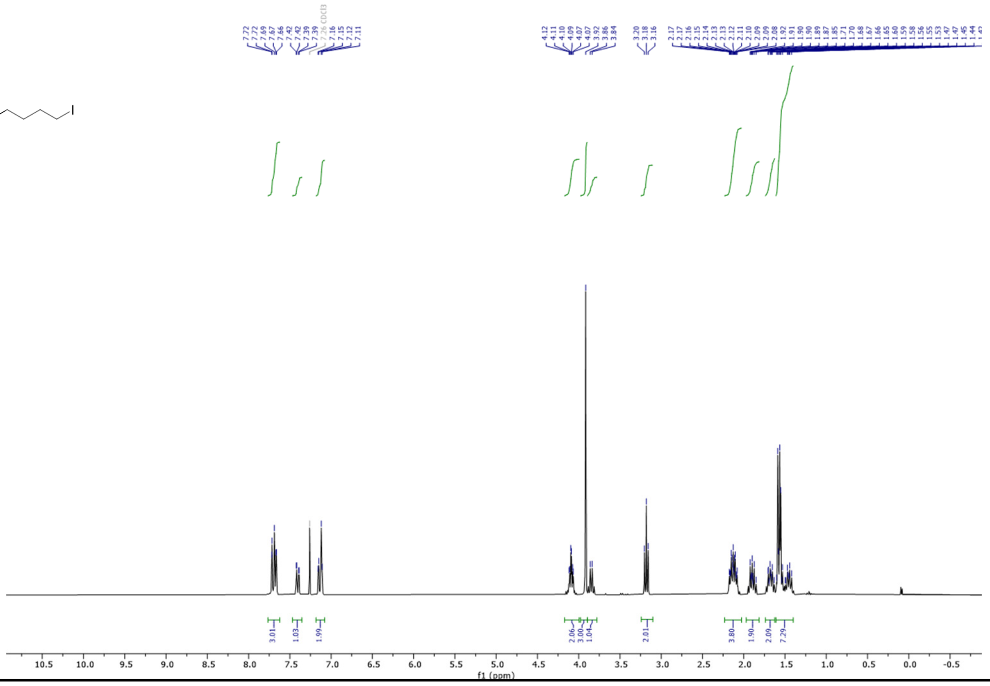

30a

^{13}C NMR (75 MHz, $CDCl_3$)

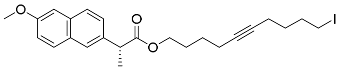

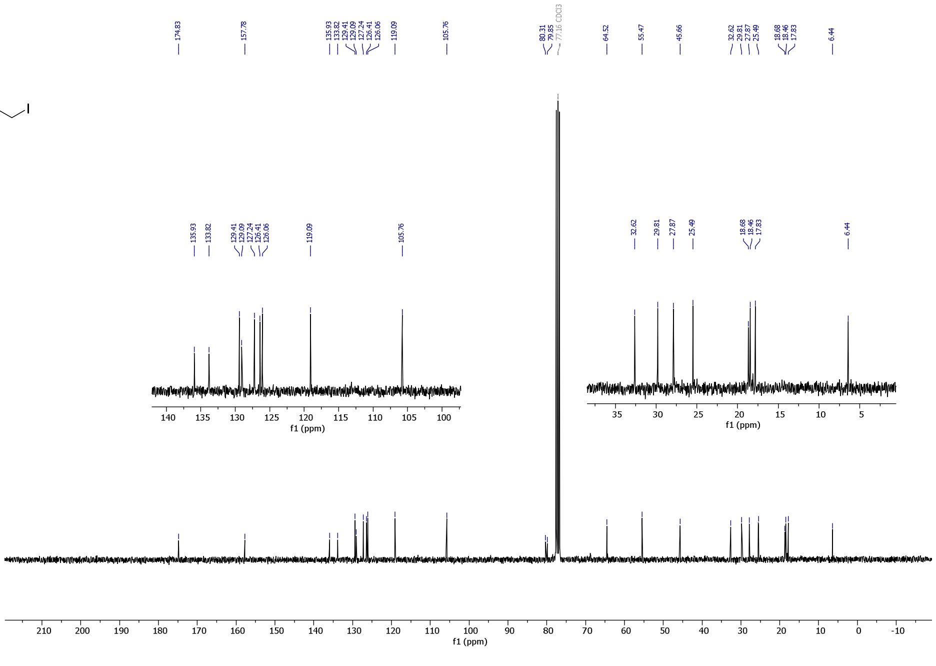

31a

^{1}H NMR (300 MHz, $CDCl_3$)

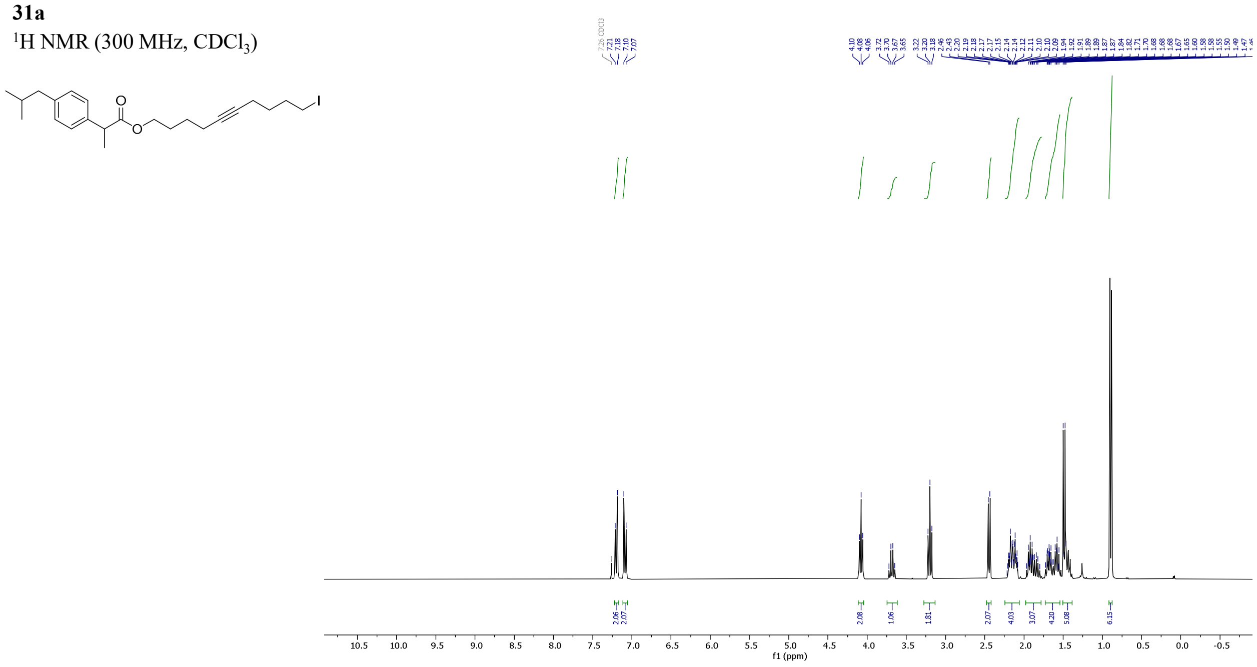

31a

^{13}C NMR (75 MHz, $CDCl_3$)

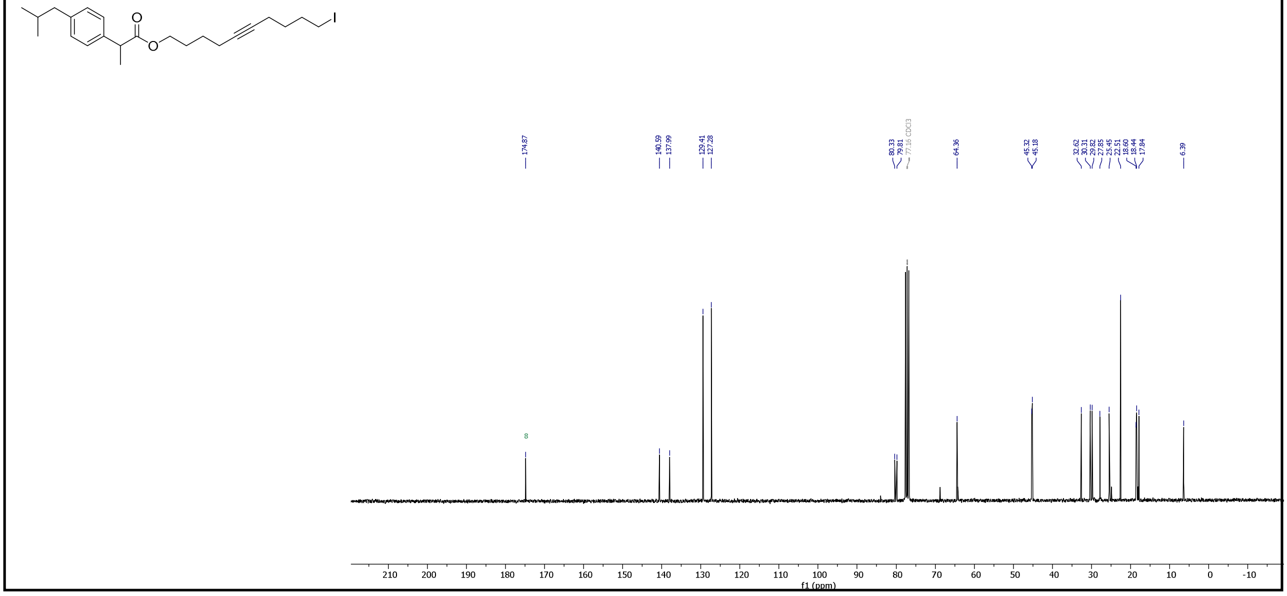

32a

^{1}H NMR (300 MHz, $CDCl_3$)

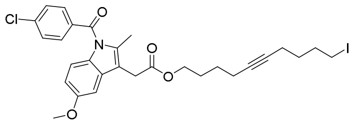

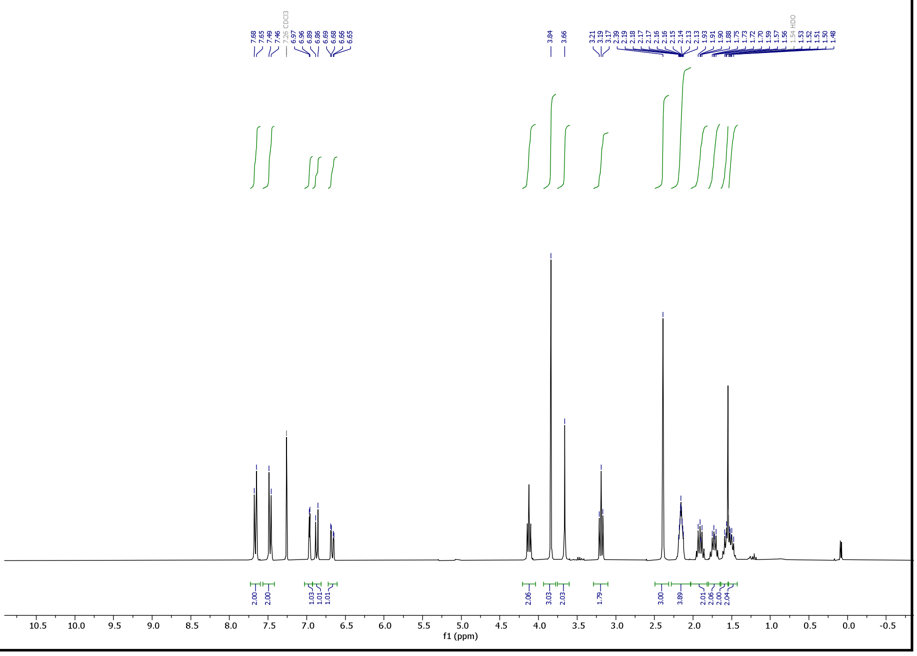

32a

^{13}C NMR (75 MHz, $CDCl_3$)

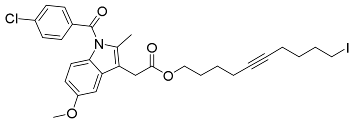

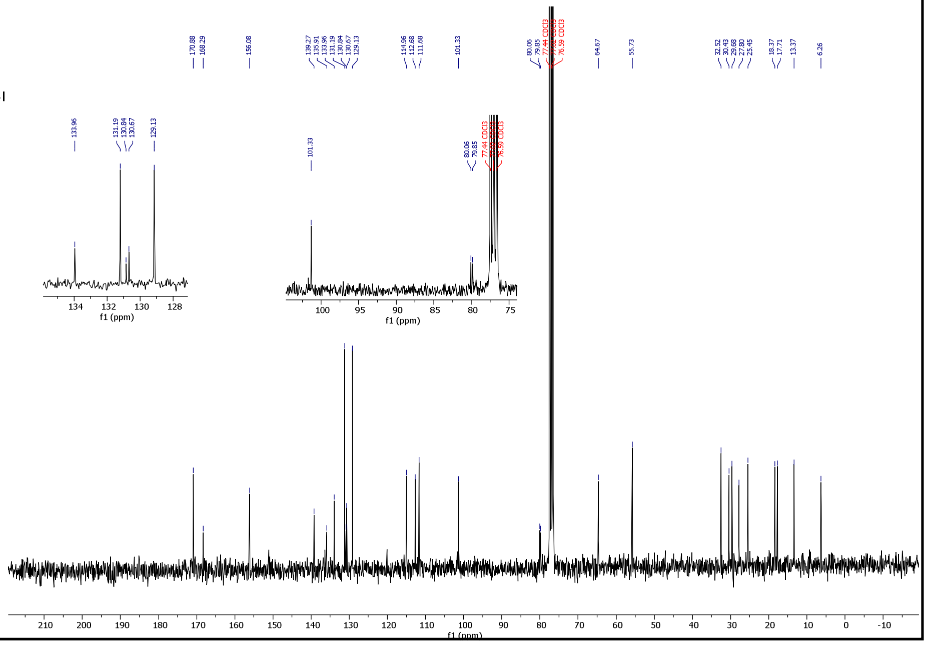

S3b

^{1}H NMR (250 MHz, C_6D_6)

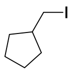

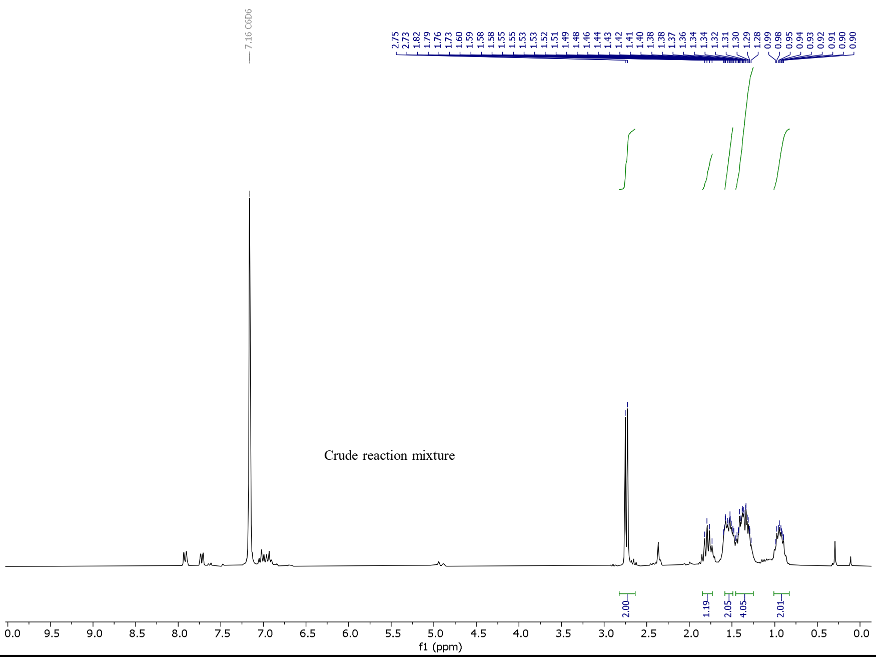

16b

^{1}H NMR (250 MHz, C_6D_6)

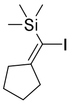

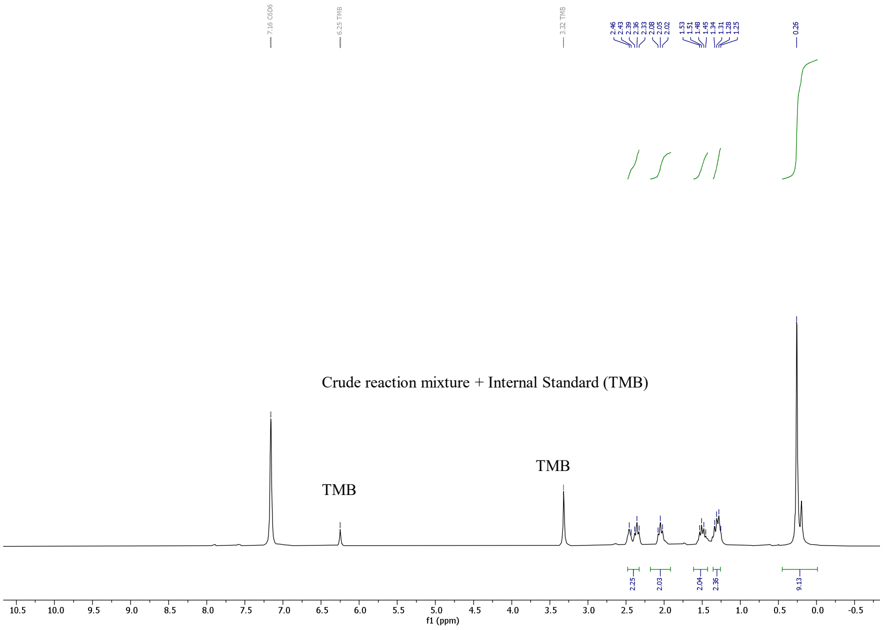

19b

^{1}H NMR (300 MHz, $CDCl_3$)

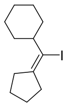

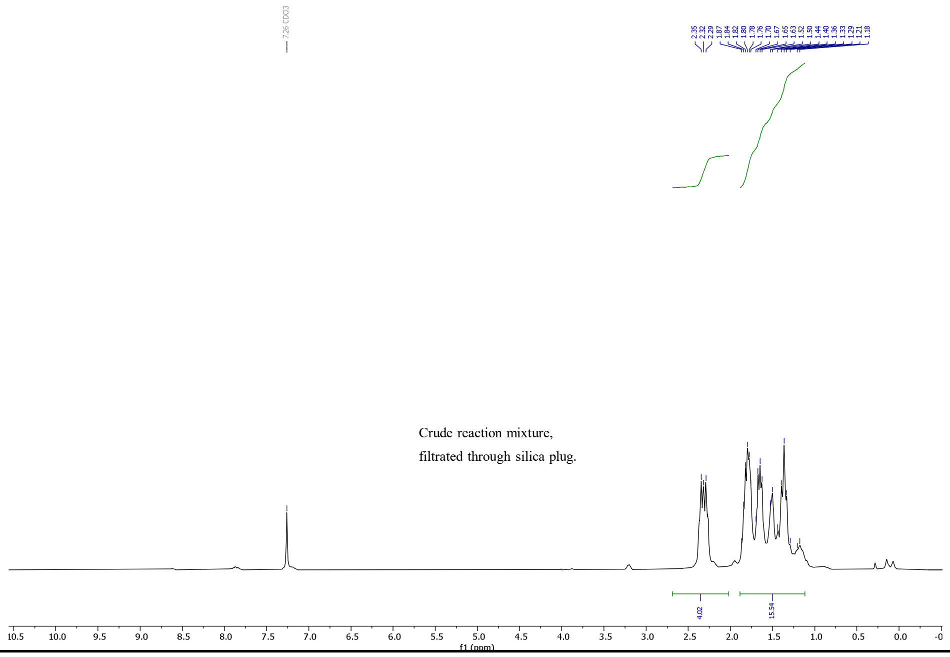

20b

^{1}H NMR (250 MHz, C_6D_6)

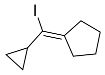

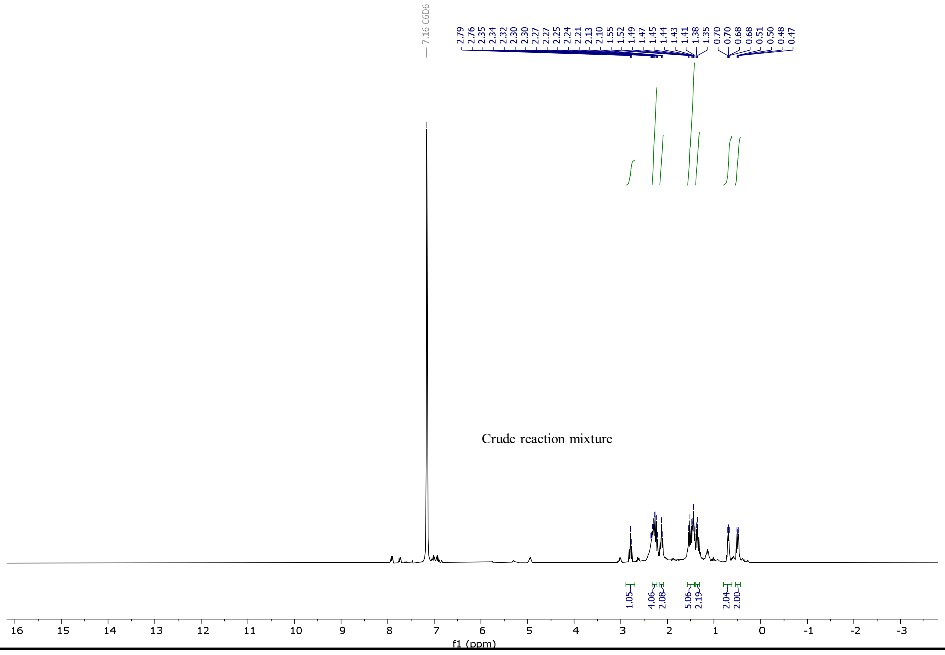

21b

^{1}H NMR (250 MHz, $CDCl_3$)

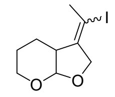

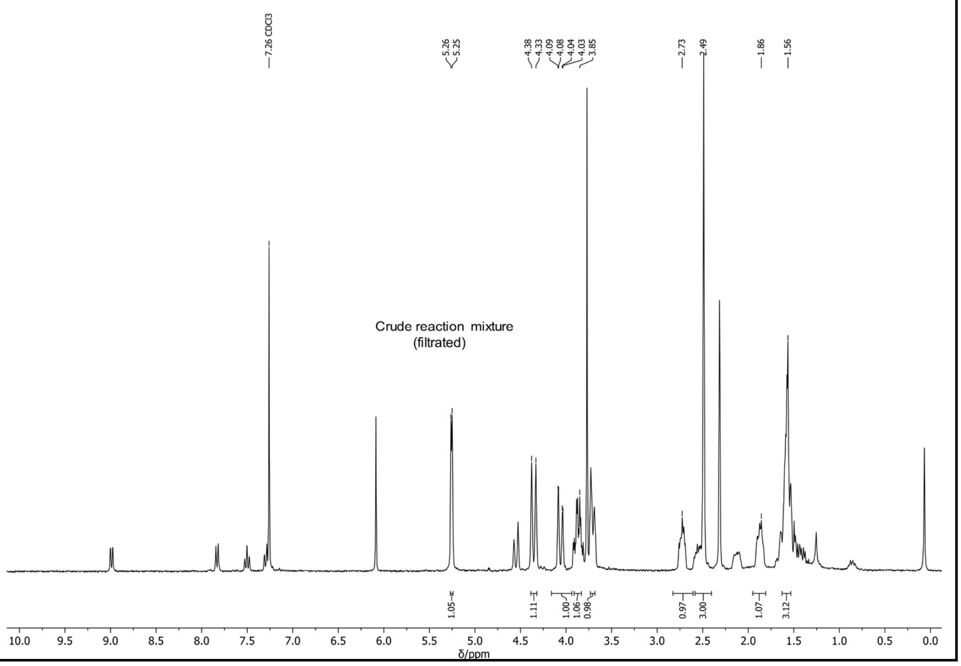

22b

^{1}H NMR (300 MHz, $CDCl_3$)

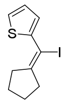

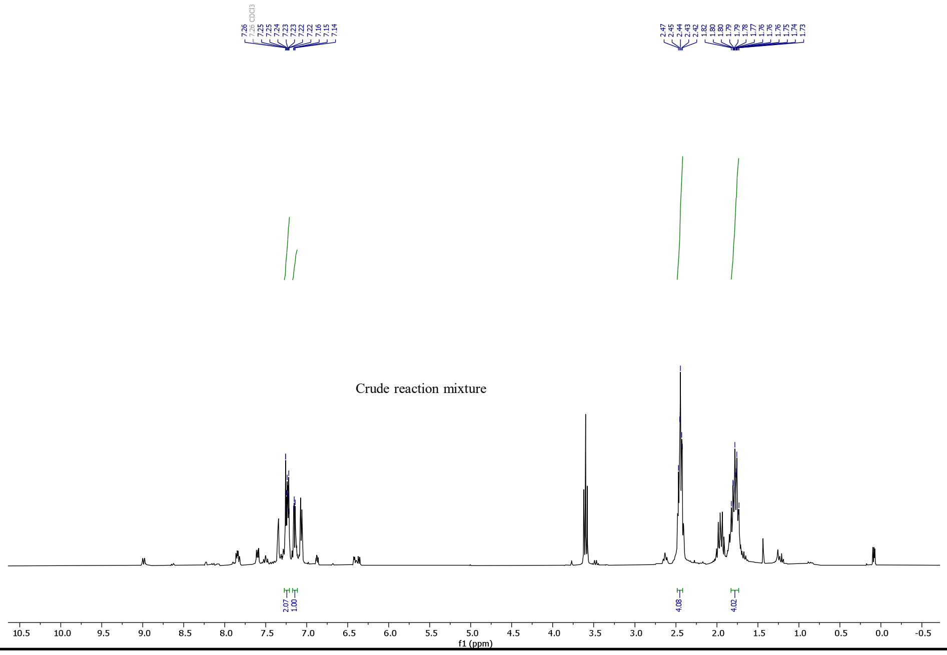

23b

^{1}H NMR (300 MHz, $CDCl_3$)

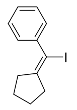

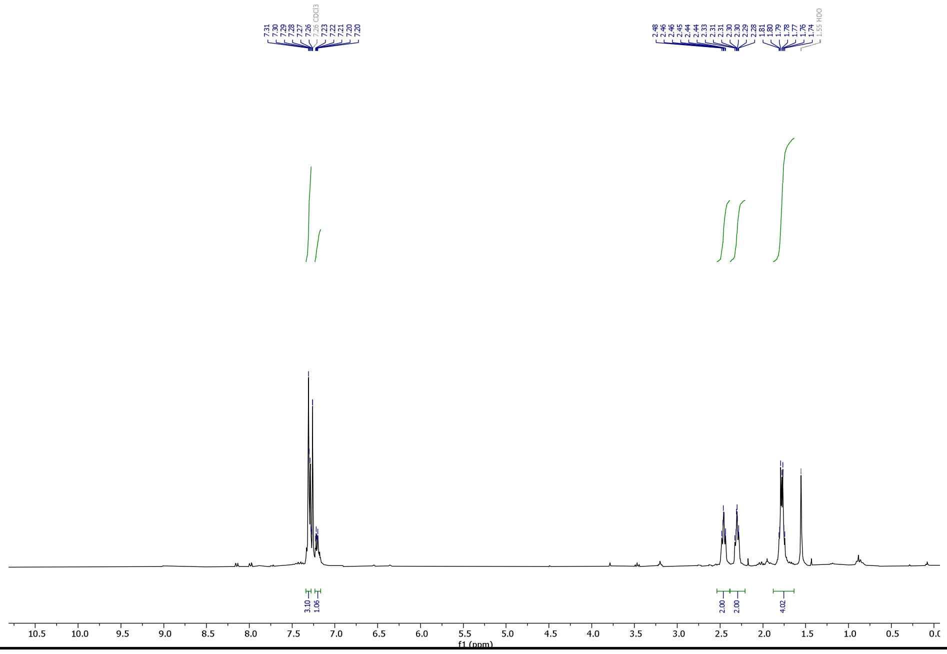

24b

^{1}H NMR (300 MHz, $CDCl_3$)

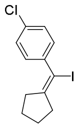

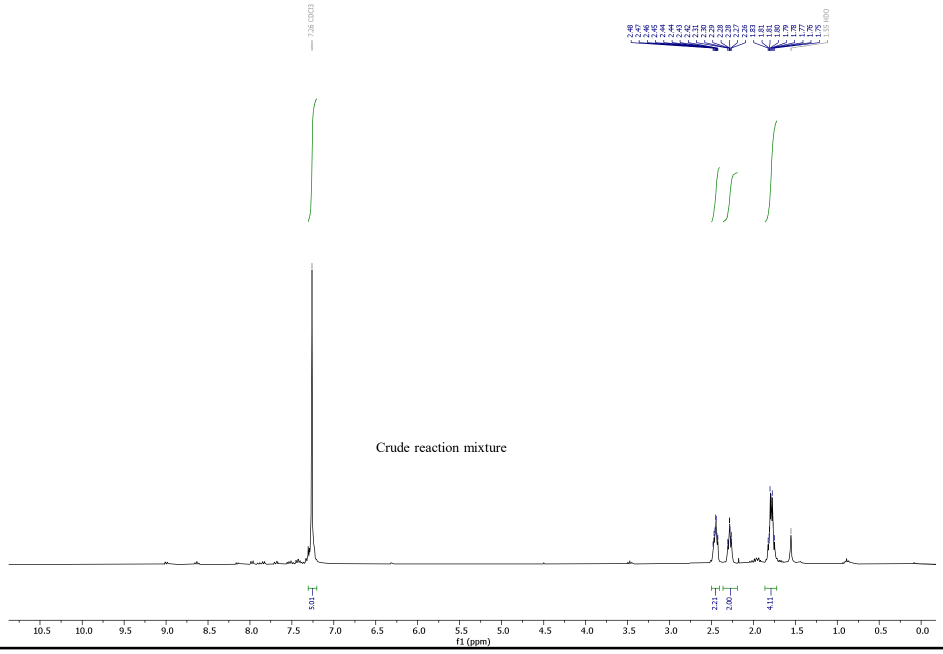

25b

^{1}H NMR (300 MHz, $CDCl_3$)

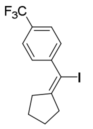

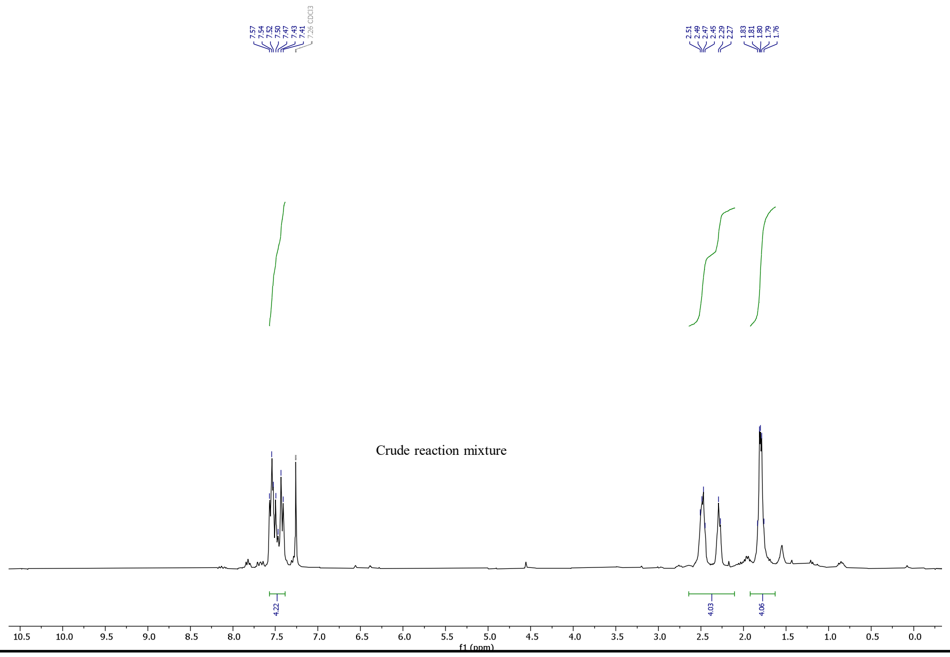

25b

^{19}F NMR (282 MHz, $CDCl_3$)

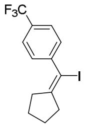

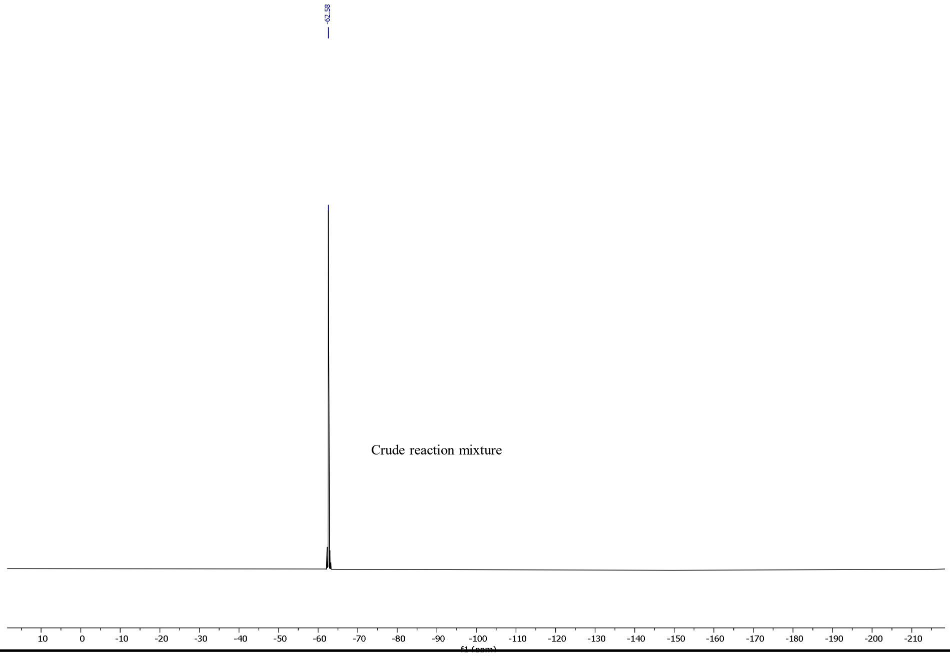

26b

^{1}H NMR (300 MHz, $CDCl_3$)

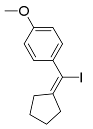

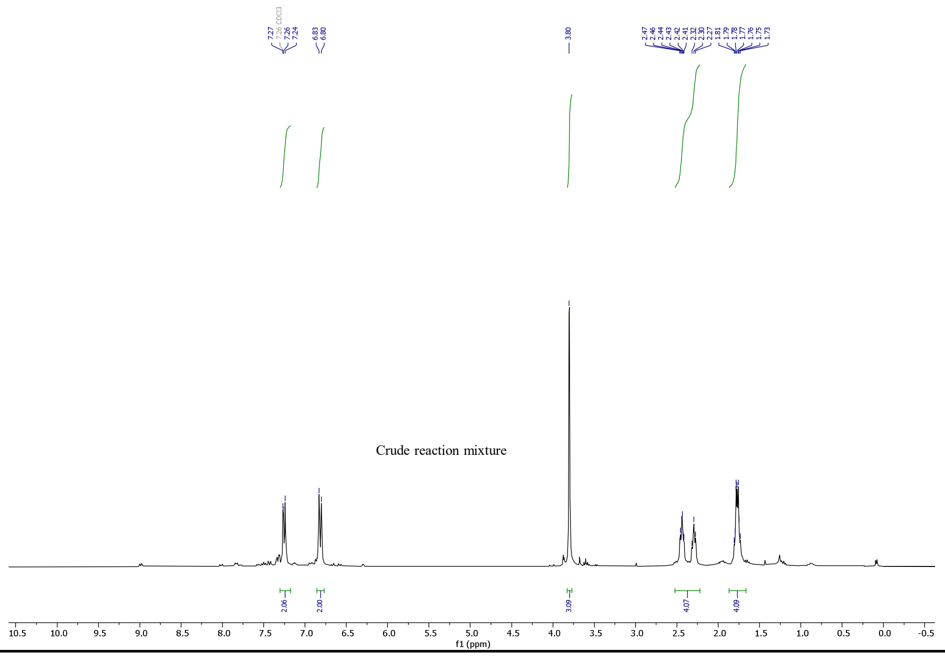

27b

^{1}H NMR (250 MHz, C_6D_6)

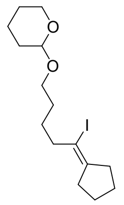

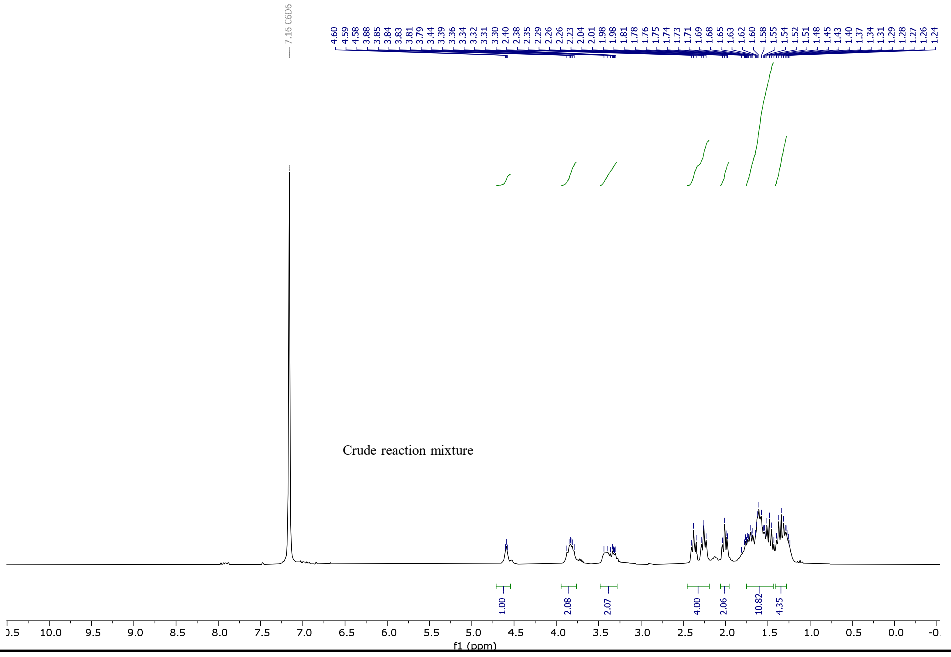

28b

^{1}H NMR (300 MHz, $CDCl_3$)

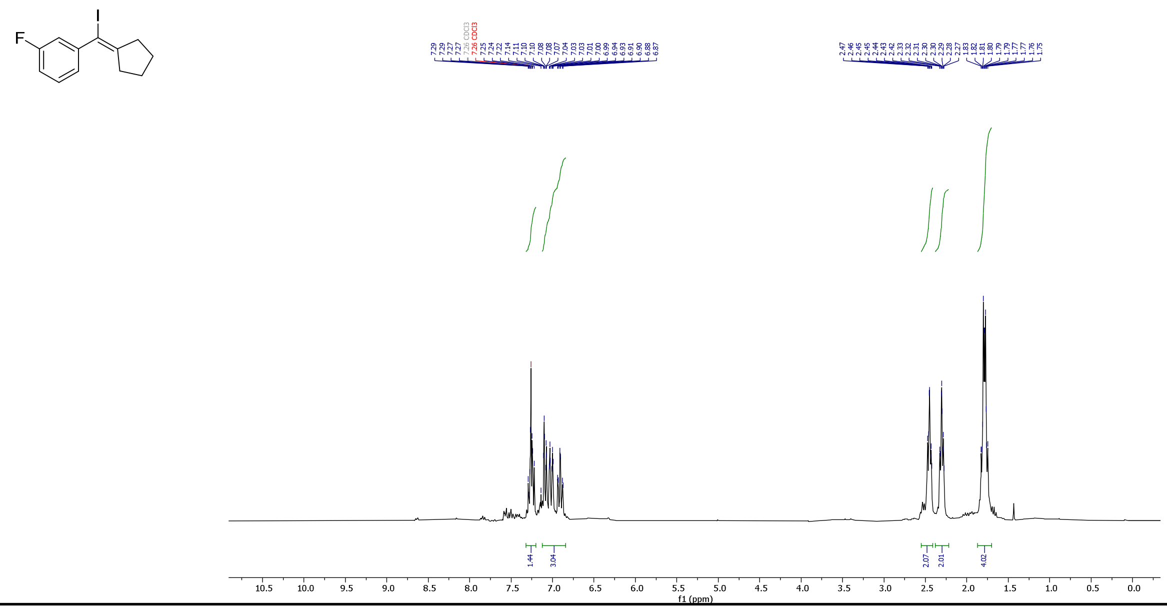

28b

^{13}C NMR (75 MHz, $CDCl_3$)

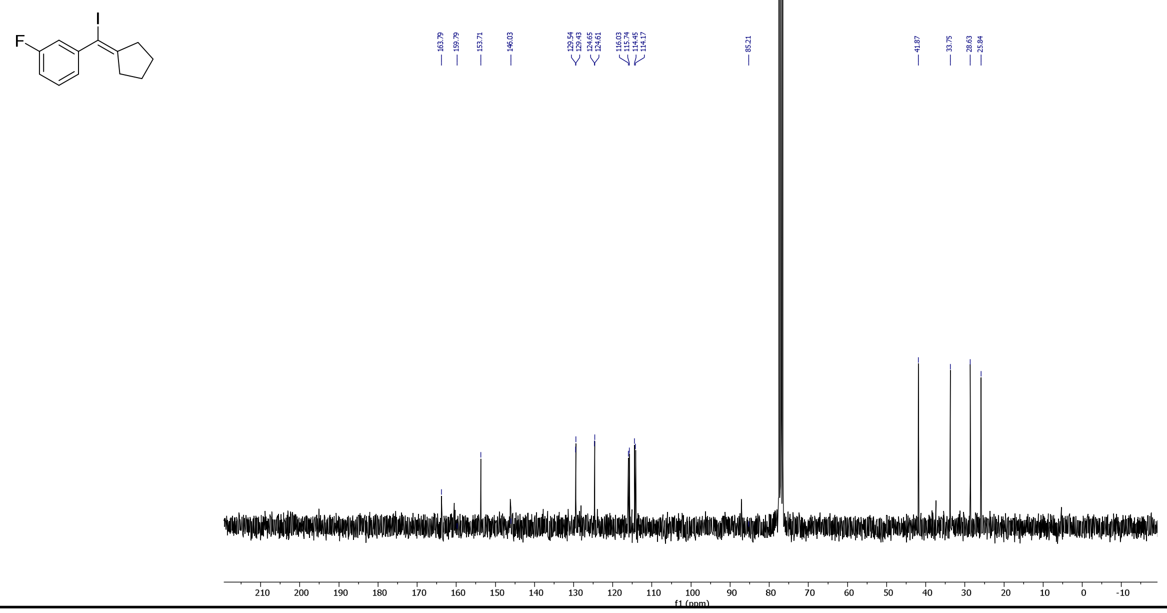

28b

^{19}F NMR (282 MHz, $CDCl_3$)

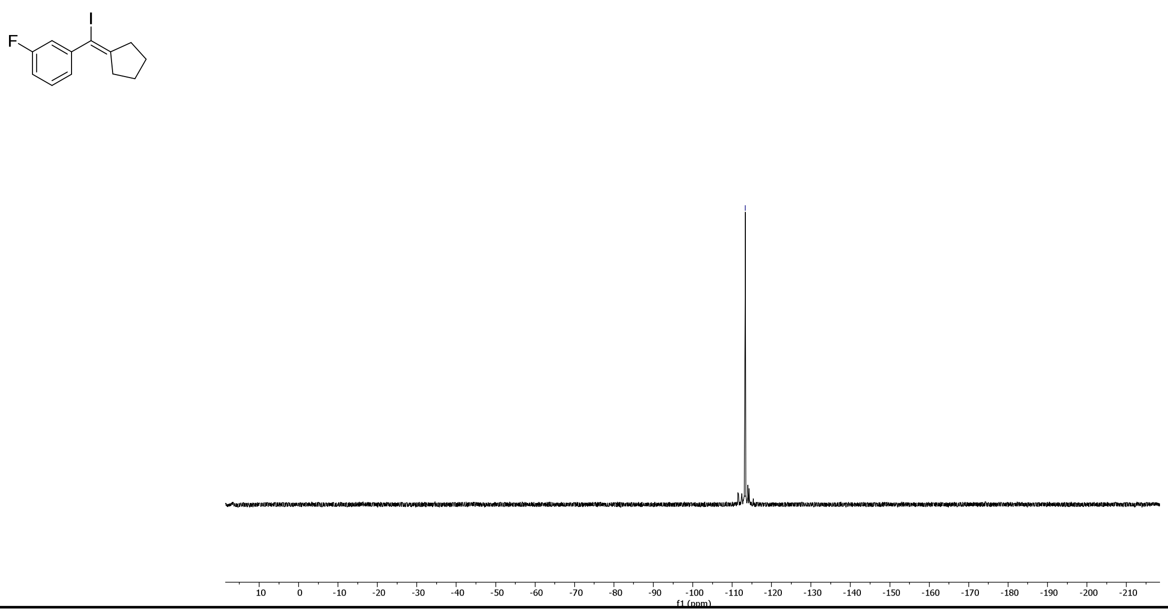

29b

^{1}H NMR (250 MHz, C_6D_6)

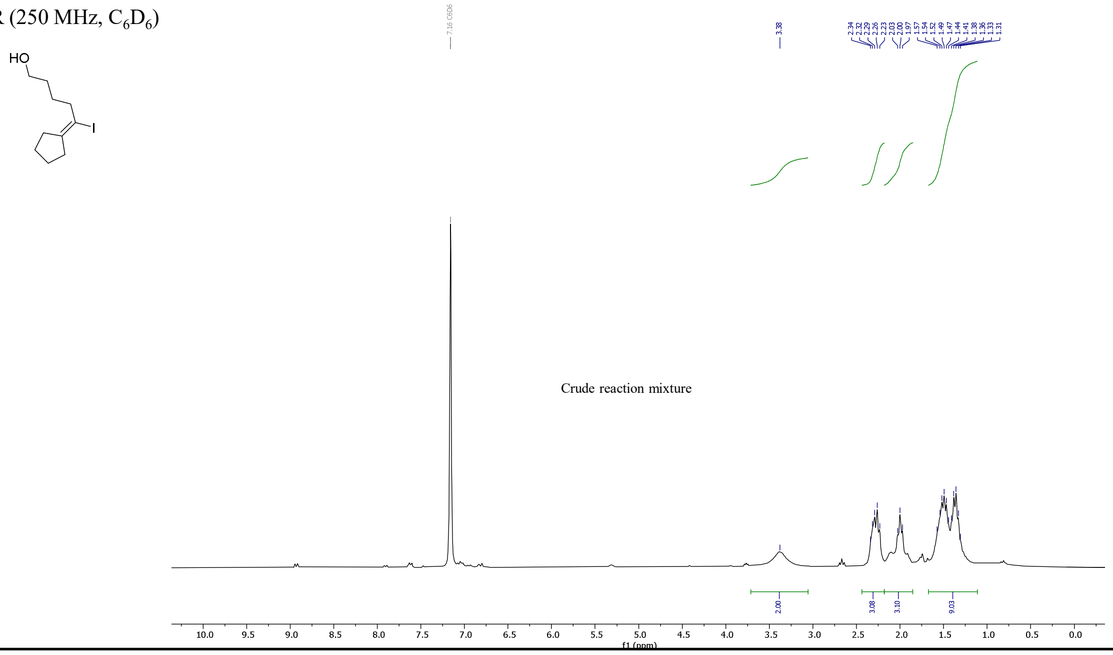

30b

^{1}H NMR (300 MHz, $CDCl_3$)

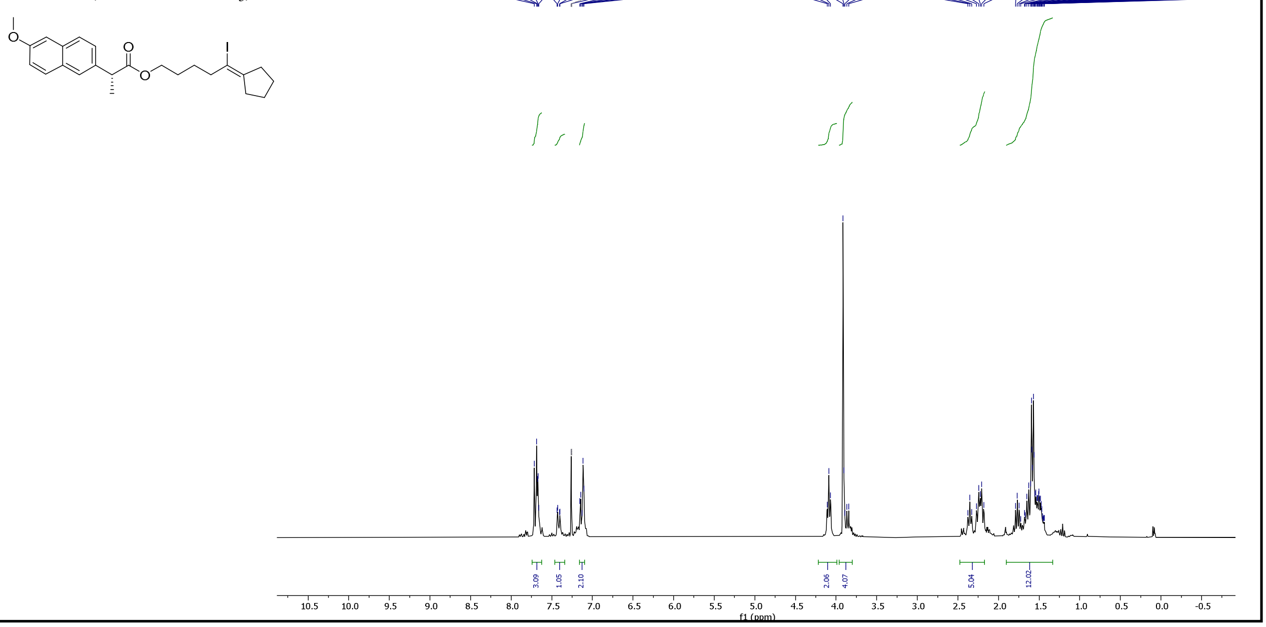

30b

^{13}C NMR (75 MHz, $CDCl_3$)

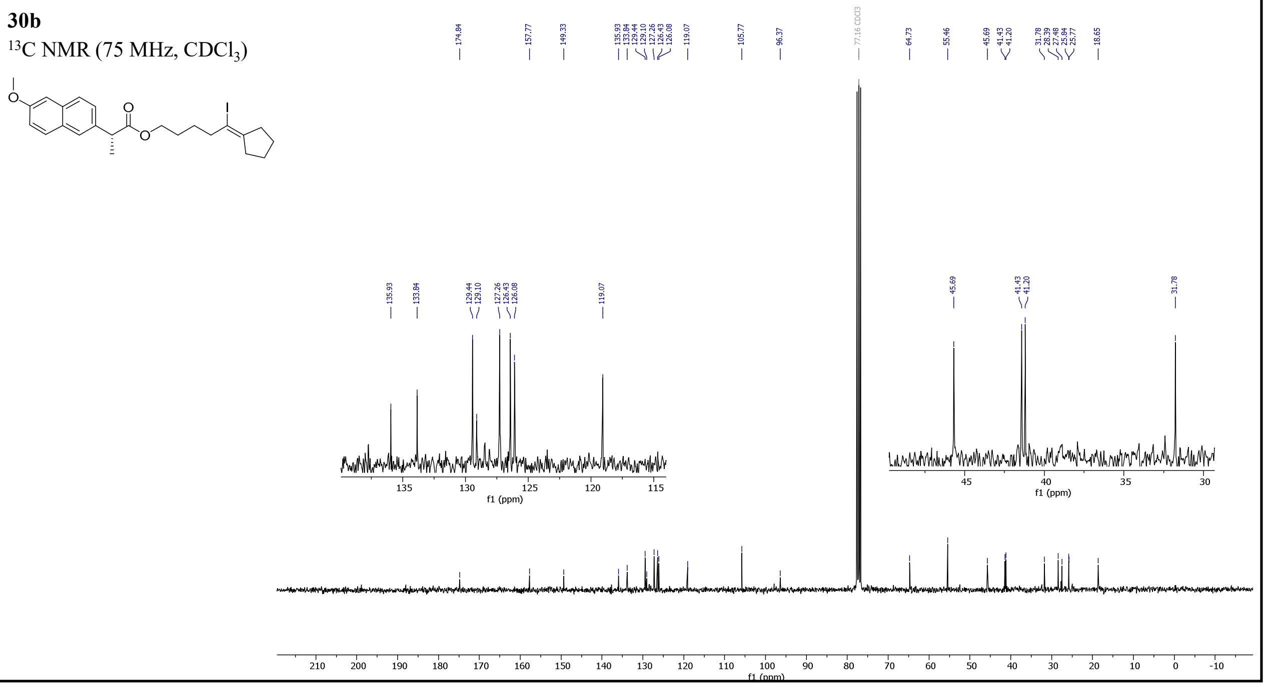

31b

^{1}H NMR (300 MHz, $CDCl_3$)

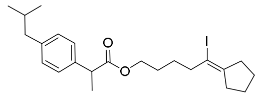

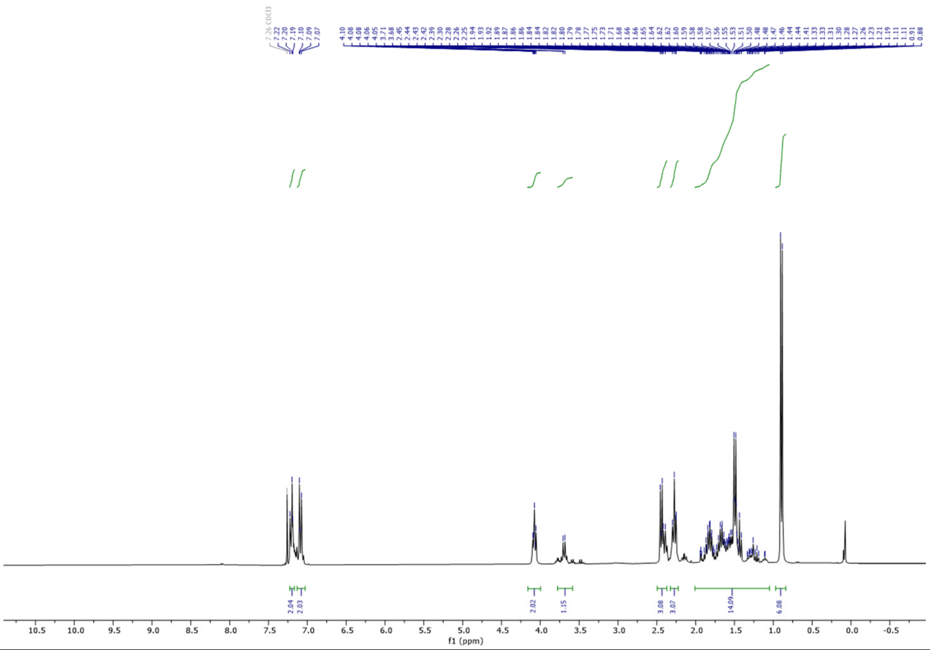

31b

^{13}C NMR (75 MHz, $CDCl_3$)

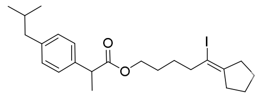

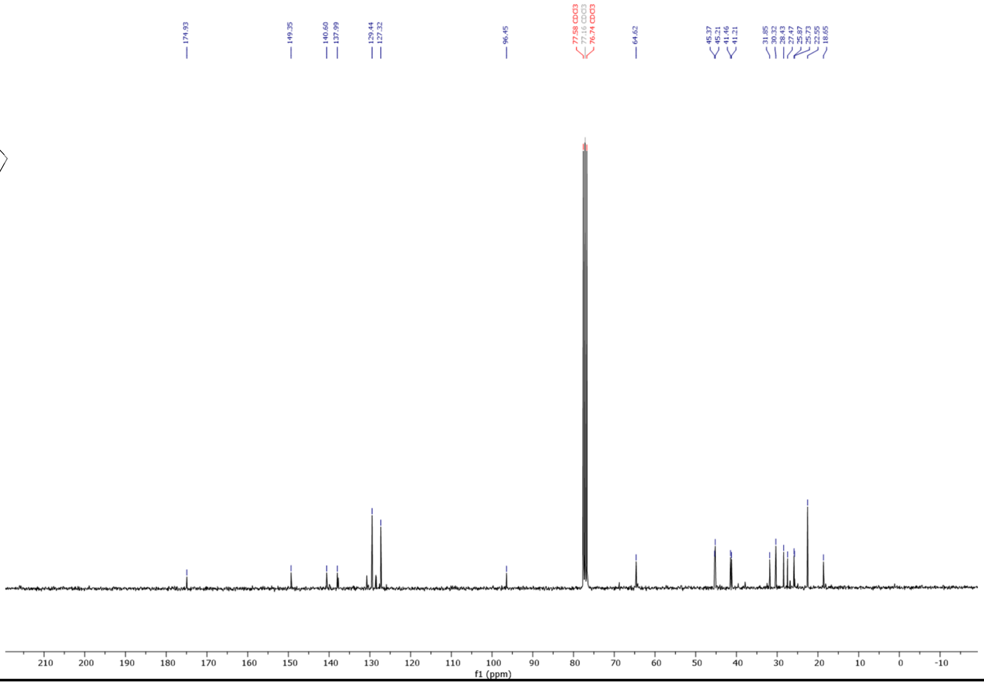

32b

^{1}H NMR (300 MHz, $CDCl_3$)

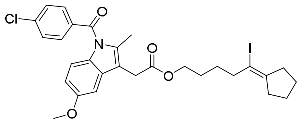

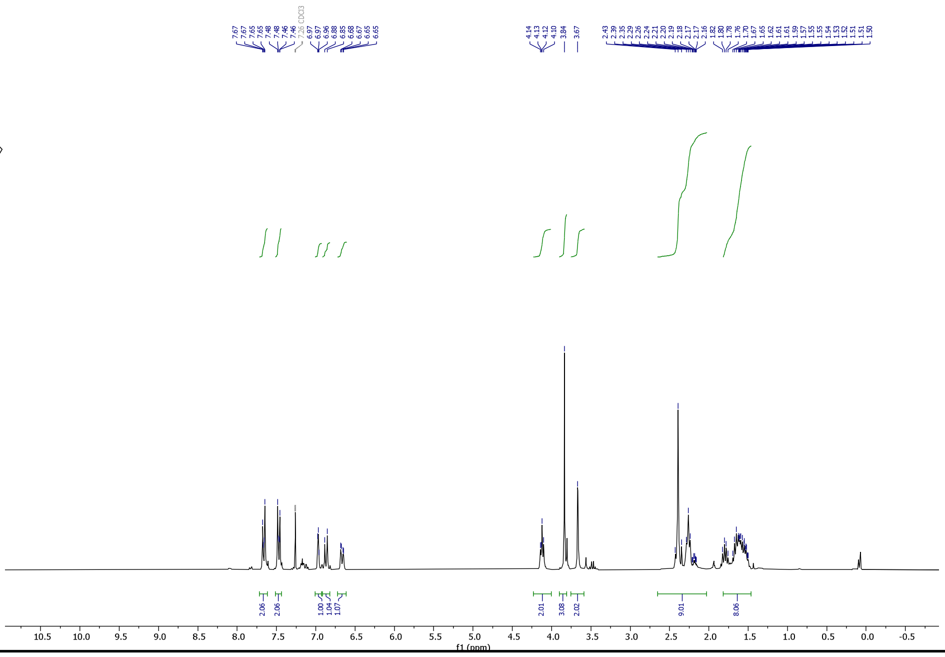

32b

^{13}C NMR (75 MHz, $CDCl_3$)

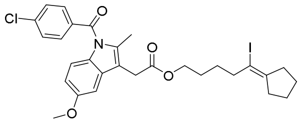

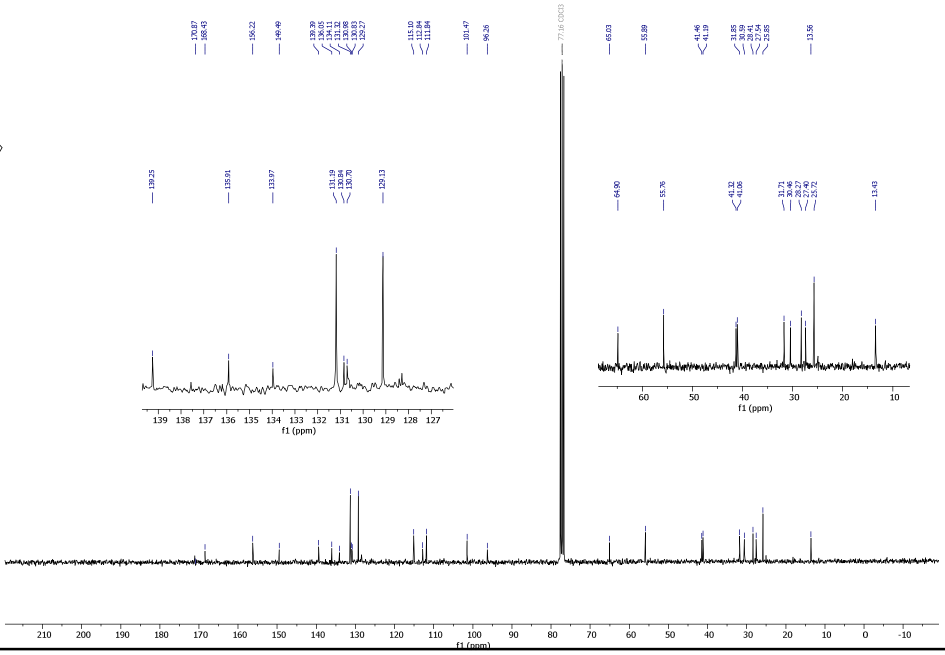

Supplement: Supplementary file 1 [file cs5c02812_si_001.pdf]
